# Oxidation of ethidium-based probes by biological radicals: mechanism, kinetics and implications for the detection of superoxide

**DOI:** 10.1038/s41598-020-75373-2

**Published:** 2020-10-29

**Authors:** Radosław Michalski, David Thiebaut, Bartosz Michałowski, Mehmet M. Ayhan, Micael Hardy, Olivier Ouari, Michał Rostkowski, Renata Smulik-Izydorczyk, Angelika Artelska, Andrzej Marcinek, Jacek Zielonka, Balaraman Kalyanaraman, Adam Sikora

**Affiliations:** 1 Institute of Applied Radiation Chemistry, Lodz University of Technology, 90-924 Lodz, Poland; 2 Aix Marseille Univ, CNRS, ICR, UMR 7273, 13013 Marseille, France; 3 Department of Chemistry, Gebze Technical University, 41400 Gebze Kocaeli, Turkey; 4 Department of Biophysics and Free Radical Research Center, Medical College of Wisconsin, Milwaukee, WI 53226 USA

**Keywords:** Physical chemistry, Fluorescent probes, Chemical biology, Small molecules

## Abstract

Hydroethidine (HE) and hydropropidine ($\hbox {HPr}^{+}$) are fluorogenic probes used for the detection of the intra- and extracellular superoxide radical anion ($\hbox {O}_{ {2}}^{\bullet -}$). In this study, we provide evidence that HE and $\hbox {HPr}^{+}$ react rapidly with the biologically relevant radicals, including the hydroxyl radical, peroxyl radicals, the trioxidocarbonate radical anion, nitrogen dioxide, and the glutathionyl radical, via one-electron oxidation, forming the corresponding radical cations. At physiological pH, the radical cations of the probes react rapidly with $\hbox {O}_{ {2}}^{\bullet -}$, leading to the specific 2-hydroxylated cationic products. We determined the rate constants of the reaction between $\hbox {O}_{ {2}}^{\bullet -}$ and the radical cations of the probes. We also synthesized N-methylated analogs of $\hbox {HPr}^{+}$ and HE which were used in mechanistic studies. Methylation of the amine groups was not found to prevent the reaction between the radical cation of the probe and the superoxide, but it significantly increased the lifetime of the radical cation and had a substantial effect on the profiles of the oxidation products by inhibiting the formation of dimeric products. We conclude that the N-methylated analogs of HE and $\hbox {HPr}^{+}$ may be used as a scaffold for the design of a new generation of probes for intra- and extracellular superoxide.

## Introduction

The superoxide radical anion ($\hbox {O}_{ {2}}^{\bullet -}$) is the primary reactive oxygen species (ROS) produced *in vivo*. It is formed in the process of one-electron reduction of molecular oxygen, mediated by enzymes such as NAD(P)H oxidases^1^ and xanthine oxidase^2^. It is also produced in mitochondria, e.g., by the electron transport chain^3^. The superoxide radical anion itself is not highly reactive toward most biomolecules, but it can serve as a precursor of biologically relevant oxidants, such as hydrogen peroxide ($\hbox {H}_{ {2}}\hbox {O}_{ {2}}$) and peroxynitrite ($\hbox {ONOO}^{-}$), initiating a cellular cascade of various oxidizing, nitrating and halogenating species^4–7^.

When reactive oxygen and nitrogen species (ROS and RNS) are produced in cells in amounts sufficient to overcome the antioxidant defense, a state of “oxidative stress” occurs^8^. Oxidative stress has been linked to several pathophysiological states, including cancer, inflammation, reperfusion injuries, and aging^9–11^. Therefore, it is essential to develop reliable probes and methods allowing for rigorous and accurate detection and characterization of ROS and RNS in biological samples. Among other approaches, fluorescent probes are used widely for ROS/RNS measurements, due mostly to their variety and versatility, as well as owing to the sensitivity of fluorescence-based techniques^12^.

**Figure 1:**
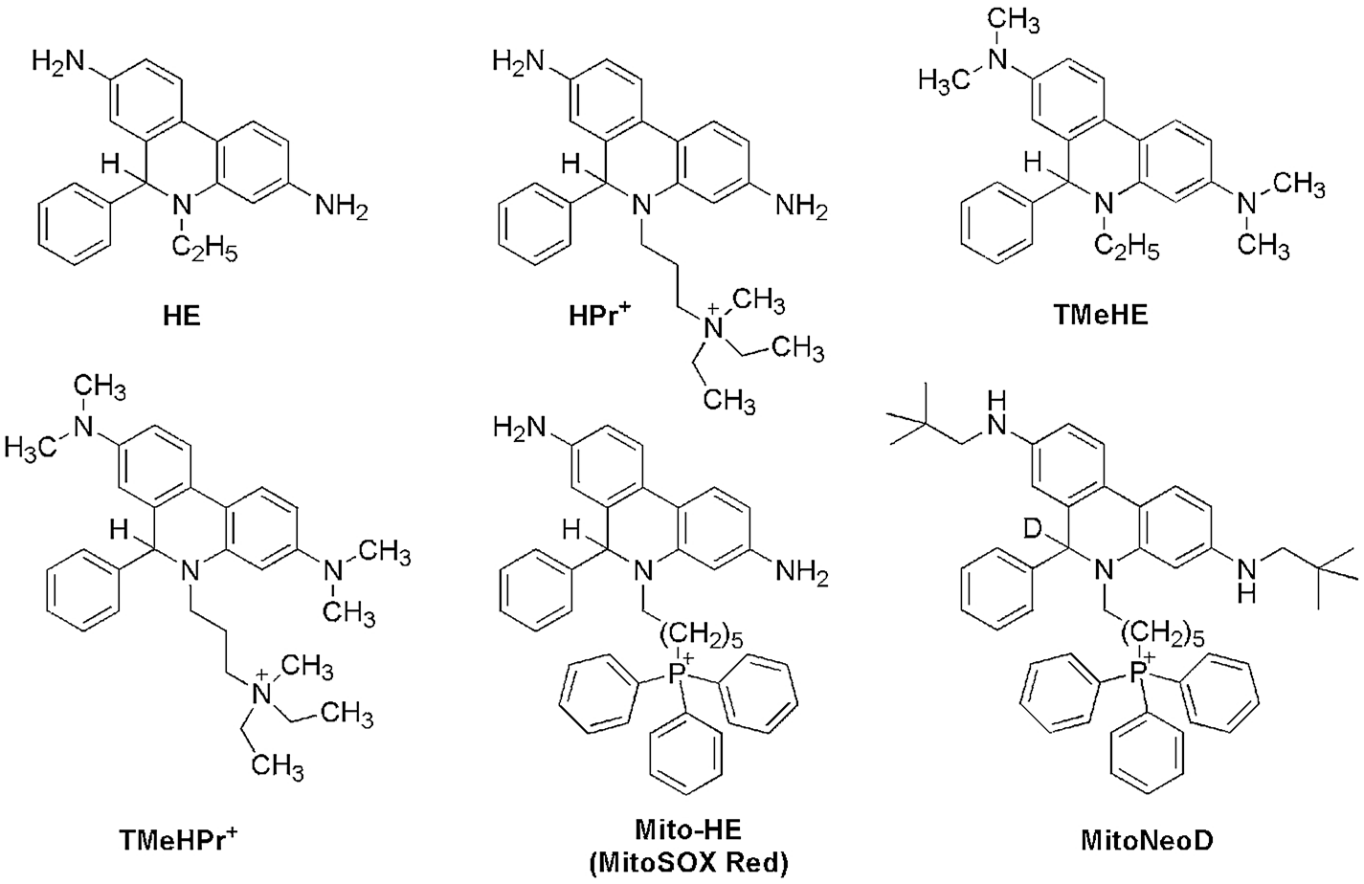
Chemical structures of hydroethidine (HE), hydropropidine ($\hbox {HPr}^{+}$), N,N,N’,N’-tetramethylhydroethidine (TMeHE), N,N,N’,N’-tetramethylhydropropidine ($\hbox {TMeHPr}^{+}$), mitochondria-targeted hydroethidine (Mito-HE), and mitochondria-targeted superoxide probe (MitoNeoD).

Of the fluorescent probes used for $\hbox {O}_{ {2}}^{\bullet -}$ detection^13–20^, hydroethidine (HE) has been found to be one of the most promising candidates. It has been applied in a variety of *in vitro* and *in vivo* systems, ranging from intracellular organelles to whole organs in live animals^21–26^. The triphenylphosphonium-linked derivative of HE, known as Mito-HE (or MitoSOX Red), has been synthesized in order to detect $\hbox {O}_{ {2}}^{\bullet -}$ produced in mitochondria^24^. Another derivative of HE used to detect $\hbox {O}_{ {2}}^{\bullet -}$ in isolated mitochondria and mouse heart mitochondria *in vivo* is MitoNeoD^27^. A cell membrane-impermeable analog of HE, hydropropidine ($\hbox {HPr}^{+}$), designed for the detection of extracellular $\hbox {O}_{ {2}}^{\bullet -}$^28^, was used successfully to identify Nox2 inhibitors by monitoring $\hbox {O}_{ {2}}^{\bullet -}$ production from activated NADPH oxidase directly by fluorescence in high-throughput screening assays^29,30^. Although HE, Mito-HE, MitoNeoD, and $\hbox {HPr}^{+}$, differ in their exact chemical structures (Fig. 1) and physicochemical properties, resulting in different subcellular distributions, their overall chemical reactivity is very similar^24,27,28,31^.

**Figure 2:**
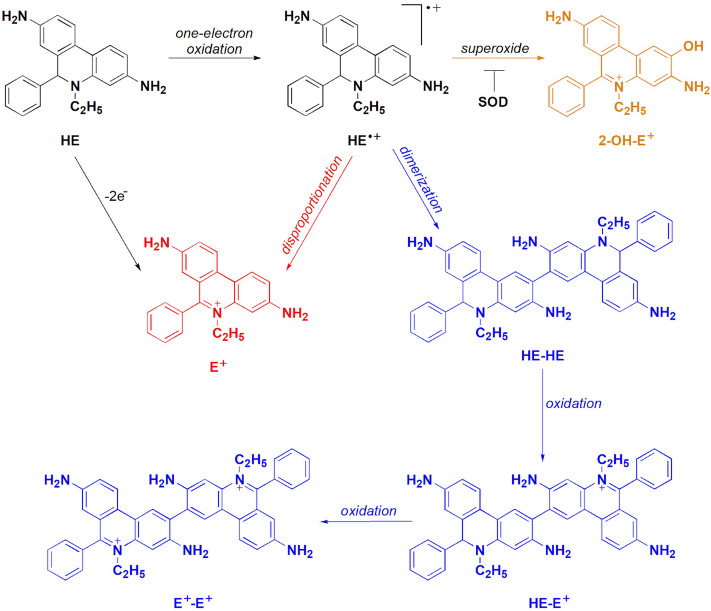
Oxidative chemistry of the HE probe.

The chemical reactivity of HE-based probes has been studied in some detail^28,31–37^, including their reaction kinetics and reactivity toward various one- and two-electron oxidants. The main and unequivocal advantage of using hydroethidine-based probes is the specific formation of 2-hydroxylated products in the reaction with $\hbox {O}_{ {2}}^{\bullet -}$, which are unique markers of superoxide production. The most extensively studied hydroethidine-based probe is HE, and a large amount of data are available concerning its reactivity, including its stoichiometry and oxidation mechanism^36,38,39^. According to the literature, in the presence of varying fluxes of $\hbox {O}_{ {2}}^{\bullet -}$ the only SOD-sensitive product formed from the reaction between HE and $\hbox {O}_{ {2}}^{\bullet -}$ is $\hbox {2-OH-E}^{+}$^39^. Irrespective of the one-electron oxidant present, the first step in the oxidation reaction of HE leads to the formation of an intermediate that is thought to be either a radical cation or an aromatic aminyl radical, which in the presence of $\hbox {O}_{ {2}}^{\bullet -}$ is subsequently converted into $\hbox {2-OH-E}^{+}$. In the absence of the superoxide, one-electron oxidation of HE results in the formation of $\hbox {E}^{+}$ and characteristic dimeric products, such as HE-HE, HE-$\hbox {E}^{+}$, and $\hbox {E}^{+}$-$\hbox {E}^{+}$, the distribution of which depends on the reaction conditions (Fig. 2). The same has been shown to be true for the $\hbox {HPr}^{+}$ probe^28^. Although much work has focused on elucidating the oxidation mechanism of HE-based probes, more comprehensive knowledge of their chemical reactivity toward biologically relevant oxidants is needed for their reliable use in biological systems and for accurate interpretation of experimental data.

Here, we present a detailed mechanistic study of the one-electron oxidation of hydropropidine, a water-soluble analogue of HE. We report the kinetics of reactions between $\hbox {HPr}^{+}$ and the biologically relevant oxidants, such as glutathionyl radical ($\hbox {GS}^{\bullet }$), nitrogen dioxide ($^{\bullet }\hbox {NO}_{ {2}}$), and trioxidocarbonate radical anion ($\hbox {CO}_{ {3}}^{\bullet -}$), as well as the kinetics of the reaction of the probe-derived radical cation ($\hbox {HPr}^{\bullet 2+}$) with $\hbox {O}_{ {2}}^{\bullet -}$. The results are complemented by spectroscopic characterization of the one-electron oxidation products of $\hbox {HPr}^{+}$ and HE in cryogenic matrices, along with quantum mechanical calculations. The mechanistic conclusions are supported by detailed analyses of the oxidation products. We also introduce novel N-methylated HE and $\hbox {HPr}^{+}$ derivatives (Fig. 1), N,N,N’,N’-tetramethylhydroethidine (TMeHE) and N,N,N’,N’-tetramethyhydropropidine ($\hbox {TMeHPr}^{+}$), both for mechanistic studies and as a chemical scaffold for the development of new HE-based probes for $\hbox {O}_{ {2}}^{\bullet -}$.

## Results

### Kinetic studies using pulse radiolysis

#### Reaction of $\hbox {HPr}^{+}$ and $\hbox {HE}$ with $^{\bullet }{\text{NO}}_{ {2}}$ and $\hbox {CO}_{ {3}}^{\bullet -}$

**Figure 3:**
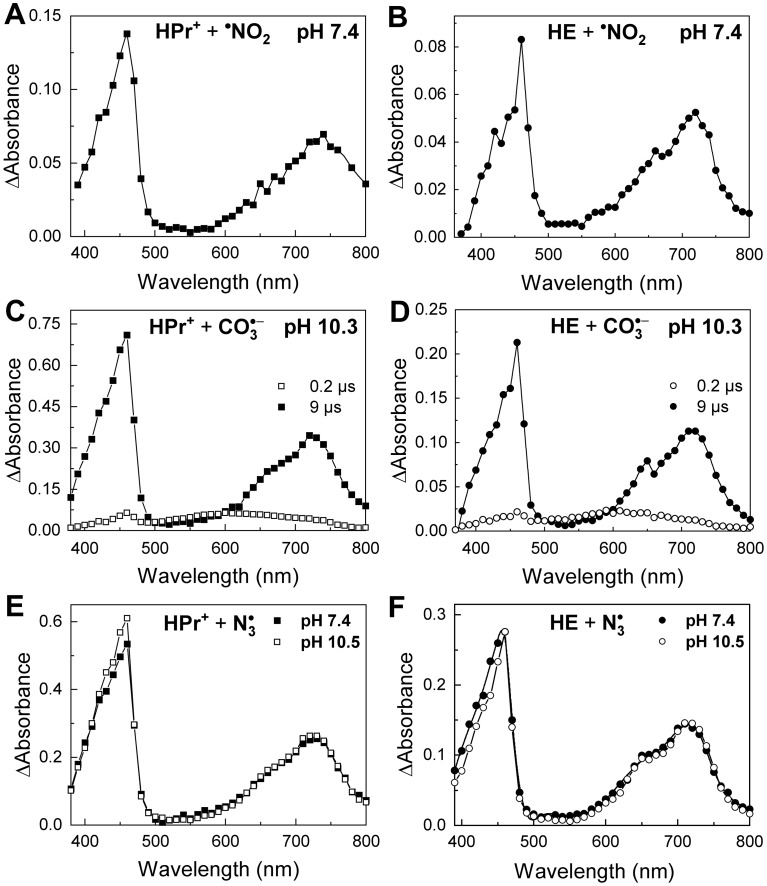
Absorption spectra of one-electron oxidation products of $\hbox {HPr}^{+}$ and HE. (**A**) Transient absorption spectrum recorded after pulse radiolysis of $\hbox {N}_{ {2}}$-saturated solutions of 0.1 M $\hbox {NaNO}_{ {3}}$, 1 M *t*-BuOH, and 50 mM phosphate buffer at pH 7.4 containing 50 $\upmu$M $\hbox {HPr}^{+}$ recorded 25 $\upmu$s after the electron pulse. Radiation dose: 62 Gy. (**B**) same as (**A**) but the solution contained 75 $\upmu$M HE. Radiation dose: 50 Gy. (**C**) Transient absorption spectra obtained by pulse radiolysis of 50 $\upmu$M $\hbox {HPr}^{+}$ in $\hbox {N}_{ {2}}$O-saturated aqueous solution containing 0.25 M $\hbox {Na}_{ {2}}\hbox {CO}_{ {3}}$ and 0.25 M $\hbox {NaHCO}_{ {3}}$ (pH 10.3). Spectra recorded (open squares) 0.2 $\upmu$s and (solid squares) 9 $\upmu$s after the electron pulse. Radiation dose: 53 Gy. (**D**) same as (**C**) but the solution contained 100 $\upmu$M HE instead of $\hbox {HPr}^{+}$ and 10% vol. $\hbox {CH}_{ {3}}\hbox {CN}$. Spectra recorded (open circles) 0.2 $\upmu$s and (solid circles) 9 $\upmu$s after the electron pulse. Radiation dose 52 Gy. (**E**) Transient absorption spectra recorded after pulse radiolysis of $\hbox {HPr}^{+}$ (50 $\upmu$M) in $\hbox {N}_{ {2}}$O-saturated solution containing phosphate buffer (50 mM) with $\hbox {NaN}_{ {3}}$ (0.1 M). Spectra recorded (solid squares) at pH 7.4, radiation dose 54 Gy and (open squares) at pH 10.5, radiation dose 54 Gy (the solution was adjusted with 0.1 M NaOH to pH 10.5). (**F**) same as (**E**) but the solution contained HE instead of $\hbox {HPr}^{+}$ and 10% $\hbox {CH}_{ {3}}\hbox {CN}$ (solid circles—75 $\upmu$M HE, pH 7.4, radiation dose 65 Gy; open circles—100 $\upmu$M HE, pH 10.5, radiation dose 63 Gy). All samples were 1 cm thick.

Decomposition of peroxynitrite in the presence of $\hbox {CO}_{ {2}}$ leads to the formation of $^{\bullet }\hbox {NO}_{ {2}}$ and $\hbox {CO}_{ {3}}^{\bullet -}$^4^. To study the reaction of $^{\bullet }\hbox {NO}_{ {2}}$ with the $\hbox {HPr}^{+}$ probe, $^{\bullet }\hbox {NO}_{ {2}}$ was generated by pulse radiolysis of an aqueous solution of $\hbox {HPr}^{+}$, as described in detail in the Supplementary Information (SI). The reaction of $^{\bullet }\hbox {NO}_{ {2}}$ and $\hbox {HPr}^{+}$ led to the formation of a product with two characteristic absorption bands: a narrow band located at 460 nm and a broad band with a maximum at 720 nm (Fig. 3A). Similar absorption bands were observed during the reaction of $^{\bullet }\hbox {NO}_{ {2}}$ with HE instead of $\hbox {HPr}^{+}$ (Fig. 3B). The second-order rate constants determined for the reaction of $^{\bullet }\hbox {NO}_{ {2}}$ with $\hbox {HPr}^{+}$ and HE are equal to $(6.5\pm 0.3) \times 10^8$ $\hbox {M}^{-1}\hbox {s}^{-1}$ and $(6.8\pm 0.3)\times 10^8$ $\hbox {M}^{-1}\hbox {s}^{-1}$, respectively (Supplementary Table S1 and Fig. S1). We have also carried out the kinetic simulations for the reaction of $\hbox {HPr}^{+}$ with both oxidants that support the experimental data.  The used kinetic models are presented in the SI.

The oxidation of $\hbox {HPr}^{+}$ and HE by $\hbox {CO}_{ {3}}^{\bullet -}$ was also studied. The reaction of both probes with $\hbox {CO}_{ {3}}^{\bullet -}$ was performed at pH 10.3 to ensure the fast and complete conversion of the hydroxyl radical produced during radiolysis into $\hbox {CO}_{ {3}}^{\bullet -}$ (see SI). Initially, after the electron pulse, a broad weak absorption band around 600 nm appeared (Fig. 3C,D), consistent with the formation of $\hbox {CO}_{ {3}}^{\bullet -}$^40^. Over the course of the reaction, this absorption band was replaced by the absorption bands of the oxidation product of $\hbox {HPr}^{+}$ (Fig. 3C) when the solute was $\hbox {HPr}^{+}$ or by the bands for the oxidation product of HE (Fig. 3D) when HE was present in the reaction mixture. Both $\hbox {HPr}^{+}$ and HE were oxidized by $\hbox {CO}_{ {3}}^{\bullet -}$ at pH 10.3. The second-order rate constants are equal to $(4.8\pm 0.6)\times 10^9$ $\hbox {M}^{-1}\hbox {s}^{-1}$ and $(4.6\pm 0.2)\times 10^9$ $\hbox {M}^{-1}\hbox {s}^{-1}$, respectively (Supplementary Table S1 and Fig. S1). We have also performed the kinetic simulations for the reaction of $\hbox {HPr}^{+}$ with $\hbox {N}_{ {3}}^{\bullet }$ and the data are available in SI.

#### Oxidation of $\hbox {HPr}^{+}$ and $\hbox {HE}$ by $\hbox {N}_{ {3}}^{\bullet }$ at different $\hbox {pH}$

To confirm that both $^{\bullet }\hbox {NO}_{ {2}}$ and $\hbox {CO}_{ {3}}^{\bullet -}$ react with the probes *via* one-electron transfer, we performed additional studies at two different pH values using the azidyl ($\hbox {N}_{ {3}}^{\bullet }$) radical as a strong, but selective one-electron oxidant. The transient absorption spectra obtained from the reaction of $\hbox {HPr}^{+}$ with the $\hbox {N}_{ {3}}^{\bullet }$ radical at pH 7.4 and pH 10.5 are presented in Fig. 3E. The same spectrum was recorded for both pH values, with strong absorption observed around 460 nm and an additional broad band of lower intensity with its maximum observed at 720 nm. The second-order rate constant of the reaction between $\hbox {HPr}^{+}$ and $\hbox {N}_{ {3}}^{\bullet }$, $k = (4.8\pm 0.2)\times 10^9$ $\hbox {M}^{-1}\hbox {s}^{-1}$ (Supplementary Table S1 and Fig. S1), was determined from the dependence of the kinetics of the increase in absorption at 460 nm on the $\hbox {HPr}^{+}$ concentration. Figure 3F shows the absorption spectra of the transient species formed during the reaction of $\hbox {N}_{ {3}}^{\bullet }$ with HE at pH 7.4 and pH 10.5. Similar to the case of $\hbox {HPr}^{+}$, the same species was observed at both pH values. The determined rate constant for the reaction of the $\hbox {N}_{ {3}}^{\bullet }$ radical with HE, at pH 7.4 is equal to $(4.2\pm 0.3)\times 10^9$ $\hbox {M}^{-1}\hbox {s}^{-1}$ (Supplementary Table S1 and Fig. S1).

#### Oxidation of $\hbox {HPr}^{+}$ and $\hbox {HE}$ by $\hbox {HO}^{\bullet }$ and $\hbox {Br}_{ {2}}^{\bullet -}$

We used the hydroxyl radical ($\hbox {HO}^{\bullet }$) and the dibromide radical anion ($\hbox {Br}_{ {2}}^{\bullet -}$) to better characterize the reactivity of the studied probes toward one-electron oxidants. Pulse radiolytic generation of the $\hbox {HO}^{\bullet }$ and $\hbox {Br}_{ {2}}^{\bullet -}$ radicals in the presence of $\hbox {HPr}^{+}$ led to the appearance of characteristic spectra observed previously during the reaction of the probe with $^{\bullet }\hbox {NO}_{ {2}}$, $\hbox {CO}_{ {3}}^{\bullet -}$, and $\hbox {N}_{ {3}}^{\bullet }$ oxidants (Fig. 3A,C,E). The hydroxyl radical is known to be a non-selective oxidant that reacts with aromatic compounds by electron transfer, hydrogen atom abstraction, and/or addition to the double bond. Due to the similarity of the observed spectrum for $\hbox {HO}^{\bullet }$-induced oxidation product to the other spectra obtained with selective one-electron oxidants, it is reasonable to assume that the $\hbox {HPr}^{+}$ probe reacts with $\hbox {HO}^{\bullet }$ primarily *via* electron transfer. This has been previously reported for HE^35^.

The rate constants for the reactions of $\hbox {HO}^{\bullet }$ and $\hbox {Br}_{ {2}}^{\bullet -}$ with $\hbox {HPr}^{+}$ were found to be equal to $(1.2\pm 0.1)\times 10^{10}$ $\hbox {M}^{-1}\hbox {s}^{-1}$ and $(3.9\pm 0.2)\times 10^9$ $\hbox {M}^{-1}\hbox {s}^{-1}$, respectively (Supplementary Table S1).

In the case of HE, we were unable to determine the appropriate rate constant with $\hbox {HO}^{\bullet }$, due to its side reactions with acetonitrile, which was used to improve the solubility of the probe in aqueous solution. The rate constant for the reactions of HE with $\hbox {Br}_{ {2}}^{\bullet -}$ was found to be equal to $(3.7\pm 0.1)\times 10^9$ $\hbox {M}^{-1}\hbox {s}^{-1}$ (Supplementary Table S1).

#### Reaction of $\hbox {GS}^{\bullet }$ and $\hbox {CysS}^{\bullet }$ with $\hbox {HPr}^{+}$ and $\hbox {HE}$

Under conditions of oxidative stress, initial scavenging of free radicals by reduced glutathione or cysteine leads to the formation of the thiyl radical, which reactivity towards the redox probes should also be considered. Therefore, we generated the biologically relevant thiyl radicals from glutathione and cysteine, abbreviated as $\hbox {GS}^{\bullet }$ and $\hbox {CysS}^{\bullet }$, respectively, to monitor their reaction with the $\hbox {HPr}^{+}$ and HE probes. One should note that $\hbox {GS}^{\bullet }$ and $\hbox {CysS}^{\bullet }$ exist in equilibrium with their carbon-centered radicals as reported by Schöneich and coworkers^41^. Although this equilibrium is shifted to the side of the sulfur-centered radicals and is rather fast^42^, it may affect the reaction kinetics, and thus the determined rate constants should be treated as the “apparent” values.

In the presence of $\hbox {HPr}^{+}$ or HE, the build-up of the absorption bands characteristic for their radical cations was completed within 30 $\upmu$s after pulse irradiation (Supplementary Fig. S2). The second-order rate constants of the $\hbox {GS}^{\bullet }$ reaction with $\hbox {HPr}^{+}$ or HE at pH 7.4 were found to be $(3.8\pm 0.3)\times 10^8$ $\hbox {M}^{-1}\hbox {s}^{-1}$ and $(2.9\pm 0.1)\times 10^8$ $\hbox {M}^{-1}\hbox {s}^{-1}$, respectively (Supplementary Fig. S2 and Table S1). The second-order rate constants of the $\hbox {CysS}^{\bullet }$ radical reactions with both probes have been determined to be equal to $(2.4\pm 0.1)\times 10^8$ $\hbox {M}^{-1}\hbox {s}^{-1}$ for $\hbox {HPr}^{+}$ and $(4.3\pm 0.2)\times 10^8$ $\hbox {M}^{-1}\hbox {s}^{-1}$ for HE (Supplementary Fig. S2 and Table S1).

#### Reactivity of $\hbox {HE}$ toward peroxyl radicals

**Figure 4:**
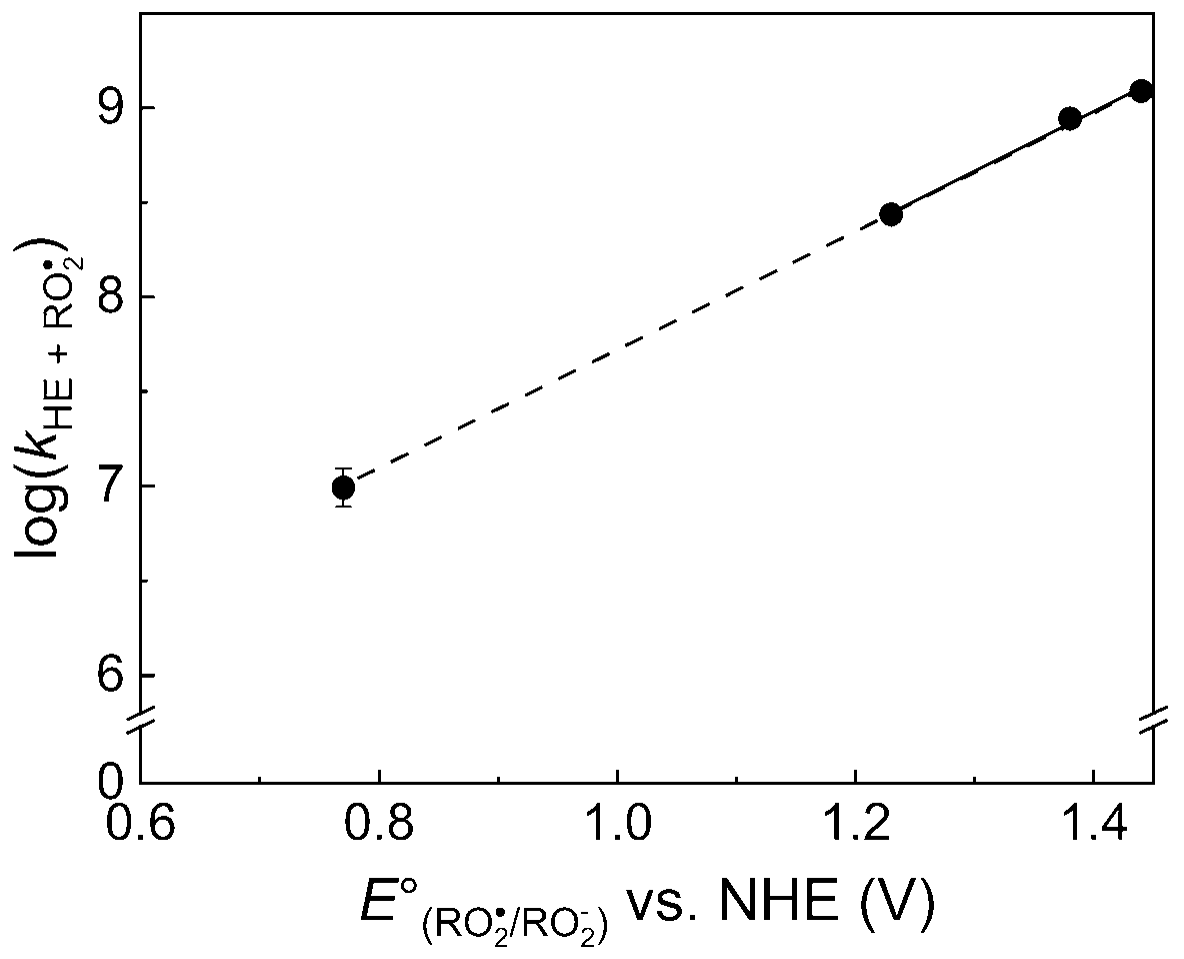
Relationship between the decimal logarithm of the second-order rate constants [log(k(HE + $\hbox {ROO}^{\bullet }$))] and the standard electrode potentials of peroxyl radicals [$\hbox {E}^\circ (\hbox {ROO}^{\bullet }/ \hbox {ROO}^{-})$], the kinetic and thermodynamic parameters of the reaction between HE and selected peroxyl radicals. The dashed line shows the extrapolation of the relationship to the value of the standard electrode potential of $\hbox {HO}_{ {2}}^{\bullet }$ (0.77 V). The standard electrode potential of the $\hbox {HO}_{ {2}}^{\bullet }/\hbox {HO}_{ {2}}^{-}$ couple was calculated using the pKa value of hydrogen peroxide (11.7), the standard electrode potential of the $\hbox {HO}_{ {2}}^{\bullet }$, $\hbox {H}^{+}$/$\hbox {H}_{ {2}}\hbox {O}_{ {2}}$ couple (1.46 V)^49^, and 1 M concentration of $\hbox {H}^{+}$ (pH 0), according to the standard state conditions^49^. The standard electrode potentials were taken from the literature^71^.

Peroxyl-type radicals are another class of strong one-electron oxidants of biological relevance. We used a series of pulse-generated model peroxyl radicals to study the reactivity of HE towards these oxidants. The chloromethylperoxyl radical reacted with HE to produce a transient species possessing the characteristic absorption spectra observed in the reaction of HE with the other used oxidants (Supplementary Fig. S3). The second-order rate constants determined at pH 7.4 for the reaction of HE with $\hbox {CCl}_{{3}}\hbox {OO}^{\bullet }$, $\hbox {CHCl}_{ {2}}\hbox {OO}^{\bullet }$, and $\hbox {CH}_{ {2}}\hbox {ClOO}^{\bullet }$ radicals are equal to $(1.23\pm 0.02)\times 10^9$, $(8.8\pm 0.1)\times 10^8$, and $(2.7\pm 0.1)\times 10^8$ $\hbox {M}^{-1}\hbox {s}^{-1}$, respectively (Fig. 4 and Supplementary Table S2). This indicates that the rate constant of the reaction can be correlated with the strength (standard electrode potential) of the oxidant. Extrapolating the dependence of log k on the standard standard electrode potentials of the oxidants to the value calculated for the hydridodioxygen($^{\bullet }$) ($\hbox {HO}_{ {2}}^{\bullet }$, also known as the hydroperoxyl radical), enabled us to estimate the rate constant for the reaction of this oxidant with HE ($\sim 1\times 10^7$ $\hbox {M}^{-1}\hbox {s}^{-1}$, Fig. 4 and Supplementary Table S2).

### Low-temperature radiolysis and quantum-mechanical calculations

**Figure 5:**
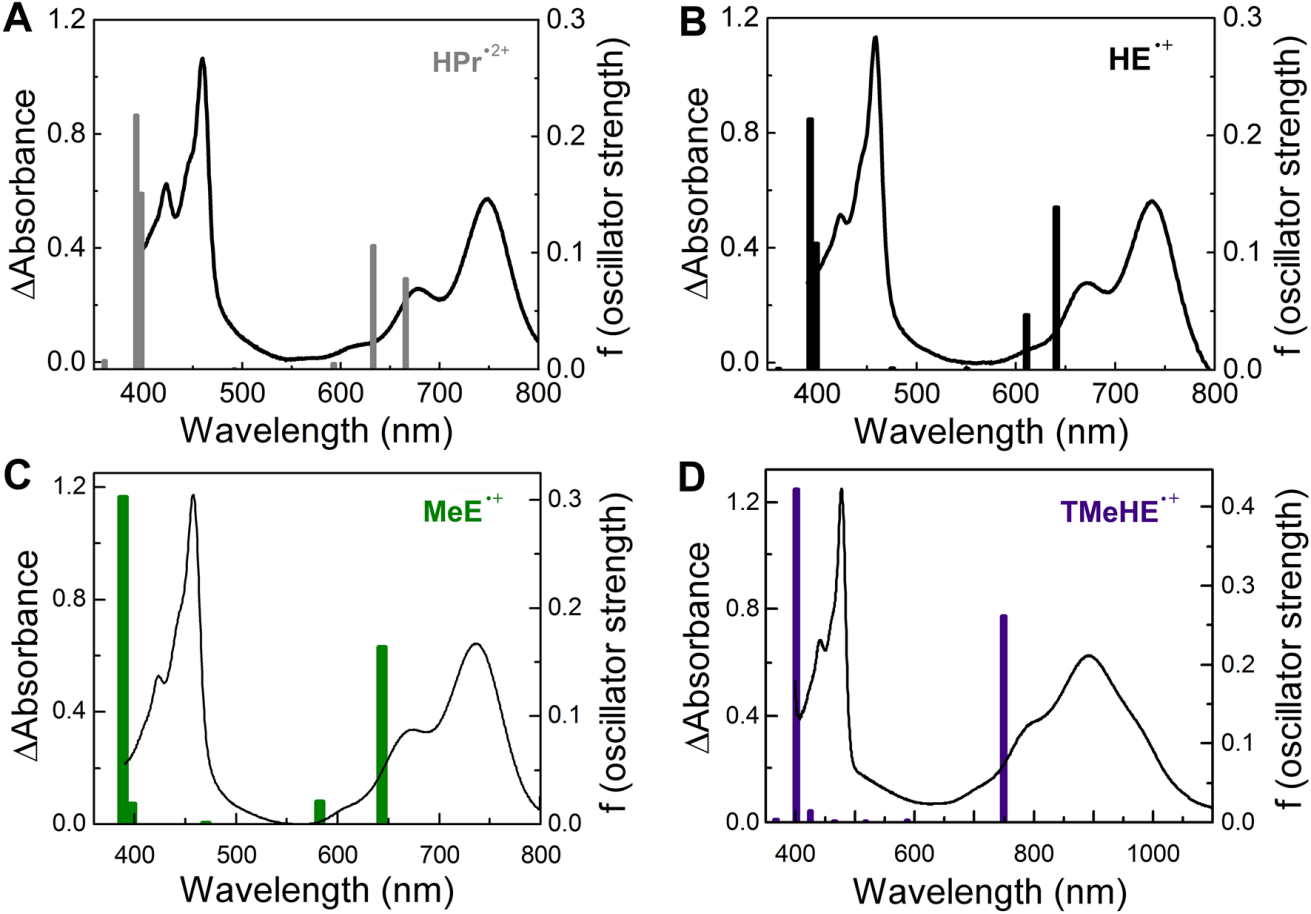
Comparison between the experimental and calculated spectra of the one-electron oxidation products of $\hbox {HPr}^{+}$, HE, MeE, and TMeHE. (**A**–**D**, respectively). Electronic absorption spectra obtained by radiolysis of $\hbox {HPr}^{+}$ (2 mM), HE (8.6 mM), MeE (10 mM), and TMeHE (10 mM), respectively, embedded in low temperature glass of $\hbox {BMIM}^{+}\hbox{PF}_{ {6}}^{-}:\hbox {CH}_{ {2}}\hbox {Cl}_{ {2}}$ (1:1, v/v) at 77 K. (the sample of $\hbox {HPr}^{+}$ received a radiation dose of 5.8 kGy, HE received a radiation dose of 27 kGy, the samples of MeE and TMeHE received a radiation dose of 36 kGy, all samples were $\sim$ 1 mm thick.) Excitation energies of $\hbox {HPr}^{\bullet 2+}$, $\hbox {HE}^{\bullet +}$, $\hbox {MeE}^{\bullet +}$, and $\hbox {TMeHE}^{\bullet +}$ obtained from TD-DFT quantum mechanical calculations are shown as vertical bars.

Due to its inhibitory effect on recombination and fragmentation processes, a rigid frozen matrix allows for direct observation of the primary oxidation products of solute molecules, such as radical cations. Therefore, additional experiments were performed to ensure correct identification of the transients observed in the pulse radiolysis experiments at room temperature. Upon irradiation of a frozen solution of $\hbox {HPr}^{+}$ in a mixture of 1-butyl-3-methylimidazolium hexafluorophosphate and methylene chloride ($\hbox {BMIM}^{+}\hbox {PF}_{ {6}}^{-}:\hbox {CH}_{ {2}}\hbox {Cl}_{ {2}}$, 1:1, v/v) at 77 K, a UV–Vis absorption spectrum was recorded with a strong absorption band located around 460 nm and minor absorption bands at 678 and 748 nm. This spectrum was provisionally assigned to the radical cation of $\hbox {HPr}^{+}$ ($\hbox {HPr}^{\bullet 2+}$) (Fig. 5A). Irradiation of HE embedded in the frozen mixture of $\hbox {BMIM}^{+}\hbox{PF}_{ {6}}^{-}:\hbox {CH}_{ {2}}\hbox {Cl}_{ {2}}$ led to the formation of a species possessing the same absorption band profile, and this product was ascribed to the radical cation of HE ($\hbox {HE}^{\bullet +}$) (Fig. 5B).

To further confirm this assignment and exclude the possibility of rapid deprotonation of the radical cations formed during irradiation of the cryogenic matrices, we also performed spectroscopic characterization of the radical cations of methylethidine (MeE) and TMeHE in the low-temperature matrices. MeE bears a methyl group in position 6, in the place of a hydrogen atom in HE. In contrast to HE, the formation of an ethidium cation is not possible following one-electron oxidation. The species formed upon irradiation of the frozen solution of MeE in a $\hbox {BMIM}^{+}\hbox{PF}_{ {6}}^{-}:\hbox {CH}_{ {2}}\hbox {Cl}_{ {2}}$ mixture (1:1, v/v) at 77 K, was characterized by a UV–Vis spectrum with a strong absorption band located around 460 nm and minor absorption bands at 670 nm and 740 nm. This spectrum was assigned to the radical cation of MeE ($\hbox {MeE}^{\bullet +}$) (Fig. 5C). To test the possibility of deprotonation of the radical cation from the exocyclic amine groups, analogous experiments were performed for TMeHE (Fig. 5D). For MeE and TMeHE, similar electronic absorption spectra to those obtained upon oxidation of $\hbox {HPr}^{+}$ and HE were recorded. Interestingly, for the radical cation of TMeHE ($\hbox {TMeHE}^{\bullet +}$) the long-wavelength absorption band was red-shifted compared to those for $\hbox {HPr}^{\bullet 2+}$, $\hbox {HE}^{\bullet +}$ and $\hbox {MeE}^{\bullet +}$.

The results of cryogenic measurements were complemented by quantum mechanical calculations of electron spin densities and excited-state transitions. The density functional theory (DFT) calculations for the radical cations of $\hbox {HPr}^{+}$, HE, and their analogs were performed at the B3LYP/6-311+G(d,p) level. The geometry and electronic structures of the radical cations were calculated and the absorption spectra of the species were computed. The results are reported in Fig. 5 and Supplementary Fig. S4, and compared with the experimental data from radiolysis of frozen glasses in Supplementary Table S3. The results of TD-DFT calculations for $\hbox {HPr}^{\bullet 2+}$, $\hbox {HE}^{\bullet +}$, $\hbox {MeE}^{\bullet +}$, and $\hbox {TMeHE}^{\bullet +}$ are in reasonable agreement with the experimental data obtained from cryogenic measurements (Fig. 5). The shifts of the absorption bands ($\sim$0.25–0.5 eV) are typical for these types of calculations and for the B3LYP functional^43^, and can also be partially attributed to the solvent effect, as the calculations were performed *in vacuo*. Moreover, the ratios of the intensities of the two absorption bands in the experimental spectra and of the calculated transitions remain in agreement. It should also be noted, that the red shift for the long-wavelength absorption band of $\hbox {TMeHE}^{\bullet +}$ is in agreement with TD-DFT-based predictions of the effect of methylation of the $-\hbox {NH}_{ {2}}$ groups on the electronic transitions in the radical cation. Thus, the spectra obtained from TD-DFT calculations are in agreement with the assignment of the species observed in radiolytic studies to the radical cations of HE, $\hbox {HPr}^{+}$, MeE, and TMeHE.

Analysis of the Mulliken atomic spin densities of the $\hbox {HPr}^{+}$ and HE radical cations reveals that the highest spin density is located at the C-2 and C-12 carbon atoms (Supplementary Fig. S4 and Table S3). Similar spin distributions were calculated for the radical cations of MeE and TMeHE (Supplementary Fig. S4 and Table S3).

### Stabilization of the probe radical cation by methylation of the amine groups

One-electron oxidation of HE and $\hbox {HPr}^{+}$ is known to result in the formation of dimeric products, with two phenanthridine moieties forming a covalent 2,2’ carbon-carbon bond^28,31^. We anticipated that methylation of the amine groups adjacent to the C-2 carbon atoms would result in significant steric hindrance for a dimerization reaction, while still allowing a reaction with $\hbox {O}_{ {2}}^{\bullet -}$, thereby simplifying the profiles of the oxidation products of the probes. In order to test the effect of methylation of the amine groups on the reactivity of the radical cations produced, we compared the rates of $\hbox {HPr}^{\bullet 2+}$ and $\hbox {HE}^{\bullet +}$ decay with the rate of $\hbox {TMeHPr}^{\bullet 2+}$ decay, as well as the rates of the reactions of $\hbox {HPr}^{\bullet 2+}$ and $\hbox {TMeHPr}^{\bullet 2+}$ with $\hbox {O}_{ {2}}^{\bullet -}$. Due to limited water solubility, in the case of TMeHE, we were unable to obtain the sufficient concentration for pulse radiolysis experiments.

**Figure 6:**
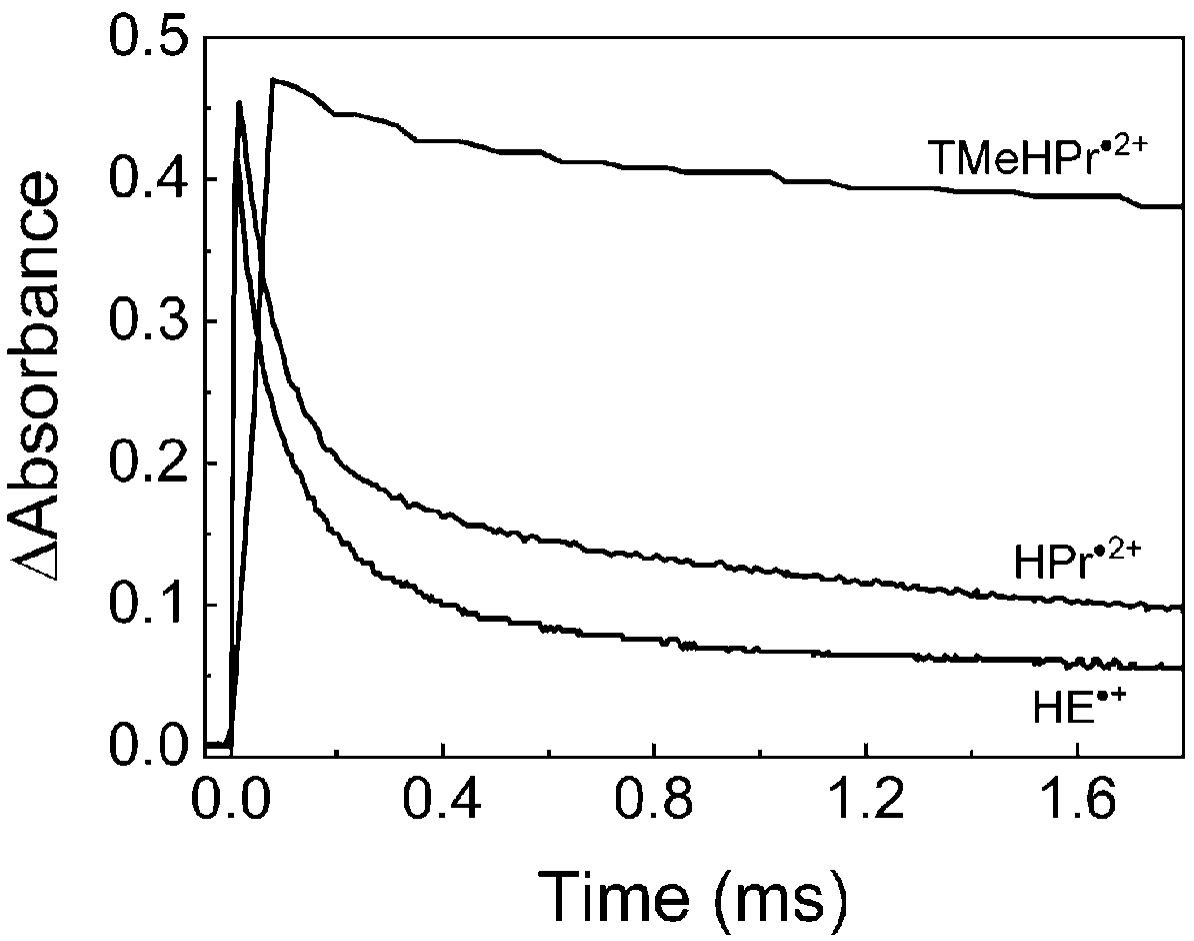
Decay kinetics of radical cations of HE, $\hbox {HPr}^{+}$, and $\hbox {TMeHPr}^{+}$. The decays were monitored at 460 nm for $\hbox {HE}^{\bullet +}$ and $\hbox {HPr}^{\bullet 2+}$, and at 480 nm for $\hbox {TMeHPr}^{\bullet 2+}$. Radical cations were generated by the irradiation of solutions containing 0.1 M of $\hbox {NaN}_{ {3}}$ , 50 mM of phosphate buffer (pH 7.4), and 100 $\upmu$M of HE, or 100 $\upmu$M of $\hbox {HPr}^{+}$, or 60 $\upmu$M of $\hbox {TMeHPr}^{+}$. Radiation doses: 59 Gy for HE, 55 Gy for $\hbox {HPr}^{+}$, and 62 Gy for $\hbox {TMeHPr}^{+}$. Optical path-length: 1 cm.

The kinetic traces for the decay of $\hbox {HE}^{\bullet +}$, $\hbox {HPr}^{\bullet 2+}$ and $\hbox {TMeHPr}^{\bullet 2+}$ are presented in Fig. 6. Using the evaluated molar absorption coefficient of $\hbox {HPr}^{\bullet 2+}$ ($\epsilon _{ {460 \hbox {nm}}} = 3.0\times 10^4$ $\hbox {M}^{-1}\hbox {cm}^{-1}$, Supplementary Table S4) and $\hbox {HE}^{\bullet +}$ ($\epsilon _{ {460 \hbox {nm}}} = 1.7\times 10^4$ $\hbox {M}^{-1}\hbox {cm}^{-1}$, Supplementary Table S4), the rate constants for the observed second-order decay of $\hbox {HPr}^{\bullet 2+}$ and $\hbox {HE}^{\bullet +}$ were estimated to be equal to $(3.3\pm 0.1)\times 10^8$ $\hbox {M}^{-1}\hbox {s}^{-1}$ and $(2.7\pm 0.2)\times 10^8$ $\hbox {M}^{-1}\hbox {s}^{-1}$, respectively (Table S4). The rate constant of $\hbox {TMeHPr}^{\bullet 2+}$ was not determined, because $\hbox {TMeHPr}^{\bullet 2+}$ decay is complex and does not follow simple bimolecular decay kinetics. However, $\hbox {TMeHPr}^{\bullet 2+}$ was significantly more stable than $\hbox {HE}^{\bullet +}$ or $\hbox {HPr}^{\bullet 2+}$ (Fig. 6), demonstrating that N-methylation leads to the increased lifetime of the radical cation.

### Reaction of the radical cation of the probe with $\hbox {O}_{ {2}}^{\bullet -}$

We used $\hbox {TMeHPr}^{+}$ to demonstrate the reaction of the radical cation of the probe with $\hbox {O}_{ {2}}^{\bullet -}$ and to determine its rate constant. We anticipated that the rate of this reaction would not be significantly affected by the N-methylation of amine groups, and thus it should be characteristic for all HE analogues. The much slower decay of $\hbox {TMeHPr}^{\bullet 2+}$ than $\hbox {HPr}^{\bullet 2+}$ and $\hbox {HE}^{\bullet +}$ (Fig. 6) enabled more accurate determination of the rate constant.

**Figure 7:**
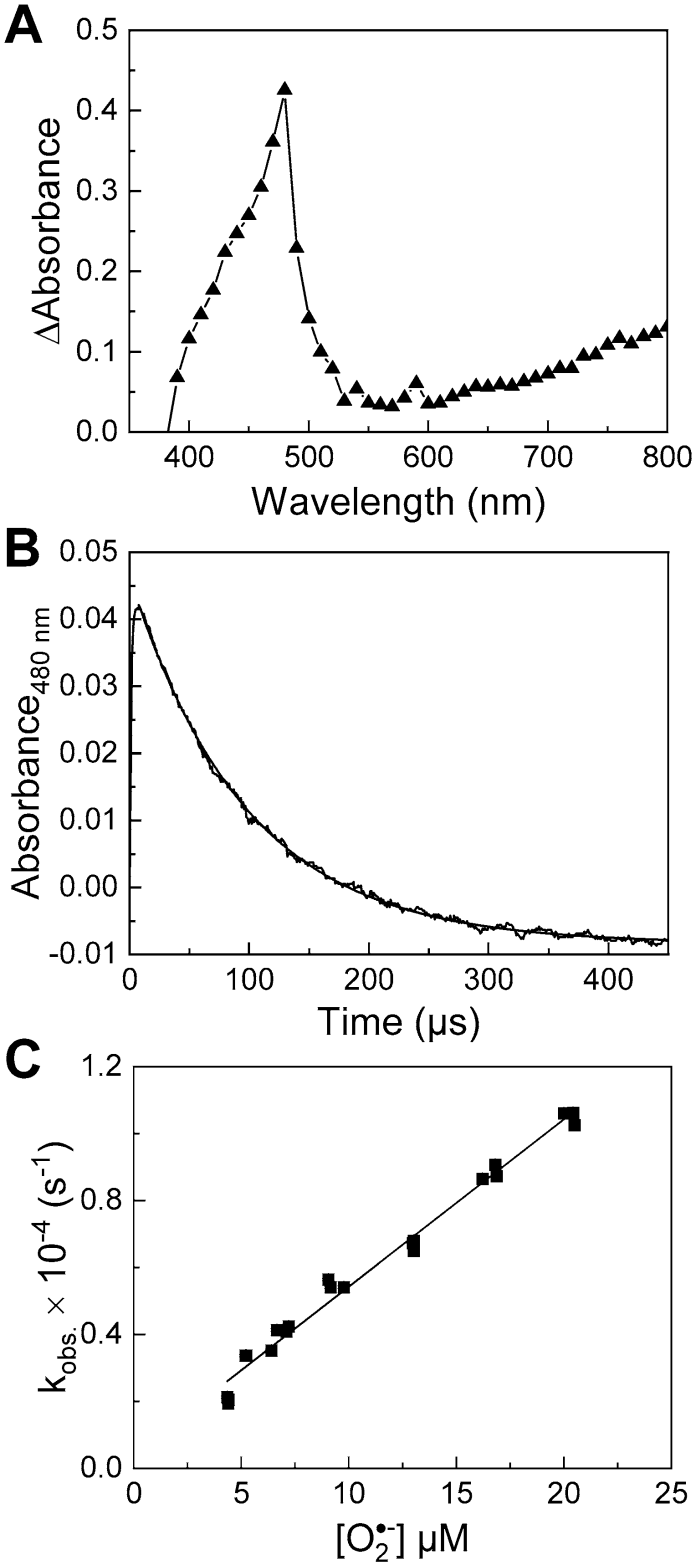
Reaction of $\hbox {TMeHPr}^{\bullet 2+}$ with superoxide. (**A**) Transient absorption spectrum recorded after pulse radiolysis of $\hbox {TMeHPr}^{+}$ (50 $\upmu$M) in an $\hbox {N}_{ {2}}$O-saturated solution containing phosphate buffer (50 mM, pH 7.4) with $\hbox {NaN}_{ {3}}$ (0.1 M). The sample was 1 cm thick, radiation dose: 55 Gy. The spectrum was recorded 9 $\upmu$s after the electron pulse. (**B**) Decay of $\hbox {TMeHPr}^{\bullet 2+}$ due to the reaction with $\hbox {O}_{ {2}}^{\bullet -}$ monitored at 480 nm. The kinetic trace was recorded after pulse radiolysis of an oxygen-saturated aqueous solution containing $\hbox {TMeHPr}^{+}$ (180 $\upmu$M), $\hbox {NaN}_{ {3}}$ (3 mM), formate (50 mM), and phosphate buffer (5 mM, pH 7.4). Radiation dose: 39 Gy. The black lines show the kinetic trace and the pseudo-first order kinetics fit ($\it\hbox {k}_{ {obs}}$) to the experimental data. The concentration of $\hbox {TMeHPr}^{\bullet2+}$ was calculated using the previously determined extinction coefficient of 26,100 $\hbox {M}^{-1}\hbox {cm}^{-1}$. (**C**) Plot of $\it\hbox {k}_{ {obs}}$ against the initial concentration of $\hbox {O}_{ {2}}^{\bullet -}$.

To monitor the reaction of the radical cation $\hbox {TMeHPr}^{\bullet 2+}$ with $\hbox {O}_{ {2}}^{\bullet -}$, both species were generated simultaneously by pulse radiolysis, as described in the SI. One-electron oxidation of $\hbox {TMeHPr}^{+}$ led to the formation of $\hbox {TMeHPr}^{\bullet 2+}$ (Fig. 7A), the lifetime of which was significantly shortened in the presence of $\hbox {O}_{ {2}}^{\bullet -}$ (Fig. 7B). This directly demonstrates for the first time the reaction of the probe radical cation with $\hbox {O}_{ {2}}^{\bullet -}$. The rate constant of the reaction between the generated radical cation and $\hbox {O}_{ {2}}^{\bullet -}$ was determined by monitoring the decay of the radical cation of $\hbox {TMeHPr}^{+}$ at 480 nm with different radiation doses to modulate the concentration of $\hbox {O}_{ {2}}^{\bullet -}$ (see SI). The second-order rate constant determined from the dependence of the observed rate constant on the initial $\hbox {O}_{ {2}}^{\bullet -}$ concentration (Fig. 7C) is equal to $(5.0\pm 0.1)\times 10^8$ $\hbox {M}^{-1}\hbox {s}^{-1}$ (Supplementary Table S4). The second-order rate constant for the reaction of $\hbox {HPr}^{\bullet 2+}$ with $\hbox {O}_{ {2}}^{\bullet -}$ was also determined under analogous experimental conditions, and is equal to $(7.4\pm 0.1)\times 10^8$ $\hbox {M}^{-1}\hbox {s}^{-1}$ (Supplementary Table S4 and Fig. S5). At the concentrations of superoxide generated in these experiments, the self-decay of superoxide *via* dismutation reaction occurs over a significantly longer timescale, and therefore its effect on the observed kinetics can be neglected. We attribute a small, but non-zero intercept observed in Fig. 7C to the self-decay of $\hbox {TMeHPr}^{\bullet 2+}$ in the absence of $\hbox {O}_{ {2}}^{\bullet -}$.

### Characterization of the stable products of one-electron oxidation of TMeHE and $\hbox {TMeHPr}^{+}$

**Figure 8:**
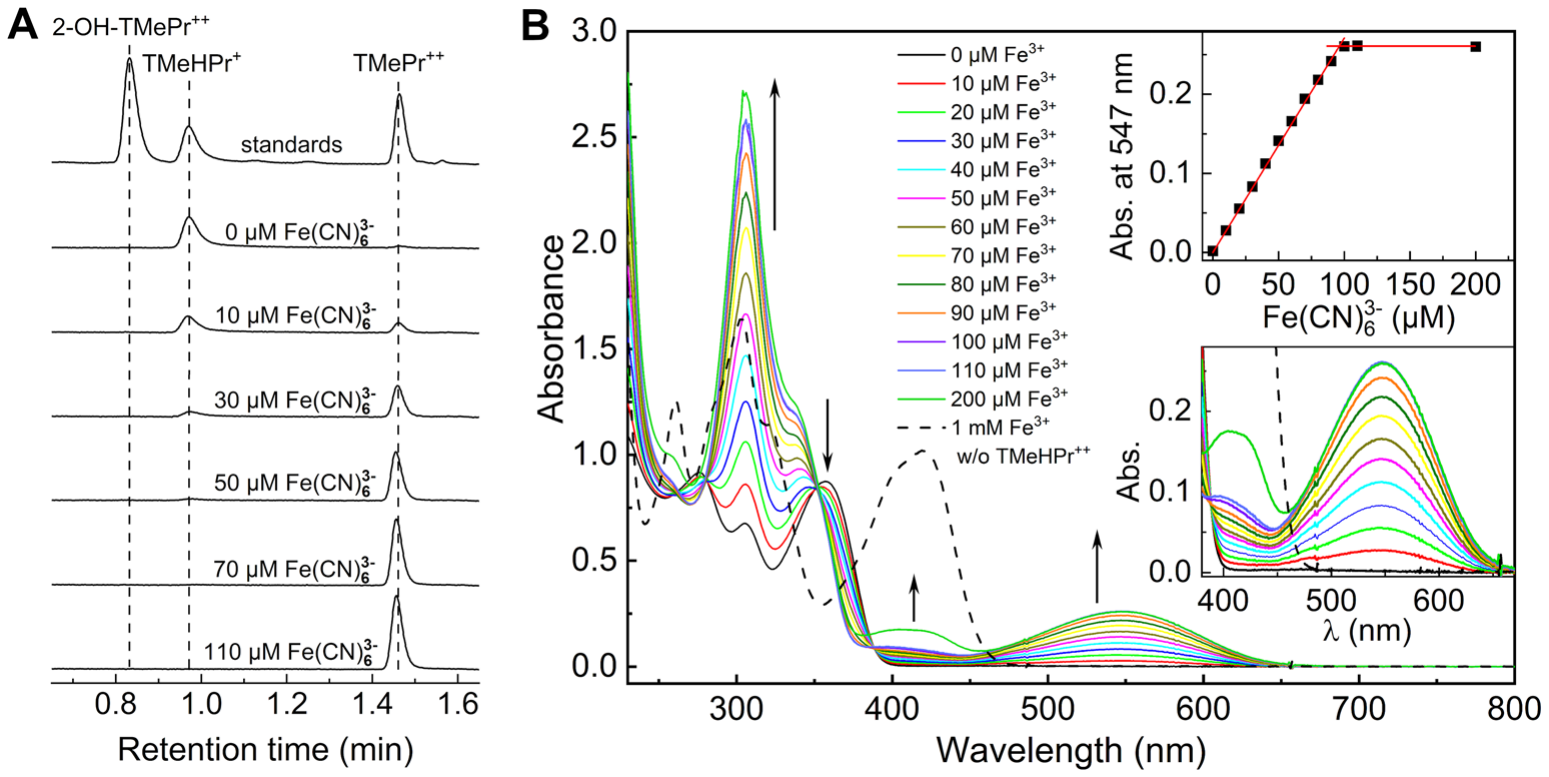
Oxidation of $\hbox {TMeHPr}^{+}$ by the ferricyanide anion. (**A**) UPLC traces obtained for mixtures containing 50 $\upmu$M $\hbox {TMeHPr}^{+}$ and 0–110 $\upmu$M ferricyanide anion. The concentration of the standards was 50 $\upmu$M for $\hbox {TMeHPr}^{+}$, 2-OH-$\hbox {TMePr}^{++}$, and $\hbox {TMePr}^{++}$. The analytical wavelength used for UV–Vis detection: $\lambda = 370\pm 5$ nm. (**B**) UV–Vis absorption spectra of mixtures containing 50 $\upmu$M $\hbox {TMeHPr}^{+}$ and various concentrations of $\hbox {Fe(CN)}_{ {6}}^{3-}$ (0–200 $\upmu$M, represented in the legend as Fe^3+^). Upper inset: dependence of absorbance at 547 nm on $\hbox {Fe(CN)}_{ {6}}^{3-}$ concentration. Lower inset: changes in absorbance between 400 and 670 nm.

We have demonstrated that N-methylation of the probes increases the lifetime of the radical cation (Fig. 6), which may be due to the hindered dimerization process. To determine the products of one-electron oxidation of the methylated probes, we performed titration of a set of $\hbox {TMeHPr}^{+}$ samples, with different concentrations of potassium ferricyanide as a one-electron oxidant. In our previous studies, we found ferricyanide oxidizes HE and $\hbox {HPr}^{+}$ to form $\hbox {E}^{+}$ and $\hbox {Pr}^{++}$, respectively, as well as dimeric products^28,31^. The UPLC traces obtained in the present study for the incubation mixtures containing $\hbox {TMeHPr}^{+}$ and various concentration of $\hbox {Fe(CN)}_{ {6}}^{3-}$ are presented in Fig. 8A. The sole product observed for this reaction is $\hbox {TMePr}^{++}$, which was eluted at 1.45 min (Fig. 8A). These data are in agreement with the UV–Vis measurements conducted under the same experimental conditions (Fig. 8B). The absorption band of $\hbox {TMePr}^{++}$ peaking at 547 nm, which was identified on the basis of the absorption spectrum of its original standard, increases linearly with the addition of $\hbox {Fe(CN)}_{ {6}}^{3-}$ (Fig. 8B, upper inset). The addition of $\hbox {Fe(CN)}_{ {6}}^{3-}$ at a concentration higher than 100 $\upmu$M to the solution containing $\hbox {TMeHPr}^{+}$ at a concentration of 50 $\upmu$M did not cause further increases in $\hbox {TMePr}^{++}$, suggesting a 1:2 stoichiometry. The changes observed for the absorption maximum of $\hbox {K}_{ {3}}\hbox {Fe(CN)}_{ {6}}$ at 420 nm are also shown in Fig. 8B. It can be calculated that the concentration of the remaining $\hbox {K}_{ {3}}\hbox {Fe(CN)}_{ {6}}$ is equal to 100 $\upmu$M for the mixture of 50 $\upmu$M of $\hbox {TMeHPr}^{+}$ and 200 $\upmu$M of $\hbox {K}_{ {3}}\hbox {Fe(CN)}_{ {6}}$ ($\epsilon _{240}$ nm = 1023 $\hbox {M}^{-1}\hbox {cm}^{-1}$)^44^. For the purpose of comparison, the absorption spectrum of a 1 mM solution of $\hbox {K}_{ {3}}\hbox {Fe(CN)}_{ {6}}$ is also shown in Fig. 8B marked by a black dashed line. All presented data indicate 1:2 ($\hbox {HPr}^{+}$:$\hbox {Fe(CN)}_{ {6}}^{3-}$) stoichiometry of the observed reaction and the absence of corresponding dimers formed by the N-methylated analog of $\hbox {HPr}^{+}$. Similar analyses were performed for TMeHE and compared to HE. Using LC/MS we detected $\hbox {TMeE}^{+}$ (the X-Ray structure is presented in SI) as the product, which was eluted at a retention time of 2.44 min (Supplementary Fig. S6). The small peak observed at 2.3 min (Supplementary Fig. S6) was identified as a trimethylethidium cation originating from residual amounts of trimethyl-hydroethidine detected in the TMeHE sample. In the case of HE (Supplementary Fig. S6), oxidation of the probe with the ferricyanide anion led to the formation of several products, the distribution of which was dependent on the concentration of the oxidant. In agreement with our previous results^31^, LC/MS analyses showed that, in addition to $\hbox {E}^{+}$, HE dimers are formed. In the case of TMeHE, no dimers were detected under the same conditions (Supplementary Fig. S6). All the presented data indicate that N-methylation of the probes blocks the dimerization process, leading to the formation of $\hbox {TMeE}^{+}$ or $\hbox {TMePr}^{++}$ as the sole products formed upon one-electron oxidation. In contrast, in the cases of HE and $\hbox {HPr}^{+}$ the dimeric products are formed upon one-electron oxidation, and the stoichiometry of their reaction with the ferricyanide anion is higher due to additional depletion of the oxidant during the formation of the fully oxidized dimers, $\hbox {E}^{+}$-$\hbox {E}^{+}$ and $\hbox {Pr}^{++}$-$\hbox {Pr}^{++}$^28^.

### Oxidation of TMeHE by $\hbox {O}_{ {2}}^{\bullet -}$ in enzymatic and cellular systems

In order to evaluate the impact of methylation of the exocyclic amino groups of HE on its reactivity toward $\hbox {O}_{ {2}}^{\bullet -}$, we incubated TMeHE in a mixture of hypoxanthine and xanthine oxidase as a source of $\hbox {O}_{ {2}}^{\bullet -}$. In the reaction mixture, the chelating agent dtpa was used to avoid the interference with metal ions such as copper and/or iron that can accelerate the dismutation of $\hbox {O}_{ {2}}^{\bullet -}$ or initiate oxidation of the probe *via* Fenton reaction.

The UPLC analyses revealed the build-up of a peak with a retention time of 1.73 min (Supplementary Fig. S7) corresponding to 2-OH-$\hbox {TMeE}^{+}$. This was confirmed by comparison to the retention time of an independently synthesized standard of 2-OH-$\hbox {TMeE}^{+}$ (Supplementary Fig. S7, X-Ray structure available in SI). The influence of SOD and catalase on the formation of 2-OH-$\hbox {TMeE}^{+}$ was also determined (Supplementary Fig. S7). The SOD completely abolished the peak assigned to 2-OH-$\hbox {TMeE}^{+}$. In turn, catalase increased the yield of 2-OH-$\hbox {TMeE}^{+}$ production substantially, which we attribute to an increased steady-state concentration of $\hbox {TMeHE}^{\bullet +}$ ready to react with $\hbox {O}_{ {2}}^{\bullet -}$ and produced by catalase in a peroxidase-like cycle. After three hours of incubation in the absence of XO and SOD, a small amount of 2-OH-$\hbox {TMeE}^{+}$ was formed due to autoxidation of the probe in the presence of oxygen. Autoxidation has also been shown to occur in the case of HE probe but to a lesser extent^32^. The formation of other nonspecific oxidation products, such as the corresponding dimers, was not observed. These results indicate that methylation of the exocyclic amine groups of HE does not prevent the probe from trapping superoxide.

**Figure 9:**
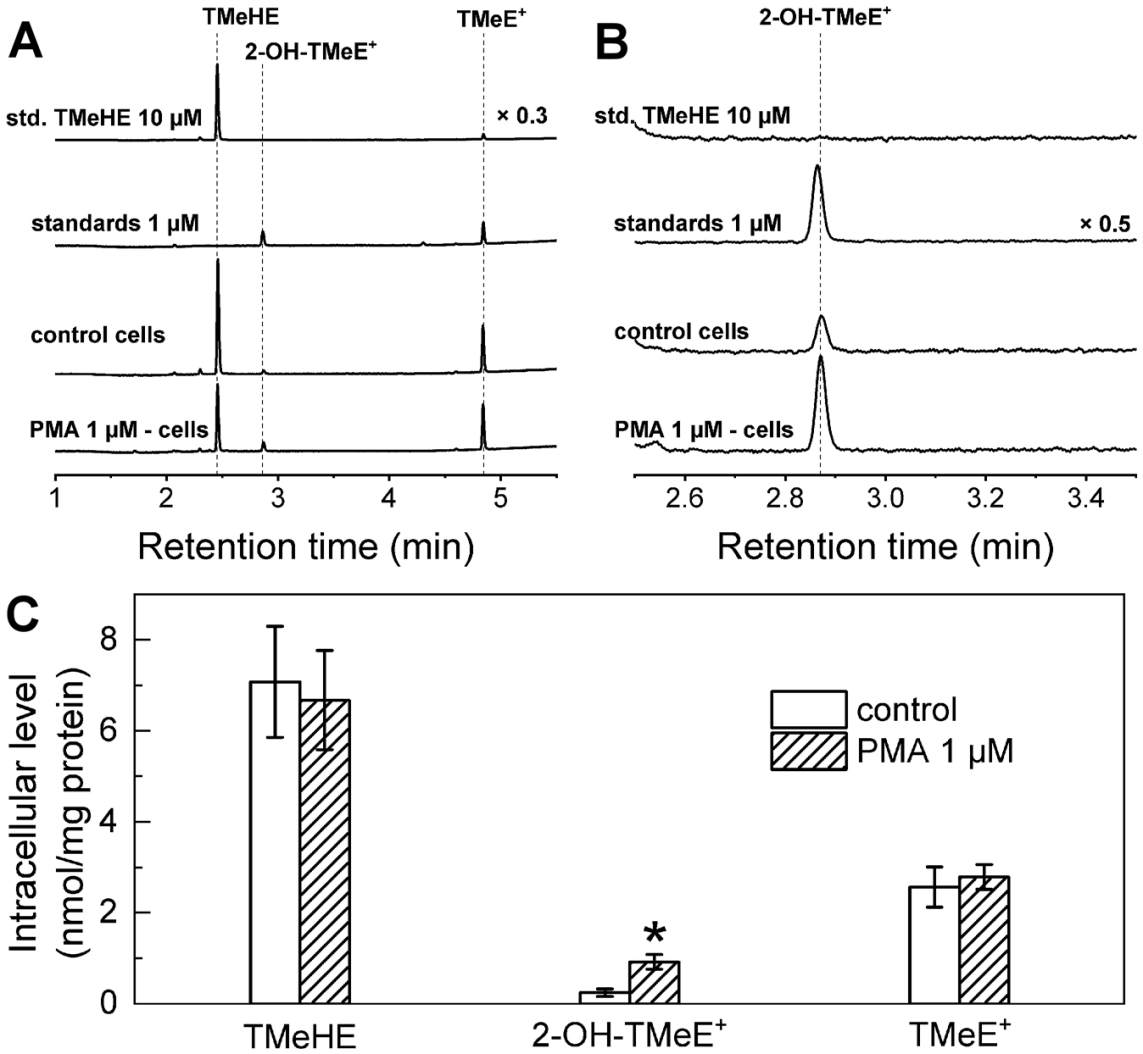
Detection of $\hbox {O}_{ {2}}^{\bullet -}$ in RAW 264.7 cells stimulated PMA (1 $\upmu$M, 1 h), using TMeHE (10 $\upmu$M, 1 h). (**A**) HPLC traces were recorded with the diode array detector set at 370 nm. (**B**) same as (**A**) but with changes in the peak intensities of 2-OH-$\hbox {TMeE}^{+}$ as shown. (**C**) Quantitative data on intracellular levels of **TMeHE** and its oxidation products, as the amount normalized to total cellular protein. T-test vs. control: $*\hbox {p}<0.01$.

To determine whether the TMeHE probe is able to report intracellular superoxide, RAW 264.7 macrophages were stimulated with phorbol 12-myristate 13-acetate (PMA) in the presence of the TMeHE probe. Incubation of macrophages activated to produce $\hbox {O}_{ {2}}^{\bullet -}$ in the presence of TMeHE resulted in the formation of 2-OH-$\hbox {TMeE}^{+}$ (Fig. 9). A significant increase in the amount of 2-OH-$\hbox {TMeE}^{+}$, but not of $\hbox {TMeE}^{+}$, was detected upon stimulation of the cells with PMA (Fig. 9C).

## Discussion

The presented results demonstrate that the peroxynitrite-derived radicals $^{\bullet }\hbox {NO}_{ {2}}$ and $\hbox {CO}_{ {3}}^{\bullet -}$, as well as $\hbox {GS}^{\bullet }$ and $\hbox {CysS}^{\bullet }$, are capable of oxidizing HE and $\hbox {HPr}^{+}$ probes *via* a single electron transfer pathway. The values of the determined rate constants are shown in Supplementary Table S1. Nonetheless, in the presence of glutathione and proteins at millimolar concentrations, the direct reaction of $^{\bullet }\hbox {NO}_{ {2}}$ and $\hbox {CO}_{ {3}}^{\bullet -}$ with HE or $\hbox {HPr}^{+}$ seems rather unlikely under *in vivo* conditions. The alternate scenario is that the protein-centered radicals induced by $^{\bullet }\hbox {NO}_{ {2}}$, $\hbox {CO}_{ {3}}^{\bullet -}$, and other strong one-electron oxidants may oxidize the probe and increase the yields of $\hbox {2-OH-E}^{+}$ observed *in vivo*. Recently, it also has been proposed that nitrosoperoxocarbonate, $\hbox {ONOOCO}_{ {2}}^{-}$, may be more stable than initially assumed and may also act as a strong one-electron oxidant^45^. Prütz et al have shown that upon initial protein oxidation, intramolecular electron transfer leads to the formation of tyrosyl radicals ($\hbox {E}^{o,}$($\hbox {TyrO}^{\bullet }$, $\hbox {H}^{+}$/TyrOH) = 0.96 V)^46,47^. In turn, intramolecular electron transfer from the tyrosyl radical to the cysteine residue leads to thiyl radicals ($\hbox {CysS}^{\bullet }$)^48^. The redox potentials of both couples ($\hbox {TyrO}^{\bullet }$, $\hbox {H}^{+}$/TyrOH and $\hbox {CysS}^{\bullet }$, $\hbox {H}^{+}$/CysSH) are similar and both types of radicals may be involved in HE oxidation *in vivo*^48^. Because the radical cation of HE and its derivatives react rapidly with $\hbox {O}_{ {2}}^{\bullet -}$ to form hydroxylated products, the presence of such oxidants may increase the yield of the superoxide-specific product, even if the $\hbox {O}_{ {2}}^{\bullet -}$ level *per se* is not increased. We have previously reported similar observations regarding the effect of peroxidase on the yield of $\hbox {2-OH-E}^{+}$^33^.

We also estimated the second-order rate constant for the reaction of $\hbox {HO}_{ {2}}^{\bullet }$ with HE as $\sim 1\times 10^7$ $\hbox {M}^{-1}\hbox {s}^{-1}$ (Fig. 4). Using this value and taking into account the $\hbox {p}{K}_{ {a}}$ value of $\hbox {HO}_{ {2}}^{\bullet }$ (4.8)^49^ the apparent second-order rate constant of the reaction between $\hbox {O}_{ {2}}^{\bullet -}$ and HE at pH 7.4 was found to be equal to $2.5\times 10^4$ $\hbox {M}^{-1}\hbox {s}^{-1}$. This apparent rate constant was calculated assuming that, in the system producing $\hbox {O}_{ {2}}^{\bullet -}$, $\hbox {HO}_{ {2}}^{\bullet }$ is the sole species oxidizing HE. This value is in reasonable agreement with the value determined from competition kinetics with SOD, $k=(6.2\pm 0.8)\times 10^3$ $\hbox {M}^{-1}\hbox {s}^{-1}$^28^, and is one order of magnitude higher than the value obtained from fluorescence measurements coupled to computational modeling, $k = (2.17\pm 0.06)\times 10^3$ $\hbox {M}^{-1}\hbox {s}^{-1}$^50^. Additionally, this value is in a good agreement with those obtained for $\hbox {HPr}^{+}$ and MitoHE, $k = (1.19\pm 0.05)\times 10^4$ $\hbox {M}^{-1}\hbox {s}^{-1}$ and $k = (1.6\pm 0.8)\times 10^4$ $\hbox {M}^{-1}\hbox {s}^{-1}$, respectively^28^. Taking into account that the chemical reactivities of HE, Mito-HE, and $\hbox {HPr}^{+}$ are very similar, this suggests that $\hbox {HO}_{ {2}}^{\bullet }$ is the actual species oxidizing HE and HE derivatives in the $\hbox {O}_{ {2}}^{\bullet -}$ producing system. Further studies are required to explore the possibility of the involement of proton-coupled electron transfer (PCET) in the mechanism of oxidation of HE-based probes by $\hbox {O}_{ {2}}^{\bullet -}$^51,52^.

Experiments under cryogenic conditions enable unambiguous assignment of the observed transient species to the radical cations of $\hbox {HPr}^{+}$ and HE, and exclude the contribution of aromatic aminyl radicals ($\hbox {HPr}^{+}(^{\bullet }\hbox {NH})$ or $\hbox {HE}(^{\bullet }\hbox {NH})$) to the observed absorption spectra (Fig. 5). The good agreement between the experimental data obtained from cryogenic measurements for radical cations of HE, $\hbox {HPr}^{+}$, MeE, and TMeHE and the results obtained from TD-DFT calculations also confirms the proper assignment of the primary species observed in the time-resolved pulse radiolytic studies (Fig. 3). Moreover, the characteristic electronic absorption spectra obtained from cryogenic measurements for the radical cations of HE and $\hbox {HPr}^{+}$, as well as for MeE and TMeHE (Fig. 5), are similar to the electronic absorption spectrum obtained for the benzidine radical cation (Supplementary Fig. S8). The intense and structured transient absorption bands at 450 nm, 800 nm, and 900 nm attributed to the benzidine radical cation are in agreement with literature data^53,54^. The $\hbox {p}{K}_{ {a}}$ of the radical cation of benzidine is 10.9, which is four units higher than that of the aniline radical cation^55^. The lack of differences between the spectra of $\hbox {HPr}^{\bullet 2+}$ and $\hbox {HE}^{\bullet +}$ observed at pH 10.5 and pH 7.4 suggests an even higher $\hbox {p}{K}_{ {a}}$ value for these radical cations (Fig. 3)^34^.

According to the mechanism of $\hbox {2-OH-E}^{+}$ formation, the affinity of nucleophilic $\hbox {O}_{ {2}}^{\bullet -}$ to the radical cation should depend strongly on the spin density at the C-2 carbon atom and to a lesser extent, due to the small size of the $\hbox {O}_{ {2}}^{\bullet -}$ molecule, on the steric hindrance of the $\alpha$-amine group. Quantum mechanical calculations showed that the highest spin density was located at the C-2 and C-12 carbon atoms of the $\hbox {HPr}^{+}$, HE, MeE, and TMeHE radical cations (Supplementary Fig. S4 and Table S3). This supports the hypothesis that $\hbox {O}_{ {2}}^{\bullet -}$ reacts with the radical cation of the hydroethidine-based probe, through a direct attack on the C-2 carbon atom, yielding the 2-hydroxylated cationic product.

Using close structural analogues of HE, namely $\hbox {HPr}^{+}$ and $\hbox {TMeHPr}^{+}$, we demonstrated that the radical cations of both probes react with $\hbox {O}_{ {2}}^{\bullet -}$ with high reaction rates (Fig. 7, Supplementary Figure S5 and Table S4). To our knowledge, this is the first direct observation of this reaction, and it explains the recently reported incorporation of an oxygen atom from $^{18}\hbox {O}_{ {2}}^{\bullet -}$ in the $\hbox {2-OH-E}^{+}$ product^56^.

**Figure 10:**
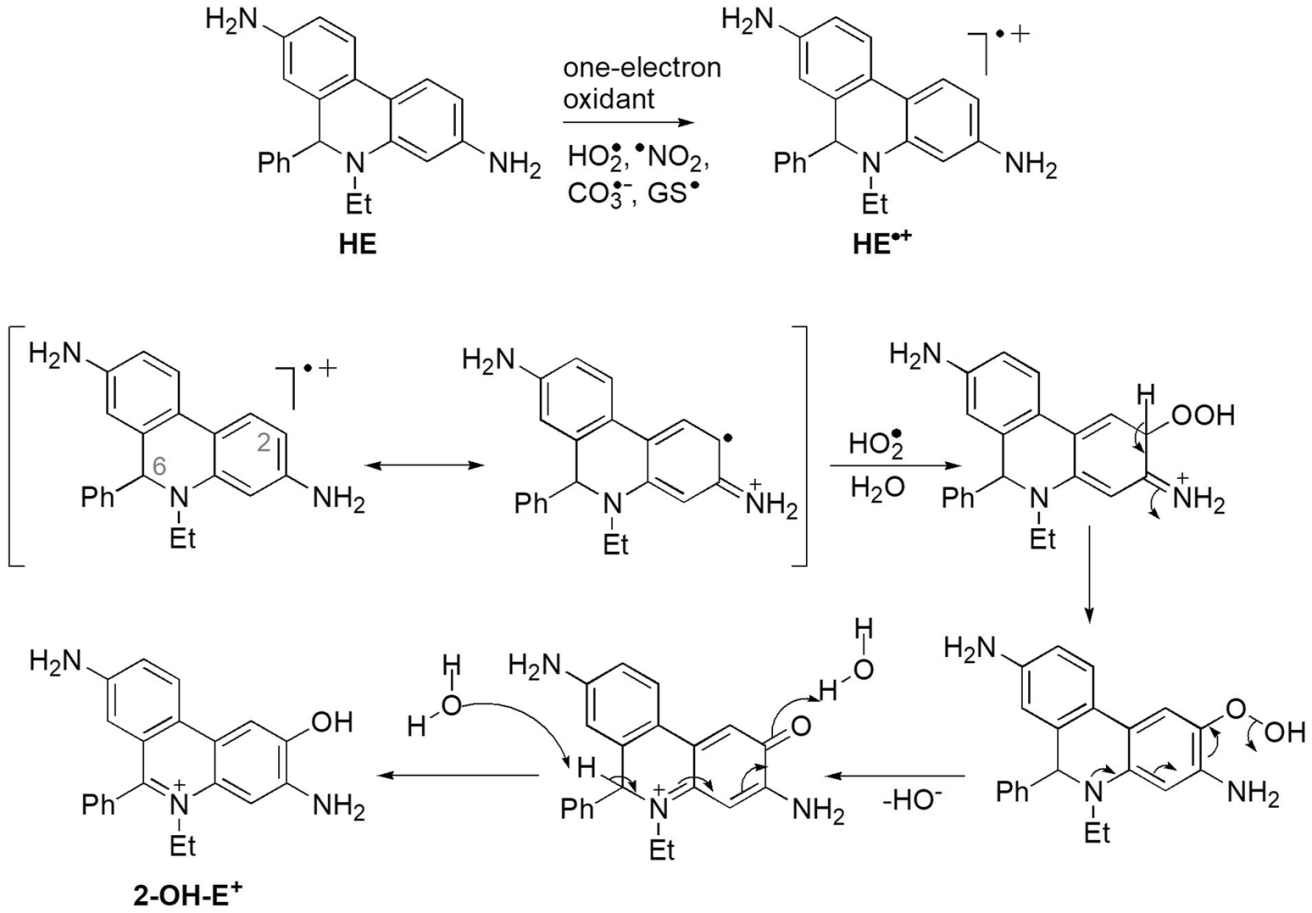
Mechanism of HE oxidation to $\hbox {2-OH-E}^{+}$ in the presence of one-electron oxidants and $\hbox {O}_{ {2}}^{\bullet -}$.

Our results led us to postulate the following mechanism for the oxidation of HE to $\hbox {2-OH-E}^{+}$ that is relevant to its other derivatives (Fig. 10). In the first step, HE is oxidized to the radical cation by one-electron oxidants like $^{\bullet }\hbox {NO}_{ {2}}$, $\hbox {CO}_{ {3}}^{\bullet -}$, $\hbox {GS}^{\bullet }$ or $\hbox {HO}_{ {2}}^{\bullet }$. Then, the recombination of $\hbox {HE}^{\bullet +}$ with $\hbox {O}_{ {2}}^{\bullet -}$ forms a hydroperoxide derivative followed by elimination of hydroxide to form a quinone derivative. The final step of the reaction is the transformation of the quinone derivative to $\hbox {2-OH-E}^{+}$ (Fig. 10).

In the absence of superoxide, the radical cation of HE and its derivatives decay by dimerization and/or by disproportionation reaction (Fig. 2). Interestingly, methylation of the exocyclic amine groups blocks dimerization of the radical cation. Probably, the bulky $\alpha$-dimethylamine group present in the structures of TMeHE and $\hbox {TMeHPr}^{+}$ causes steric hindrance around the C-2 carbon atom, the site of new C-C bond formation by two dimerizing radicals^28,31^, and impedes dimer formation for TMeHE and $\hbox {TMeHPr}^{+}$.

We also examined the reactivity of TMeHE toward superoxide (Supplementary Fig. S7). In the presence of $\hbox {O}_{ {2}}^{\bullet -}$, TMeHE was found to form 2-OH-$\hbox {TMeE}^{+}$. In combination with the fact that one-electron oxidation of TMeHE is not accompanied by the formation of any dimeric products (Supplementary Fig. S6), this finding encouraged us to test TMeHE in the cellular system. Incubation of TMeHE with cells activated to produce $\hbox {O}_{ {2}}^{\bullet -}$ resulted in the formation of 2-OH-$\hbox {TMeE}^{+}$ (Fig. 9). This result is in agreement with the results obtained for the HE probe reported previously^29,30,34,57–60^. Overall, TMeHE can be utilized for the detection of cellular $\hbox {O}_{ {2}}^{\bullet -}$ and, taking into account its less complicated oxidation products profile, TMeHE seems to be an improved candidate for this specific purpose. Furthermore, the unimpeded reactivity of the N-methylated analogs towards superoxide open the way for future modifications of the probes using amine groups as the site of derivatization.

## Conclusions

This study has demonstrated that hydroethidine and related probes are rapidly oxidized by an array of biologically relevant oxidants, including $\hbox {HO}^{\bullet }$, $^{\bullet }\hbox {NO}_{ {2}}$, $\hbox {CO}_{ {3}}^{\bullet -}$, $\hbox {GS}^{\bullet }$, and $\hbox {CysS}^{\bullet }$. The initial product of this reaction is the radical cation of the probe. Analysis of the kinetics of oxidation of HE by different peroxyl radicals and by superoxide, points to $\hbox {HO}_{ {2}}^{\bullet }$ as the actual oxidant of HE in the presence of $\hbox {O}_{ {2}}^{\bullet -}$. We have also demonstrated that the radical cation formed during oxidation of the probe by $\hbox {HO}_{ {2}}^{\bullet }$ or other oxidants, reacts rapidly with $\hbox {O}_{ {2}}^{\bullet -}$ to produce the $\hbox {O}_{ {2}}^{\bullet -}$-specific hydroxylated product. Methylation of the amine groups of HE was found to block the dimerization of the probe simplifying product distribution, while not significantly affecting the reaction with $\hbox {O}_{ {2}}^{\bullet -}$. This opens a new route for the derivatization of the HE probe and the generation of new analogs for improved performance in detecting $\hbox {O}_{ {2}}^{\bullet -}$ in biological systems.

## Methods

All synthetic procedures including the synthesis of $\hbox {HPr}^{+}$, TMeHE, $\hbox {TMeE}^{+}$, 2-OH-$\hbox {TMeE}^{+}$, $\hbox {TMeHPr}^{+}$, $\hbox {TMePr}^{++}$, 2-OH-$\hbox {TMePr}^{++}$, and MeE, together with the MS and NMR data, are described in the SI. Pulse radiolysis experiments were performed with a 6-MeV linear accelerator at the Institute of Applied Radiation Chemistry (Lodz, Poland). A detailed description of the experiments is given in the SI.

### Kinetic simulations

The kinetic simulations were carried out using Kintecus 4.80^61^. The list of the chemical reactions and values of the corresponding rate constants are shown in SI.

### Cryogenic measurements

Ionic liquid-based cryogenic glasses were prepared by immersing a solution of the appropriate compound in liquid nitrogen. The thickness of the glassy samples was below 1 mm. The samples were placed in a temperature-controlled, liquid nitrogen-cooled cryostat (OptiStat DN, Oxford Instruments, United Kingdom), then irradiated with 4 $\upmu$s electron pulses from a linear accelerator. The UV–Vis absorption spectra were recorded with a Cary 5 (Varian Medical Systems, United States) spectrophotometer. Radiolysis of the glassy sample of $\hbox {BMIM}^{+}\hbox {PF}_{ {6}}^{-}$ results in the generation of electrons and oxidizing holes represented by the 1-butyl-3-methylimidazolium ($\hbox {BMIM}^{\bullet 2+}$) radical dication and hexafluorophosphate radical ($\hbox {PF}_{ {6}}^{\bullet }$). Solvated electrons are captured by $\hbox {BMIM}^{+}$ cations yielding $\hbox {BMIM}^{\bullet }$ radicals, which prevent geminate recombination of the primary species^62,63^. In the presence of halogenated compounds like dichloromethane, instead of $\hbox {BMIM}^{\bullet }$ formation, the electrons are scavenged through dissociative electron attachment^64,65^. $\hbox {BMIM}^{\bullet 2+}$ and $\hbox {PF}_{ {6}}^{\bullet }$ are strong one-electron oxidants; they accept electrons from the solute molecules of lower ionization potential (e.g., $\hbox {HPr}^{+}$) producing the corresponding one-electron oxidized species (e.g., $\hbox {HPr}^{\bullet 2+}$).

### QM calculations

All calculations were performed using the Gaussian G09W suite of programs^66^. The gas-phase geometry of the studied radical cations was optimized using the unrestricted B3LYP density functional method^67,68^. The 6-311+G(d,p) basis set was used for geometry optimizations and energy calculations. No imaginary frequencies were observed for the converged structures of the studied radical cations. Mulliken atomic spin densities were obtained from the gas-phase UB3LYP/6-31+G(d, p) geometries. Spin density maps were generated from formatted checkpoints using the Cubegen utility provided with Gaussian. Spin density maps were formed by the superposition of the spin density surface on the electron density surface. Excited-state calculations were carried out on the basis of time-dependent response theory, along with unrestricted B3LYP density functional theory (the so-called time-dependent density functional theory [TD-DFT] method)^69^. All calculations were carried out with the default convergence criteria.

### UPLC/UV–Vis/MS analyses

The ultra-performance liquid chromatography (UPLC) system (UPLC Acquity, Waters Ltd., United States) equipped with a photodiode array detector for UV–Vis absorption measurements and LCT Premier XE (Water Micromass, United States) mass spectrometry detector was used to investigate the products of the reaction of TMeHE and $\hbox {TMeHPr}^{+}$ with the oxidants. Separation of TMeHE was accomplished on a Waters Ltd. UPLC column (Acquity UPLC BEH C18, 1.7 $\upmu$m, $50\times 2.1$ mm), kept at 40 $^{\circ }\hbox {C}$ and equilibrated with a mobile phase consisting of water/MeCN, 70:30 (v/v), at a flow rate of 0.3 ml/min for at least 0.5 min. Both the organic and water phases contained 0.1% (v/v) trifluoroacetic acid (TFA). Next, the concentration of organic phase was increased linearly up to 70% (v/v) over 1.55 min. It was then raised rapidly up to 100% (v/v) over the next 0.1 min and kept at this level for 0.65 min. The analytes, TMeHE, 2-OH-$\hbox {TMeE}^{+}$, and $\hbox {TMeE}^{+}$, were eluted at retention times of 1.53 min, 1.72 min, and 2.44 min, respectively, and detected by monitoring the absorption at $370\pm 10$ nm.

Separation of $\hbox {TMeHPr}^{+}$ was performed using an Acquity UPLC CSH Phenyl-Hexyl column (1.7 $\upmu$m, $50\times 2.1$ mm) equilibrated with water/methanol mobile phase (60:40 v/v) containing 0.1% vol. of TFA, at a flow rate of 0.3 ml/min. The $\hbox {TMeHPr}^{+}$, 2-OH-$\hbox {TMePr}^{++}$, and $\hbox {TMePr}^{++}$ were eluted at retention times of 0.83 min, 0.97 min, and 1.46 min, respectively, using the following gradient method: The initial concentration of the organic phase was applied for 0.5 min; over next 1.5 min concentration of organic phase was increased linearly to 100%; the column was then rinsed with 100% (v/v) organic phase for another 1 min.

The products of HE oxidation by ferricyanide anion were separated using an Acquity UPLC BEH C18 column (1.7 $\upmu$m, $50\times 2.1$ mm) and the gradient method, as described elsewhere^32^. The identity of the analytes was confirmed by mass spectrometry analysis using the m/z ratio obtained from the experiment and calculated based on the molecular structure, as shown in Supplementary Tables S1 and S2. The injection volumes and temperatures for both the samples and the standard solutions were 2 $\upmu$l and 23 $^{\circ }\hbox {C}$ in case of the TMeHE and HE probes, and 0.5 $\upmu$l and 20 $^{\circ }\hbox {C}$ for $\hbox {TMeHPr}^{+}$. Data acquisition was performed using MassLynx 4.1 data software (Waters Ltd., United States).

### Cell culture experiments

RAW 264.7 cells were obtained over the last five years, stored in liquid nitrogen, and used within 20 passages after thawing. The cells were grown at 37 $^\circ \hbox {C}$ in 5% $\hbox {CO}_{ {2}}$. The cells were maintained in DMEM (CAT#11965, Invitrogen, San Diego, CA) containing 10% (v/v) fetal bovine serum, penicillin (100 U/ml) and streptomycin (0.1 mg/ml).

The RAW 264.7 cells were cultured as described previously^28^. The cells were incubated with phorbol 12-myristate 13-acetate (PMA, 1 $\upmu$M) and TMeHE (10 $\upmu$M) for 1 hour at 37 $^\circ \hbox {C}$ in 5% $\hbox {CO}_{ {2}}$. After incubation, 100 $\upmu$L of the medium was transferred to an Eppendorf tube and frozen in liquid nitrogen. The rest of the medium was discarded and the cells were washed twice using ice-cold Dulbecco’s phosphate buffered saline (DPBS). The washed cells were scraped in 1 mL of DPBS, transferred to an Eppendorf tube and centrifuged for 1 min. Then, the supernatant was aspirated and the cell pellet was frozen in liquid nitrogen.

### Processing of cell pellets

Frozen cell pellets were placed on ice and syringe lysed, using 10 strokes through a 28 ga needle, in 200 $\upmu$l of 0.1% vol. Triton X-100 in ice-cold phosphate buffered saline containing 1 $\upmu$M of 3,8-diamino-6-phenylphenanthridine (DAPP) as an internal standard. The probes and their oxidation products were then extracted by adding 100 $\upmu$l of the resulting mixture to 100 $\upmu$l of 0.1% vol. formic acid in MeCN. The samples were incubated on ice for 1 h, and then centrifuged for 30 min at 20,000*g* at 4 $^{\circ }\hbox {C}$. A volume of 100 $\upmu$L of the supernatant was then transferred to a fresh tube containing 100 $\upmu$l of 0.1% vol. formic acid in water. This solution was centrifuged for an additional 15 min at 20,000*g* at 4 $^{\circ }\hbox {C}$. A volume of 150 $\upmu$L of the resulting supernatant was then transferred to HPLC vials for analysis^59^.

### HPLC analysis of cell extracts

HPLC analyses were performed by adopting the previous method^70^. The samples were separated using an Agilent 1100 system (North Billerica, MA) equipped with absorption and fluorescence detectors. During the HPLC analyses, samples were stored at 4 $^{\circ }\hbox {C}$ and the injection volume was 50 $\upmu$l. For the separation of analytes, a reverse phase column (Phenomenex, Kinetex C18, 100 mm $\times$ 4.6 mm, 2.6 $\upmu$m) was used. Prior to injection, the column was equilibrated with a mobile phase consisting of 20% MeCN and 80% water (v/v). The organic and aqueous mobile phases contained 0.1% (v/v) TFA.

The TMeHE and its oxidation products were separated using the gradient method. The fraction of MeCN was increased during the analysis linearly from 20 to 40% over 1 min, then, from 40 to 49% over 2 min and from 49 to 100% over 2 min. An absorption detector was used to measure DAPP (at 290 nm; retention time: 2.0 min), 2-OH-$\hbox {TMeE}^{+}$ (at 290 nm; retention time: 2.9 min), and TMeHE (at 370 nm; retention time: 2.4 min). TMeHE, 2-OH-$\hbox {TMeE}^{+}$, and $\hbox {TMeE}^{+}$ were also monitored fluorometrically using the following excitation and emission wavelengths: 358 nm/400 nm for TMeHE, 490 nm/608 nm for 2-OH-$\hbox {TMeE}^{+}$, and 555 nm/625 nm for $\hbox {TMeE}^{+}$ (retention time: 4.8 min).

## Supplementary information

Supplementary material 1.
