# Supplementary material for: Oxidation of ethidium-based probes by biological radicals: mechanism, kinetics and implications for the detection of superoxide
Source: Sci Rep. 2020 Oct 29;10:18626. doi: 10.1038/s41598-020-75373-2 (PMC7596101; doi:10.1038/s41598-020-75373-2)
Supplement: Supplementary file 1 — Supplementary material 1. [file 41598_2020_75373_MOESM1_ESM.pdf]

Oxidation of ethidium-based probes by biological radicals – mechanism, kinetics and implications for the detection of superoxide

Radosław Michalski,[1,*] David Thiebaut,[2] Bartosz Michałowski,[1] Mehmet M. Ayhan,[2,3] Micael Hardy,[2] Olivier Ouari,[2] Michał Rostkowski,[1] Renata Smulik-Izydorczyk,[1] Angelika Artelska,[1] Andrzej Marcinek,[1] Jacek Zielonka,[4] Balaraman Kalyanaraman,[4] and Adam Sikora[1,*]

[1]*Institute of Applied Radiation Chemistry, Lodz University of Technology, Zeromskiego 116, 90-924 Lodz, Poland*

[2]*Aix Marseille Univ, CNRS, ICR, UMR 7273, 13013 Marseille, France*

[3]*Department of Chemistry, Gebze Technical University, P.K.:141, 41400 Gebze, Kocaeli, Turkey*

[4]*Department of Biophysics and Free Radical Research Center, Medical College of Wisconsin, 8701 Watertown Plank Road, Milwaukee, WI 53226, United States*

SUPPLEMENTARY INFORMATION

Table of Contents

Supplementary Figure S1.. 6

Supplementary Figure S2.. 7

Supplementary Figure S3.. 8

Supplementary Figure S4.. 9

Supplementary Figure S5.. 10

Supplementary Figure S6.. 11

Supplementary Figure S7.. 12

Supplementary Figure S8.. 13

Supplementary Table S1.. 14

Supplementary Table S2.. 14

Supplementary Table S3.. 15

Supplementary Table S4.. 16

Supplementary Table S5.. 17

Supplementary Table S6.. 18

Synthesis of hydroethidine and hydropropidine derivatives.. 19

Materials.. 21

Synthetic procedures.. 21

Synthesis of 3,8-tetramethylamino-5-diethyl-6-phenylphenantridinium (**TMeE$^+$**)................ 22

Synthesis of 3,8-tetramethylamino-5-diethyl-6-phenylphenantridine (**TMeHE**).................. 23

Synthesis of 3,8-tetramethylamino-5-diethyl-2-hydroxy-6-phenylphenantridium (**2-OH-TMeE$^+$**).. 23

Synthesis of 3,8-tetramethylamino-6-phenylphenanthridine (**1**).. 24

Synthesis of 3-iodopropyl-trifluoromethanesulfonate... 24

Synthesis of 3,8-tetramethylamino-5-(3-iodopropyl)-6-phenylphenanthridinium (**2**).......... 25

Synthesis of 3,8-tetramethylamino-5-[3-(diethylmethylammonio)propyl]-6-phenylphenantridinium (**TMePr^{++}**).. 25

Synthesis of 3,8-tetramethylamino-5-[3-(diethylmethylammonio)propyl]-6-phenylphenantridine (**TMeHPr^{+}**).. 26

Synthesis of 3,8-bis-benzyloxycarbonylamino-6-phenylphenanthridine (**3**)......................... 27

Synthesis of 3,8-bis-benzyloxycarbonylamino-5-(3-iodopropyl)-6-phenylphenanthridinium (**4**).. 28

Synthesis of 3,8-bis-benzyloxycarbonylamino-5-[3-(diethylmethylammonio)propyl]-6-phenylphenanthridinium (**5**).. 28

Synthesis of 3,8-diamino-5-[3-(diethylmethylammonio)propyl]-6-phenylphenantridine (**HPr^{+}**).. 29

Synthesis of 3,8-tetrametylamino-5-[3-(diethylmethylammonio)propyl]-6-phenylphenantridinium (**TMePr^{++}**).. 29

Synthesis of 3,8-tetramethylamino-5-[3-(diethylmethylammonio)propyl]-2-hydroxy-6-phenylphenantridine (**2-OH-TMePr^{++}**).. 30

Synthesis of methylethidine (MeE).. 30

Pulse radiolysis experiments.. 31

Generation of azidyl radical (**$N_3^{\bullet}$**).. 31

Generation of carbonate radical anion (**$CO_3^{\bullet-}$**).. 32

Generation of nitrogen dioxide radical (**$^{\bullet}NO_2$**).. 33

Generation of thiyl radicals (**$GS^{\bullet}$** or **$CysS^{\bullet}$**).. 33

Generation of chloromethylperoxyl radicals.. 34

Generation of superoxide radical anion (**$O_2^{\bullet-}$**) and its reaction with **$HPr^{\bullet 2+}$** and **$TMeHPr^{\bullet 2+}$**. 35

Kinetic simulations.. 36

^{1}H NMR ($CDCl_3$) of compound **$TMeE^+$**.. 38

^{13}C NMR ($CDCl_3$) of compound **$TMeE^+$**... 38

Geometry of **$TMeE^+$** from X-ray diffraction analysis 39

^{1}H NMR ($CDCl_3$) of compound **TMeHE**.. 40

^{13}C NMR ($CDCl_3$) of compound **TMeHE**... 40

^{1}H NMR ($CDCl_3$) of **2-OH-$TMeE^+$**.. 41

^{13}C NMR ($CDCl_3$) of compound **2-OH-$TMeE^+$**... 41

Geometry of **2-OH-$TMeE^+$** from X-ray diffraction analysis... 42

^{1}H NMR (CD_3OD) of compound **1**... 43

^{13}C NMR (CD_3OD) of compound **1**.. 43

HPLC and ESI-MS analysis of compound **1**.. 44

^{1}H NMR ($CDCl_3$) of 3-iodopropyl trifluoromethanesulfonate... 45

^{1}H NMR ($CDCl_3$) of compound **2**... 46

^{13}C NMR ($CDCl_3$) of compound **2**.. 46

HPLC analysis of compound **2**.. 47

^{1}H NMR (CD_3OD) of **$TMePr^{++}$**.. 48

^{13}C NMR (CD_3OD) of **$TMePr^{++}$**... 48

HPLC analysis of compound **$TMePr^{++}$** (λ= 290 nm).. 49

^{1}H NMR (D_2O) of **$TMeHPr^+$**.. 49

^{13}C NMR (D_2O) of **$TMeHPr^+$**... 50

HSQC (D_2O) of **TMeHPr⁺**.. 51

HMBC (D_2O) of **TMeHPr⁺**.. 52

HPLC analysis of compound **TMeHPr⁺** (λ= 290 nm).. 52

^{1}H NMR (CD_3OD) of **2-OH-TMePr⁺⁺**... 53

^{13}C NMR (CD_3OD) of **2-OH-TMePr⁺⁺**... 53

HSQC (CD_3OD) of **2-OH-TMePr⁺⁺**.. 54

HMBC (CD_3OD) of **2-OH-TMePr⁺⁺**.. 55

^{1}H NMR (DMSO d_6) of compound **3**.. 56

^{13}C NMR (DMSO d_6) of compound **3**.. 56

HPLC and ESI-MS of compound **3**.. 57

^{1}H NMR (CD_3OD) of compound **4**... 58

HPLC and ESI-MS of compound **4**.. 59

^{1}H NMR (CD_3OD) of compound **5**... 60

^{13}C NMR ($CDCl_3$) of compound **5**... 60

HPLC and ESI-MS of compound **5**.. 61

Abbreviations.. 62

References.. 64

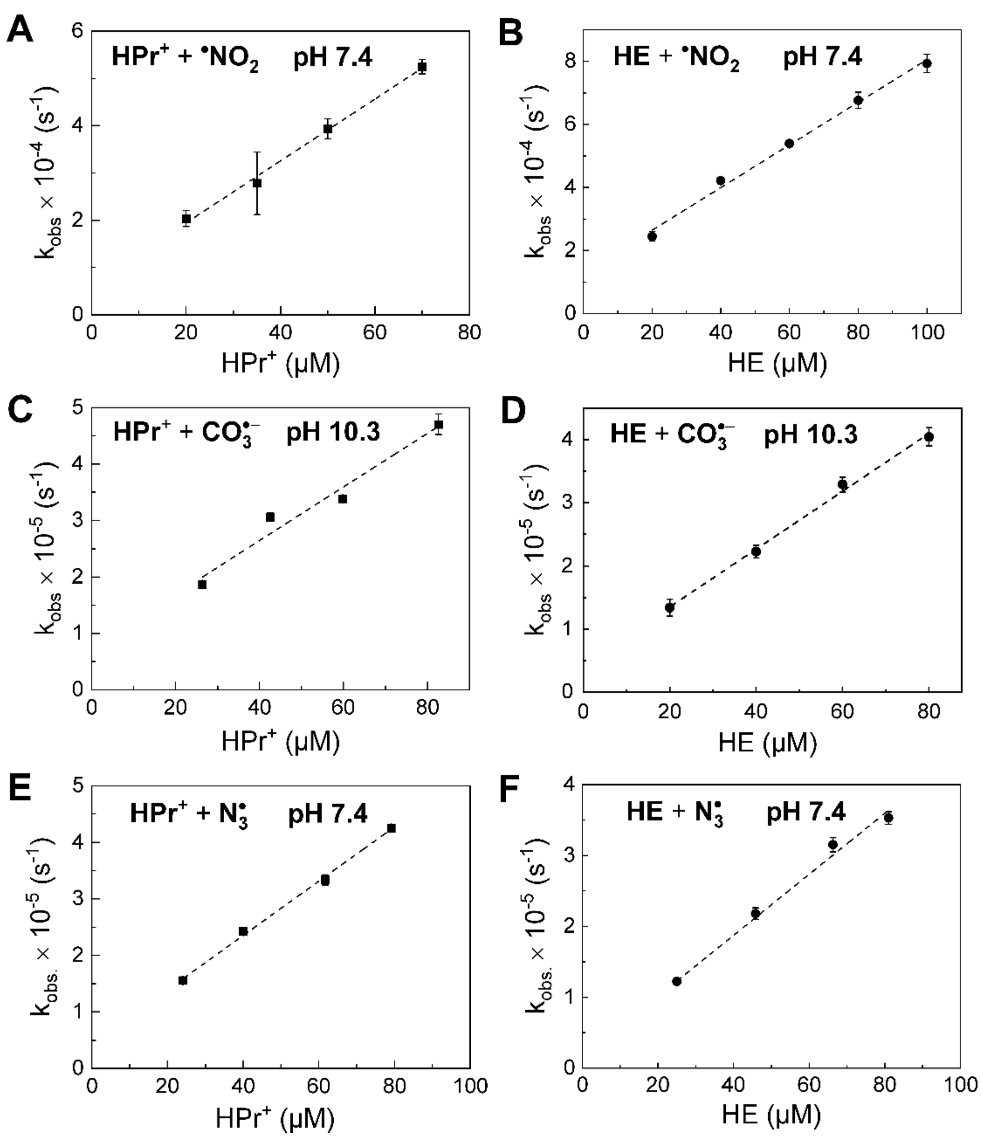

Supplementary Figure S1. The dependences of the pseudo-first-order rate constants of the reaction between probes and radical oxidants on the initial concentration of the probes. (A) Incubation mixtures contained 0.1 M $NaNO_3$, 1 M t-BuOH, 50 mM phosphate buffer at pH 7.4, 10 - 70 µM HPr^+, and were saturated with N_2. Radiation dose 20.5 Gy (B) same as (A) but incubation mixtures contained 10 - 100 µM HE instead of HPr^+. Radiation dose: 15 Gy. (C) Incubation mixtures contained 0.25 M Na_2CO_3, 0.25 M $NaHCO_3$ (pH 10.3), 10 - 80 µM HPr^+, and were saturated with N_2O. Radiation dose 20 Gy (D) same as (C) but incubation mixtures contained 10% MeCN and 10 - 80 µM HE. Radiation dose: 15 Gy. (E) The incubation mixtures contained 0.1 M NaN_3, 50 mM phosphate buffer at pH 7.4, 20 - 80 µM HPr^+, and were saturated with N_2O. Radiation dose: 20.7 Gy. (F) same as (E) but the reaction mixture contained 20 - 80 µM HE and 10% MeCN. Radiation dose: 7 Gy. The kinetics were determined by following the increase in the absorbance at 460 nm, corresponding to the formation of probes' radical cations. The non-zero intercepts observed for the kinetics of the reaction of HPr^+ with $N_3^\bullet$ and HE with $^\bullet NO_2$ can be explained by the fast self-decay of the listed radicals competing with the observed reaction of the probe and radical oxidant.

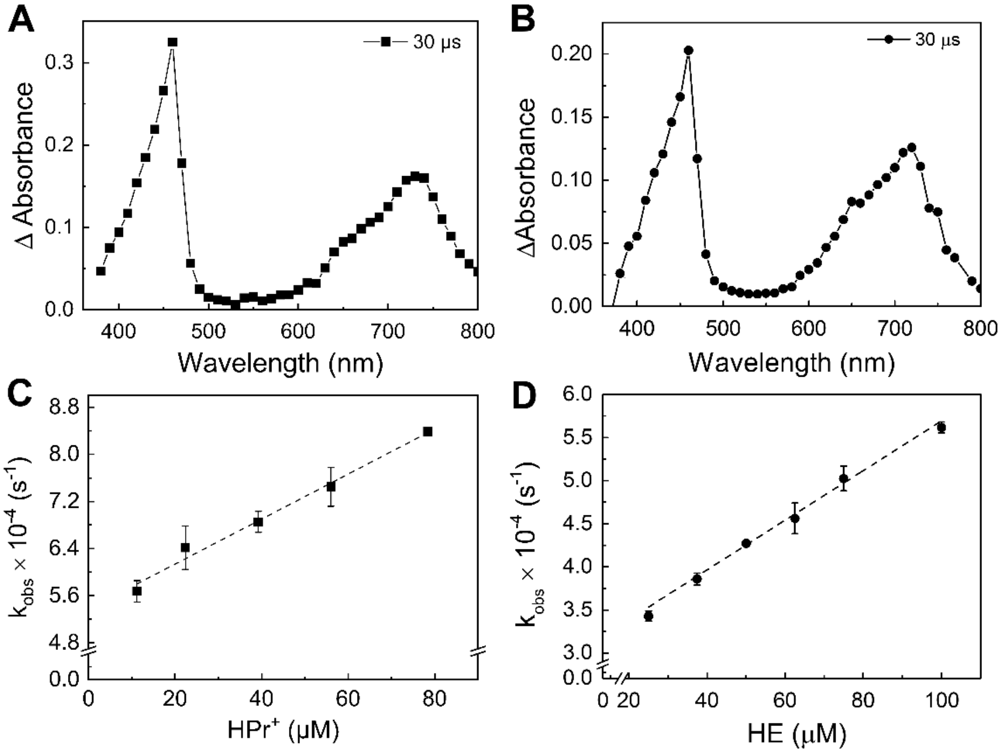

Supplementary Figure S2. The reactivity of $GS^{\bullet}$ toward HPr^{+} and HE. (A) Transient absorption spectra recorded after pulse radiolysis of N_2O-saturated solutions of 1.6 M CH_3OH, and 50 mM phosphate buffer at pH 7.4 containing 2.5 mM GSH and 50 µM HPr^{+} recorded 30 µs after electron pulse. Sample was 1 cm thick. Radiation dose: 54 Gy. (B) same as (A) but solution contained 60 mM phosphate buffer and 75 µM HE instead of HPr^{+}. Radiation dose: 65 Gy. (C) The relationship between the first-order rate constant and the concentration of HPr^{+} for the buildup of $HPr^{\bullet 2+}$ (monitored at 460 nm) in the reaction of HPr with $GS^{\bullet}$. Experimental conditions: aqueous solutions were saturated with N_2O and contained 50 mM phosphate buffer, 2.5 mM GSH, 1.6 M CH_3OH, pH 7.4. (D) same as (C) but solution contained (20 - 100 µM) HE instead of HPr^{+} and 10% MeCN.

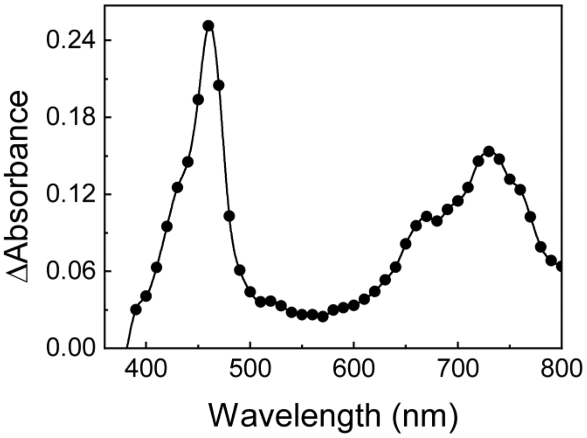

Supplementary Figure S3. Oxidation of HE by the $CH_2ClO_2^{\bullet}$ radical. Transient absorption spectrum recorded after pulse radiolysis of O_2-saturated solution of HE (125 µM) containing phosphate buffer (20 mM, pH 7.4) in H_2O:2-PrOH (1:1; v/v) mixture with 4% (v/v) of CH_2Cl_2, recorded 30 µs after pulse. Sample was 1 cm thick. Radiation dose: 65 Gy.

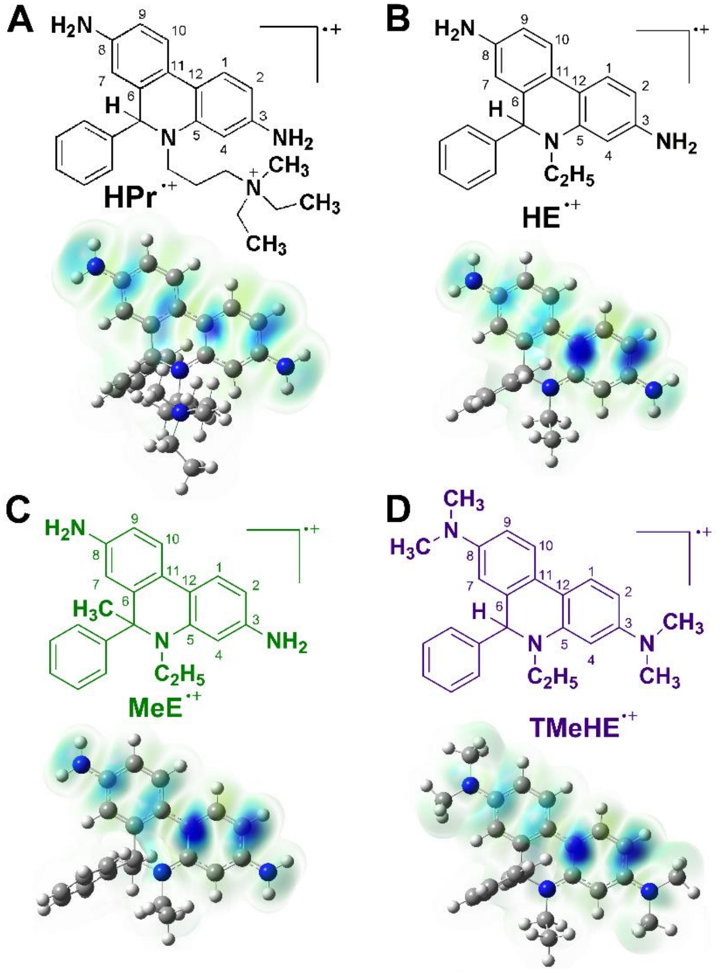

Supplementary Figure S4. Chemical structures of (A) $HPr^{\bullet 2+}$, (B) $HE^{\bullet +}$, (C) $MeE^{\bullet +}$, and (D) $TMeHE^{\bullet +}$, and appropriate spin density maps obtained from DFT quantum mechanical calculations using 6-311+G(d,p) basis set.

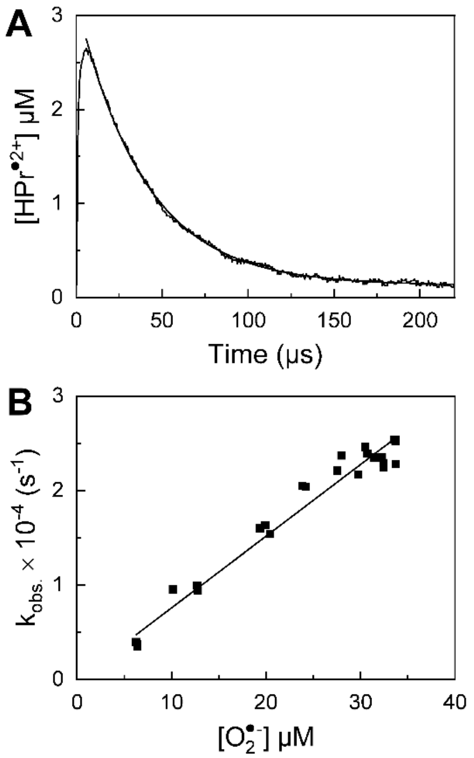

Supplementary Figure S5. (A) Decay of $HPr^{2+\bullet}$ due to the reaction with $O_2^{\bullet-}$ monitored at 460 nm. The kinetic trace was recorded after the pulse radiolysis of oxygen-saturated aqueous solution containing HPr^+ (200 μM), NaN_3 (3 mM), formate (50 mM), and phosphate buffer (5 mM, pH 7.4). Radiation dose: 61 Gy. The pseudo-first order kinetics fit (k_{obs}) to the experimental data is also shown. The concentration of HPr^+ was calculated using the previously determined extinction coefficient of 3.0×10^4 $M^{-1}cm^{-1}$. (B) Plot of k_{obs} against the initial concentration of $O_2^{\bullet-}$.

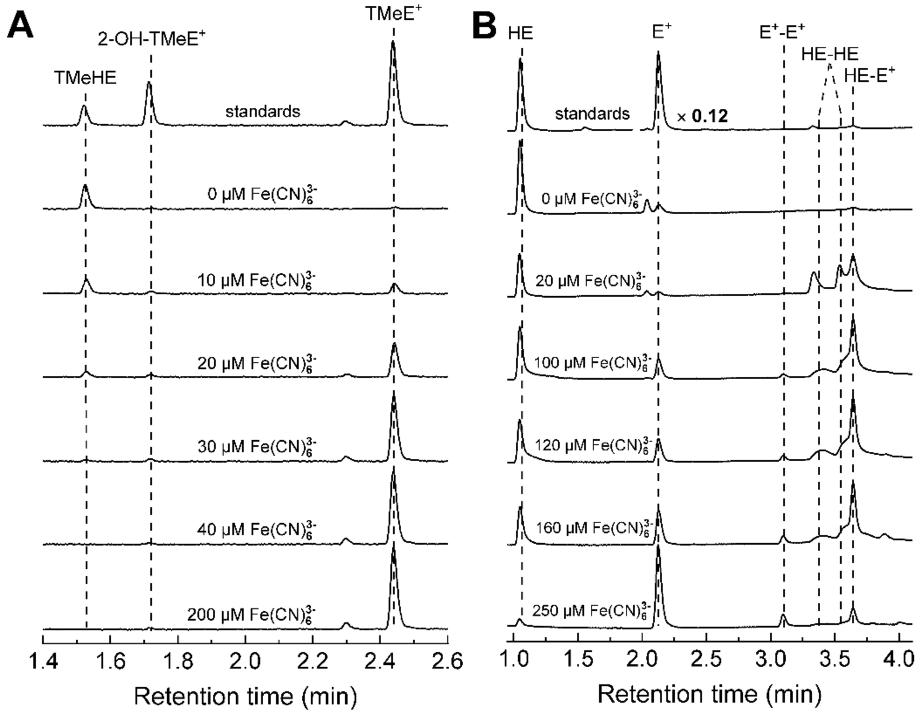

Supplementary Figure S6. Oxidation of TMeHE and HE by ferricyanide anion. (A) Chromatograms obtained from UPLC analyses of the mixtures containing TMeHE (20 µM) and various concentration of potassium ferricyanide (0, 10, 20, 30, 40, and 200 µM) in MeCN/phosphate buffer (50 mM; pH 7.4) (20:80 v/v) with dtpa (0.1 mM). Concentration of the standards was 20 µM, 5 µM, and 20 µM, for TMeHE, 2-OH-TMeE$^+$, TMeE$^+$, respectively. (B) same as (A) but mixtures contained 50 µM HE and the concentration of ferricyanide anion was in the range of 0 - 250 µM. Instead of MeCN, solutions contained 0.3% DMSO. Experimental conditions: temperature, 25°C; analytical wavelength for UV-Vis detection, λ = 370 ± 10 nm and λ = 270 ± 5 nm for TMeHE and HE, respectively. The UPLC trace of E$^+$ standard was scaled by a factor 0.12 to fit the E$^+$ and HE peak height to the same scale.

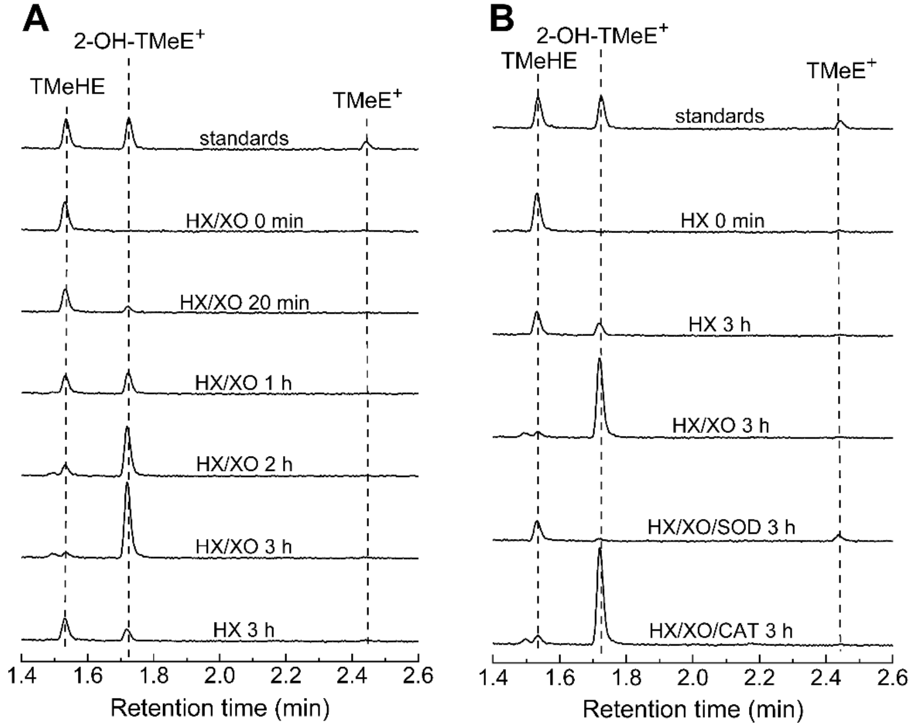

Supplementary Figure S7. Oxidation of TMeHE with $O_2^{\bullet-}$. (A) The increase of 2-OH-TMeE$^+$ concentration over time of superoxide production. (B) The influence of catalase and SOD on the oxidation of TMeHE by $O_2^{\bullet-}$. Chromatograms obtained from UPLC analyses of the mixtures containing TMeHE (20 µM), hypoxanthine (HX, 0.5 mM), xanthine oxidase (XO, 0.15 mU/ml), superoxide dismutase (SOD, 0.1 mg/ml), and catalase (CAT, 100 U/ml) in MeCN/phosphate buffer (50 mM; pH 7.4) (20:80 v/v) with dtpa (0.1 mM). Concentrations of the standards were 20 µM, 5 µM, and 5 µM, for TMeHE, 2-OH-TMeE$^+$, TMeE$^+$, respectively. Experimental conditions: incubation time as shown; temperature, 25°C; analytical wavelength for UV-Vis detection, λ = 370 ± 0.8 nm.

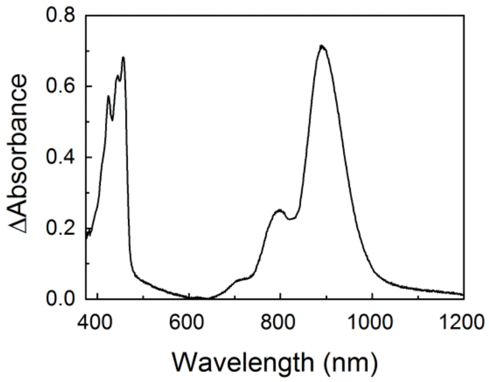

Supplementary Figure S8. Electronic absorption spectrum of benzidine radical cation generated by the irradiation of benzidine embedded in $BMIM^+PF_6^-$:CH_2Cl_2 (1:1, v/v) matrix at 77 K. Radiation dose: 32 kGy. Sample was ~ 1 mm thick.

Supplementary Table S1. Second-order rate constants for the reaction of HPr^+ and HE with various one-electron oxidants at pH 7.4.

One-electron oxidant	k_{HPr}[a] ($M^{-1}s^{-1}$)	k_{HE}[a] ($M^{-1}s^{-1}$)	$E^{\circ\prime}$[b] (V)
$HO^\bullet$	$(1.2 \pm 0.1) \times 10^{10}$	$(7 \pm 1) \times 10^{9}$[c]	2.31
$CO_3^{\bullet-}$[d]	$(4.8 \pm 0.6) \times 10^{9}$	$(4.6 \pm 0.2) \times 10^{9}$	1.57
$^\bullet NO_2$	$(6.5 \pm 0.3) \times 10^{8}$	$(6.8 \pm 0.3) \times 10^{8}$	1.04
$GS^\bullet$	$(3.8 \pm 0.3) \times 10^{8}$	$(2.9 \pm 0.1) \times 10^{8}$	0.92[e]
$CysS^\bullet$	$(2.4 \pm 0.1) \times 10^{8}$	$(4.3 \pm 0.2) \times 10^{8}$	0.92[f]
$N_3^\bullet$	$(4.8 \pm 0.2) \times 10^{9}$	$(4.2 \pm 0.3) \times 10^{9}$	1.33
$Br_2^{\bullet-}$	$(3.9 \pm 0.2) \times 10^{9}$	$(3.7 \pm 0.1) \times 10^{9}$	1.63

[a] k_{HPr} and k_{HE} were estimated under the pseudo-first order conditions using at least 10-times higher concentration of a probe than the oxidant formed by pulse radiolysis. The accurate conditions are given in SI. [b] Electrode potentials for pH 7 from Ref.[1] E° = E°' when no protons occur in the half-reaction. [c] Ref.[2] [d] pH 10.3 [e] E°'($GS^\bullet$, H^+/GSH) in water at pH 7.4.[3] [f] Assumed to be equal to $GS^\bullet$, H^+/GSH couple.

Supplementary Table S2. Second-order rate constants for the reaction of HE with chloromethylperoxyl radicals and $HO_2^\bullet$.

$RO_2^\bullet$	^2k ($RO_2^\bullet$ + HE) ($M^{-1}s^{-1}$)	E° ($RO_2^\bullet$/ RO_2^-)[a] (V)
$CCl_3O_2^\bullet$	$(1.23 \pm 0.02) \times 10^{9}$	1.44
$CHCl_2O_2^\bullet$	$(8.80 \pm 0.05) \times 10^{8}$	1.38
$CH_2ClO_2^\bullet$	$(2.74 \pm 0.06) \times 10^{8}$	1.23
$HO_2^\bullet$	$(10 \pm 2) \times 10^{6}$	0.77[b]

[a] Standard electrode potentials taken from the Table 2 of Ref.[4] [b] The standard electrode potential of the $HO_2^\bullet/HO_2^-$ couple was calculated using the pK_a value of hydrogen peroxide (11.7), the standard electrode potential of the $HO_2^\bullet$,H^+/H_2O_2 couple (1.46 V),[1] and 1 M concentration of H^+ (pH 0), according to the standard state conditions, using the following equation

$$E^{o}_{HO_2^\bullet,H^+/H_2O_2} = E^{o}_{HO_2^\bullet/HO_2^-} + 0.059(pK_a - pH)$$

Supplementary Table S3. The results obtained from TD-DFT calculations performed for $HE^{\bullet+}$, $MeE^{\bullet+}$, and $TMeHE^{\bullet+}$.

Mulliken spin densities from optimized geometries

	**$HPr^{\bullet 2+}$**	**$HE^{\bullet+}$**	**$MeE^{\bullet+}$**	**$TMeHE^{\bullet+}$**
**C-1**	-0.027	-0.077	-0.066	-0.062
**C-2**	0.135	0.189	0.180	0.167
**C-3**	0.061	0.040	0.040	0.024
**C-4**	-0.035	-0.044	-0.040	-0.033
**C-5**	0.031	0.020	0.019	0.008
**C-6**	0.050	0.086	0.086	0.051
**C-7**	0.017	-0.010	-0.010	0.009
**C-8**	0.095	0.102	0.095	0.076
**C-9**	0.015	-0.007	0.004	0.010
**C-10**	0.029	0.048	0.056	0.034
**C-11**	0.093	0.062	0.062	0.081
**C-12**	0.207	0.271	0.263	0.249
**N_α**	0.109	0.095	0.093	0.127
**N_β**	0.146	0.116	0.120	0.143

Experimental spectra–cryogenic measurements

$HPr^{\bullet 2+}$ Trans, nm	eV	ΔAbs	$HE^{\bullet+}$ Trans, nm	eV	ΔAbs	$MeE^{\bullet+}$ Trans, nm	eV	ΔAbs	$TMeHE^{\bullet+}$ Trans, nm	eV	ΔAbs
460	2.70	1.0658	460	2.70	1.1097	477	2.60	1.2480	458	2.71	1.1725
677	1.83	0.2574	670	1.85	0.2778	796	1.56	0.3735	673	1.84	0.3364
748	1.66	0.5718	737	1.68	0.5627	892	1.39	0.6252	737	1.68	0.6435

Predicted spectra–TD-DFT

$HPr^{\bullet 2+}$ Trans, nm	eV	f	$HE^{\bullet+}$ Trans, nm	eV	f	$MeE^{\bullet+}$ Trans, nm	eV	f	$TMeHE^{\bullet+}$ Trans, nm	eV	f
393	3.16	0.2180	393	3.15	0.2137	394	3.15	0.2593	402	3.09	0.4215
398	3.12	0.1508	399	3.11	0.1076	399	3.11	0.0656	425	2.92	0.0139
633	1.96	0.1058	610	2.03	0.0468	585	2.12	0.0242	749	1.66	0.2876
666	1.86	0.0775	640	1.94	0.1386	642	1.93	0.1633			

Supplementary Table S4. Kinetic and spectral data for $HE^{•+}$, $HPr^{•2+}$, and $TMeHPr^{•2+}$.

Radical cation ($R^{•+}$)	$λ_{max}$ (nm)	$ε$[a] ($M^{-1}cm^{-1}$)	k ($R^{•+}$ + $R^{•+}$)[b] ($M^{-1}s^{-1}$)	k ($R^{•+}$ + $O_2^{•-}$)[c] ($M^{-1}cm^{-1}$)
$HE^{•+}$	460	1.7×10^4	$(2.7 \pm 0.2) \times 10^8$	
$HPr^{•2+}$	460	3.0×10^4	$(3.3 \pm 0.1) \times 10^8$	$(7.4 \pm 0.1) \times 10^8$
$TMeHPr^{•2+}$	480	2.6×10^4		$(5.0 \pm 0.1) \times 10^8$

[a] Extinction coefficients used in calculations. [b] Rate constants for the observed second-order decay of $HE^{•+}$, $HPr^{•2+}$, and $TMeHPr^{•2+}$. [c] Rate constants for the reaction of $HPr^{•2+}$, and $TMeHPr^{•2+}$ with superoxide.

Supplementary Table S5. Mass spectral data for TMeHE and its oxidation products, $TMeE^+$ and 2-OH-$TMeE^+$.

Compound	Molecular ion	Ionic charge	Distance between isotopic peaks (m/z)	Calc. masses m/z	Calc. intensities (%)	Exp. masses (m/z)	Exp. intensities (%)
TMeHE	TMeE(+H^+)	1	1	372.244	100	372.244	100
				373.247	27	373.250	25
				374.240	4	374.253	4
$TMeE^+$	$TMeE^+$	1	1	370.228	100	370.224	100
				371.232	27	371.226	27
				372.234	4	372.228	4
2-OH-$TMeE^+$	2-OH-$TMeE^+$	1	1	386.223	100	386.219	100
				387.226	28	387.228	28
				388.229	4	388.230	4

Supplementary Table S6. Mass spectral data for $TMeHPr^{+}$ and its oxidation products, 2-OH-$TMeE^{++}$ and $TMeE^{++}$.

Compound	Molecular ion	Ionic charge	Distance between isotopic peaks (m/z)	Calc. masses (m/z)	Calc. intensities (%)	Exp. masses (m/z)	Exp. intensities (%)
$TMeHPr^{+}$	$TMeHPr^{+}$	1	1	471.3488	100.0	471.3812	100.0
				472.3521	34.5	472.3836	35.5
				473.3555	5.8	473.3862	7.9
2-OH-$TMePr^{++}$	2-OH-$TMePr^{++}$	2	0.5	243.1680	100.0	243.1811	100.0
				243.6696	34.5	243.6832	37.9
				244.1713	5.8	244.1845	7.8
	2-OH-$TMePr^{++}$ (+CF_3COO^-)	1	1	599.3209	100.0	599.3592	100.0
				600.3241	38.9	600.3618	41.4
				601.3271	8.0	601.3693	9.8
$TMePr^{++}$	$TMePr^{++}$	2	0.5	235.1705	100.0	235.1841	100.0
				235.6722	34.5	235.6854	36.7
				236.1739	5.8	236.1869	7.4
	$TMePr^{++}$ (+CF_3COO^-)	1	1	583.3260	100.0	583.3646	100.0
				584.3292	38.9	584.3674	38.6
				585.3322	7.8	585.3698	7.7

Synthesis of hydroethidine and hydropropidine derivatives

TMeHE and MeE were synthesized starting with the commercially available ethidium cation (E^+) in a two- or one-step sequence, respectively, as illustrated in Schemes 1 and 2. Methylation of ethidium bromide using trimethylphosphate was performed at 180°C, adapting the previously described procedure.[5] In this reaction the *N,N,N′,N′*-tetramethylated derivative was obtained in 65% yield as purple crystals (crystal structure depicted on page 39), which was subsequently reduced using sodium borohydride in methanol to afford TMeHE in quantitative yield (Scheme 1). MeE was obtained in 40% yield by reaction of E^+ with methyl magnesium bromide in a mixture of THF and CH_2Cl_2 for 3 days at 40°C (Scheme 2).

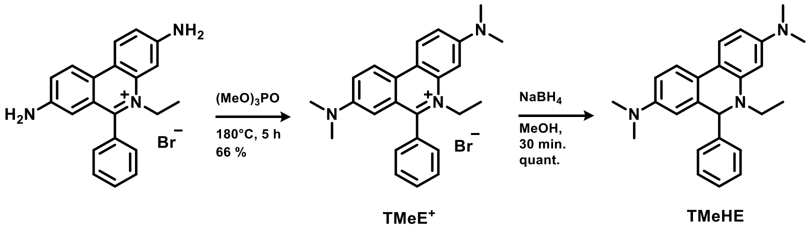

Scheme 1. Synthesis of TMeHE.

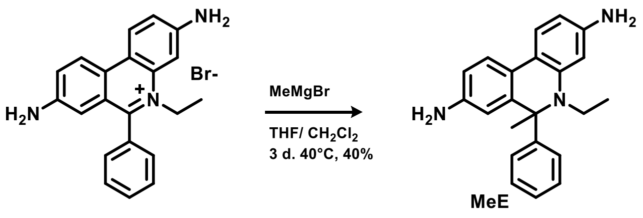

Scheme 2. Synthesis of MeE.

$TMeHPr^+$ was prepared using two different synthetic pathways (Scheme 3). The first pathway yielded $TMeHPr^+$ in four steps and 13% overall yield starting from 3,8-diamino-6-phenylphenanthridine. The second pathway yielded $TMeHPr^+$ in six steps, with an overall yield ~8%. The first synthetic sequence started with the reductive methylation of the two exocyclic amino groups producing compound **1** in 92% yield. 3-iodopropyl trifluoromethanesulfonate[6] was reacted with **1** in nitrobenzene to yield the phenanthridinium salt **2** in 16% yield.

The substitution of the triflate group in **2** by *N,N*-diethylmethylamine in methanol led to $TMePr^{++}$ in 85% yield. Finally, the reduction of $TMePr^{++}$ by sodium borohydride in MeOH led to $TMeHPr^+$ with a quantitative yield.

The second synthetic sequence started with the Cbz-protection of the two exocyclic amino functions to afford compound **3** in 90% yield. 3-iodopropyl trifluoromethanesulfonate[6] reacted with compound **3** in nitrobenzene, leading to the corresponding phenanthridinium cation **4** in 39% yield. The substitution of the halogen in **4** with *N,N*-diethylmethylamine in methanol led to the dication **5** in 75% yield. The deprotection of the carbamate groups by H_2 ($Pd(OH)_2$) afforded HPr^+ in 93% yield.[7] The methylation of the two exocyclic amino groups of HPr^+ in the presence of trimethylphosphate led to $TMePr^{++}$ in 32% yield.

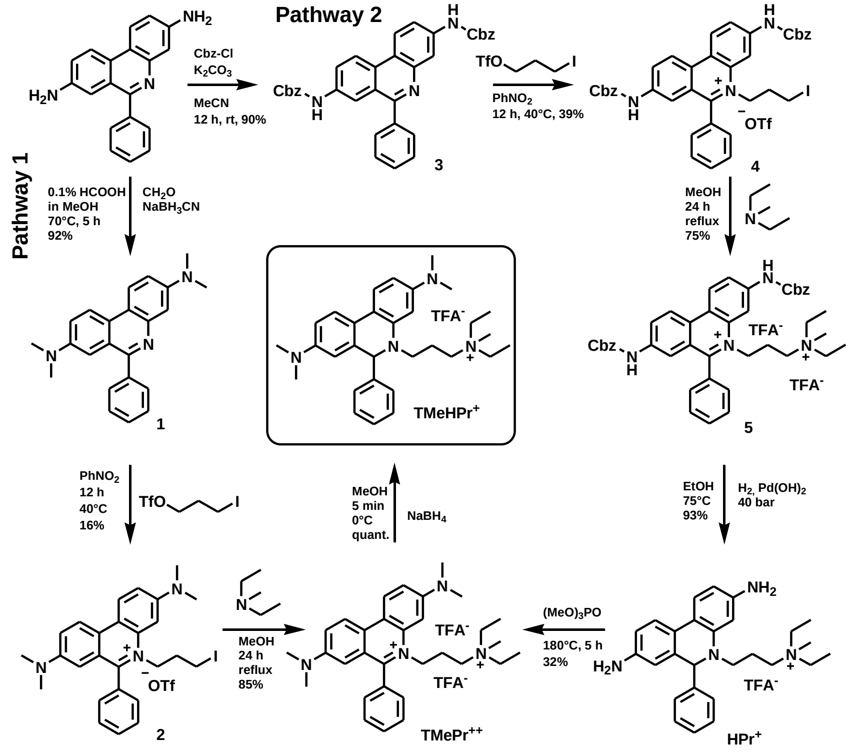

Scheme 3. Routes leading to $TMeHPr^+$.

The corresponding hydroxylated oxidation products of TMeHE and $TMeHPr^+$ have been prepared by reacting HE-based probes with Fremy's salt in DMSO/water mixture (Scheme 4).[7,8]

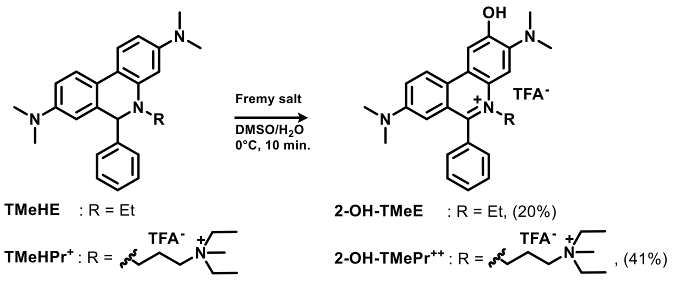

Scheme 4. Synthesis of 2-OH-$TMeE^+$ and 2-OH-$TMePr^{++}$.

Materials

Propidium iodide was from Sigma-Aldrich and ethidium bromide was from Alfa Aesar (Germany). HPr^+ and HE were synthesized by the reduction of propidium iodide (or compound **5**) and ethidium bromide, respectively, with sodium borohydride according to the procedures described elsewhere.[7-9] Dichloromethane (DCM) was obtained from POCH (Poland). Catalase (from *Corynebacterium glutamicum*) was from Fluka. All other reagents were of the highest purity available and were obtained from Sigma-Aldrich. All aqueous solutions were prepared using deionized water from a Milipore Mili-Q (Germany) system.

Synthetic procedures

All reactions were performed under argon atmosphere using oven-dried or flame-dried glassware and solvents were added *via* syringe. DCM was distilled under dry argon atmosphere in the presence of P_2O_5. All reagents were used as received without further purification. The reactions were monitored by TLC on silica gel Merck $60F_{254}$ and by HPLC/MS using a C_{18} reversed phase column. Crude materials were purified by flash chromatography on Merck Silica gel 60 (0.040-0.063 mm). ^{1}H NMR and ^{13}C NMR spectra were recorded with a Bruker DPX 300, 400 or 600 spectrometers at 300.13, 400.13, or 600.13 and 75.54, 100.61 or 150.90 MHz, respectively. Chemical shifts (δ) are reported in ppm and coupling constant J values in hertz. HRMS analyses were performed at the Spectropole of the Aix-Marseille Université on a QStar Elite (Applied Biosystems SCIEX) with API as an ionisation source. HPLC/MS experiments were performed using an Agilent 1260 infinity system coupled with a 6120 simple quadrupole. This system was equipped with a C_{18} column (Zorbax 1.8 µm, 3 mm × 50 mm) that was equilibrated with 10% vol. MeCN (containing 0.1% (v/v) formic acid) in 0.1% vol. formic acid aqueous solution at the flow rate of

0.28 or 0.40 mL/min. After sample injection, the MeCN mobile phase concentration was increased to 40% vol. over 3 min. Then, it was increased to 100% vol. over 8 min and maintained at this concentration over a period of 6 min. Hydrogenation of compounds was performed under continuous-flow conditions using a high-pressure hydrogenator (H-Cube Mini, Thales Nanotechnology Inc.).

3,8-Tetramethylamino-5-diethyl-6-phenylphenanthridinium ($TMeE^+$)

Ethidium bromide (600 mg, 1.5 mmol) was dissolved in 3 ml of trimethylphosphate and the mixture was heated for five hours at 180°C. After the mixture cooled to room temperature, water (10 ml) and ammonium bromide were added. The product was extracted by chloroform (20 ml); then, the organic phase was dried over Na_2SO_4. The solvent was removed under reduced pressure and the obtained solid was washed with diethyl ether. 500 mg of the product was obtained after purification by silica gel column chromatography (DCM/ethanol, 10/1 v/v). Crystals were obtained by slow infusion of pentane into the solution of **$TMeE^+$** in chloroform. $TMeE^+$ (450 mg, 1 mmol) was obtained in 66% yield. HMRS calculated for $[C_{25}H_{28}N_3]^+$, Br^-; $[C_{25}H_{28}N_3]^+$ 370.2278, found 370.2277. ^{1}H NMR ($CDCl_3$, 300 MHz): δ 8.56 (1H, d, *J* = 9.5), 8.54 (1H, d, *J* = 9.5), 7.72-7.74 (3H, m), 7.65 (1H, dd, *J* = 9.3, 2.6), 7.50-7.53 (2H, m), 7.43 (1H, dd, *J* = 9.5, 2.2), 7.35-7.73 (1H, d, *J* = 1.8), 6.19 (1H, d, *J* = 2.6), 5.10-5.12 (2H, q), 3.30 (6H, s), 2.90 (6H, s), 1.58 (3H, t). ^{13}C NMR ($CDCl_3$, 75 MHz): δ 158.7 (s), 151.5 (s), 148.7 (s), 134.6 (s), 132.0 (s), 131.1 (s), 129.7 (2C, s), 128.1 (2C, s), 127.8 (s), 125.4 (s), 124.9 (s), 124.3 (s), 122.6 (s), 117.8 (s), 117.3 (s), 107.8 (s), 98.0 (s), 50.2 (s), 40.7 (s), 39.9 (2C, s), 14.3 (2C, s).

3,8-Tetramethylamino-5-diethyl-6-phenylphenanthridine (TMeHE)

$TMeE^+$ (200 mg, 0.44 mmol) was dissolved in 5 ml of dry methanol under argon atmosphere. Then sodium borohydride (30 mg, 0.8 mmol) in dry methanol (5 ml) was added to the mixture drop by drop over a period of 10 min in the dark. The color changed immediately from purple to yellow. The reaction mixture was stirred for 30 min at room temperature under argon atmosphere. Then 100 ml $CHCl_3$ was added to the mixture, and the solution was washed with H_2O (20 ml) and brine (20 ml) and dried with Na_2SO_4. The solvent was removed under reduced pressure. TMeHE (165 mg, 0.44 mmol) was obtained in quantitative yield. HMRS calculated for $C_{25}H_{29}N_3$; $[C_{25}H_{29}N_3 + H]^+$, 372.2434, found 372.2433. ^1H NMR ($CDCl_3$, 300 MHz): δ 7.82 (1H, d, J = 8.6), 7.80 (1H, d, J = 8.6), 7.42-7.53 (5H, HPh, m), 6.95 (1H, dd, J = 8.6, 2.6), 6.69 (1H, d, J = 2.6), 6.52 (1H, dd, J = 8.4, 2.4), 6.35 (1H, d, J = 2.4), 5.66 (1H, s), 3.73 (1H, m), 3.51 (1H, m), 3.24 (6H, s), 3.16 (6H, s), 1.48 (3H, t). ^{13}C NMR ($CDCl_3$, 75 MHz): δ 150.8 (s), 148.8 (s), 144.1 (s), 143.9 (s), 135.2 (s), 128.4 (2C, s), 127.0 (s), 126.7 (2C, s), 122.9 (s), 122.4 (s), 120.6 (s), 112.78 (s), 112.7 (s), 110.7 (s), 102.4 (s), 97.2 (s), 66.4 (s), 44.4 (s), 40.9 (2C, s), 40.7 (2C, s), 12.9 (s).

3,8-Tetramethylamino-5-diethyl-2-hydroxy-6-phenylphenanthridium (2-OH-TMeE⁺)

To a cooled solution (0°C) containing TMeHE (50 mg, 0.35 mmol) in DMSO (1.35 mL) 3.3 mL of a Fremy's salt solution in water (0.1 M) was added rapidly. The resulting mixture was vortexed and shielded from light. After 10 min, the crude material was centrifuged and the supernatant was purified by semi-preparative HPLC, using a Kinetex C_{18} column (Phenomenex, 5 µm, 250 mm × 21.2 mm) equilibrated with 10% vol. MeCN (containing 0.1% [v/v] TFA) in 0.1% TFA aqueous solution. The compounds were eluted by increasing the content of MeCN from 10% to 100% over 17 min at a flow rate of 13 mL/min. The pure fractions collected at 11 min were combined to

afford **2-OH-TMeE$^+$** as a red solid (10 mg, 20%). Crystals of **2-OH-TMeE$^+$** were obtained by slow infusion of pentane into the solution of **2-OH-TMeE$^+$** in DCM. **^{1}H NMR** ($CDCl_3$, 300 MHz): δ 9.21 (1H, s), 8.84 (1H, d, *J* = 9.3 Hz), 8.83 (1H, s), 7.81-7.74 (3H, m), 7.72 (1H, dd, *J* = 9.3 Hz, *J* = 2.7 Hz), 7.48-7.43 (2H, m), 6.24 (1H, d, *J* = 2.7 Hz), 4.78 (2H, q, *J* = 7.1 Hz), 3.42 (6H, s), 2.94 (6H, s), 1.56 (3H, t, *J* = 7.1 Hz). **^{13}C NMR** ($CDCl_3$, 75 MHz): δ 158.1, 154.3, 150.4, 135.1, 131.6, 131.2, 129.9, 128.2, 127.8, 127.1, 125.1, 124.7, 124.5, 113.2, 110.6, 108.5, 50.3, 43.8, 39.7, 15.0.

3,8-Tetramethylamino-6-phenylphenanthridine (1)

To a solution of 3,8-diamino-6-phenylphenanthridine (50 mg, 0.18 mmol) in 0.1% formic acid in methanol (1 mL) formaldehyde (37% in water, 0.42 mL) was added at 0°C under inert atmosphere. The reaction mixture was stirred for 5 h at 70°C. Then $NaBH_3CN$ (66 mg, 1 mmol) in methanol (1 mL) was added to the stirring mixture. After 1 h, the reaction mixture was cooled to room temperature. The crude material was diluted in DCM and extracted with brine. The combined organic layers were dried over Na_2SO_4, filtrated and concentrated to give the compound **1** as a pale yellow solid (55 mg, 92% yield). **ESI-MS** for [$C_{23}H_{23}N_3$], $[MH]^+$, 342.2.

^{1}H NMR (CD_3OD, 400 MHz): δ 8.49 (1H, d, *J* = 9.2 Hz), 8.41 (1H, d, *J* = 8.8 Hz), 7.71-7.67 (2H, m), 7.63-7.56 (3H, m), 7.50 (1H, dd, *J* = 9.2 Hz, *J* = 2.7 Hz), 7.34-7.26 (2H, m), 7.05 (1H, d, *J* = 2.7 Hz), 3.10 (6H, s), 2.95 (6H, s). **^{13}C NMR** (CD_3OD, 100 MHz): δ 160.9, 150.3, 148.5, 143.1, 139.9, 129.1, 128.4, 128.0, 125.5, 124.9, 122.1, 121.8, 119.5, 115.6, 115.4, 108.33, 108.25, 39.5, 39.3.

3-Iodopropyl-trifluoromethanesulfonate

3-Iodopropyl-trifluoromethanesulfonate (3-iodopropyltriflate) was prepared according to the literature method.[10] To a solution of 3-iodopropanol (0.4 g, 2.1 mmol) in DCM (8 mL), lutidine (0.35 mL, 3.0 mmol) and trifluoromethanesulfonic anhydride (0.43 mL, 2.6 mmol) were added at

-15°C under inert atmosphere. The reaction mixture was then allowed to stir at 0°C. After 1 h, 2 mL of NH_4Cl and 100 mL of DCM were added to the reaction mixture. The organic layer was washed twice with NH_4Cl sat. (25 mL), twice with brine (50 mL), dried over Na_2SO_4 and distilled under reduced pressure. Purification of the crude material by flash chromatography (pentane/AcOEt: 90/10 vol.) afforded 3-iodopropyl-trifluoromethanesulfonate as a colorless oil (0.3 g, 43% yield). **^{1}H NMR** ($CDCl_3$, 400 MHz): δ 4.64 (2H, t, *J* = 5.9 Hz), 3.27 (2H, t, *J* = 6.7 Hz), 2.33 (2H, quint., *J* = 6.1 Hz)

3,8-Tetramethylamino-5-(3-iodopropyl)-6-phenylphenanthridinium (2)

To a solution of phenanthridine **1** (475 mg, 1.39 mmol) in nitrobenzene (5.5 mL) 3-iodopropyltriflate (487 mg, 1.53 mmol) in nitrobenzene (1.5 mL) was added at 30°C under inert atmosphere. The reaction mixture was stirred overnight at 40°C. The product was purified by flash chromatography (DCM/EtOH: 98/2 vol.) to afford the phenanthridinium salt **2** as a blue solid (148 mg, 16% yield). **ESI-MS** for $C_{26}H_{29}IN_3^+$ $[M]^+$, 510.4. **^{1}H NMR** ($CDCl_3$, 400 MHz): δ 8.57 (1H, d, *J* = 9.4 Hz), 8.52 (1H, d, *J* = 9.4 Hz), 7.79-7.75 (3H, m), 7.67 (1H, dd, *J* = 9.4 Hz, *J* = 2.2 Hz), 7.58-7.55 (2H, m), 7.45 (1H, dd, *J* = 9.4 Hz, *J* = 1.3 Hz), 7.12 (1H, d, *J* = 1.3 Hz), 6.20 (1H, d, *J* = 2.2 Hz), 4.90 (2H, t, *J* = 8.2 Hz), 3.27 (6H, s), 3.19 (2H, t, *J* = 6.4 Hz), 2.90 (6H, s), 2.53-2.43 (m, 2H). **^{13}C NMR** ($CDCl_3$, 100 MHz): δ 159.2, 151.6, 148.8, 134.9, 131.7, 131.3, 130.0, 128.6, 128.1, 125.9, 125.1, 124.6, 122.6, 118.0, 117.5, 107.6, 96.8, 54.8, 40.9, 39.9, 31.4, 0.59.

3,8-Tetramethylamino-5-[3-(diethylmethylammonio)propyl]-6-phenylphenanthridinium ($TMePr^{++}$)

To a solution of phenanthridinium salt **2** (100 mg, 0.15 mmol) in MeOH (6 mL), *N,N*-diethylmethylamine (0.37 mL, 3.00 mmol) was added. The reaction mixture was then refluxed for

1 day under inert atmosphere and monitored by HPLC. After total conversion, the reaction was cooled to room temperature and concentrated under reduced pressure. The crude material was purified by flash chromatography on a reversed phase C_{18} column (0.1% TFA in H_2O/MeCN from 90/10 vol. to 72/28 vol.) to afford **TMePr^{++}** as a deep blue solid (90 mg, 85%). **HRMS-ESI** m/z calc. for $C_{31}H_{42}N_4^{2+}$ $[M]^{2+}$ 235.1699, found 235.1699. **^{1}HNMR** (CD_3OD, 400 MHz): *δ* 8.78 (1H, d, *J* = 9.5 Hz), 8.70 (1H, d, *J* = 9.4 Hz), 7.88-7.81 (4H, m), 7.75-7.71 (2H, m), 7.63 (1H, dd, *J* = 9.5 Hz, *J* = 2.0 Hz), 7.08 (1H, d, *J* = 2.0 Hz), 6.25 (1H, d, *J* = 2.5 Hz), 4.79 (2H, t, *J* = 7.8 Hz), 3-34-3.30 (4H, m), 3.28 (s, 6H), 3.25 (2H, t, *J* = 9.5 Hz), 2.94 (3H, s), 2.90 (6H, s), 2.45-2.35 (2H, m), 1.25 (6H, t, *J* = 7.2 Hz). **^{13}C NMR** (CD_3OD, 100 MHz): δ 161.2, 153.4, 150.7, 136.3, 133.7, 132.8, 131.3, 129.81, 129.79, 127.6, 126.9, 126.2, 124.0, 119.4, 119.2, 108.7, 97.3, 58.0, 57.3, 51.2, 47.9, 40.8, 40.1, 22.7, 8.2.

3,8-Tetramethylamino-5-[3-(diethylmethylammonio)propyl]-6-phenylphenanthridine (TMeHPr$^+$)

To a solution of **TMePr^{++}** (53 mg, 76 µmol) in methanol (10 mL) at 0°C a solution of $NaBH_4$ (3.2 mg, 84 µmol) in methanol (1 mL) was added dropwise. The resulting mixture was vortexed and shielded from light. After 5 min, the crude material was concentrated under reduced pressure then dissolved in 1 mL of H_2O. The crude material was purified by flash chromatography on a reverse phase C_{18} (0.1% TFA in H_2O/MeCN from 90/10 to 50/50) afforded **TMeHPr$^+$** (44.6 mg, quant.). **HRMS-ESI** m/z calc. for $C_{31}H_{43}N_4^+$ $[M]^+$ 471.3482, found 471.3486. **^{1}H NMR** (D_2O, 600 MHz): δ 8.08 (H_{10}, d, *J* = 8.6 Hz), 8.04 (H_1, d, *J* = 8.6 Hz), 7.56 (H_9, dd, *J* = 8.6 Hz, *J* = 2.5 Hz), 7.49 (H_7, d, *J* = 2.5 Hz), 7.32-7.28 (3H, m), 7.26-7.23 (2H, m), 7.05 (H_2, dd, *J* = 8.6 Hz, *J* = 2.3 Hz), 6.93 (H_4, d, *J* = 2.3 Hz), 5.77 (1H, s), 3.87 (1H, td, *J* = 15.1 Hz, *J* = 5.6 Hz), 3.56 (1H, ddd, *J* = 15.1 Hz, *J* = 8.6 Hz, *J* = 5.6 Hz), 3.31-3.25 (1H, m), 3.29 (6H, s), 3.24 (6H, s), 3.22-3.14 (5H, s), 2.81 (3H, s), 2.17-

2.07 (1H, m), 2.03-1.94 (1H, m), 1.12 (3H, t, *J* = 7.2 Hz), 1.11 (3H, t, *J* = 6.9 Hz). **^{13}C NMR** (D_2O, 150 MHz): δ 165.9 (q, *J* = 35.3 Hz), 147.4 (C_3), 147.3 (C_{11}), 146.0 (C_8), 144.3, 139.9 (C_{14}), 132.3 (C_{13}), 132.1, 131.5, 129.3, 129.1 (C_1), 128.5 (C_{10}), 125.1 (C_{12}), 122.6 (C_9), 120.8 (C_7), 119.3 (q, *J* = 291.6 Hz), 107.4 (C_4), 68.1 (C_6), 60.6, 59.6, 59.5, 49.8, 49.4, 48.8, 48.5, 22.6, 9.7.

3,8-Bis-benzyloxycarbonylamino-6-phenylphenanthridine (3)

To a suspension of 3,8-diamino-6-phenylphenanthridine (0.8 g, 2.8 mmol) and potassium carbonate (0.8 g, 5.8 mmol) in MeCN (15 mL), benzyl chloroformate (1.1 mL, 7.7 mmol) was added dropwise at 0°C under inert atmosphere. The reaction mixture was stirred overnight at room temperature. The crude material was filtered, washed with MeCN and dissolved in THF. After concentration under reduced pressure, the crude product was dissolved in DCM and filtered. The filtrate was dried over Na_2SO_4 and the solvent was distilled under reduced pressure. Distillation of the volatiles under reduced pressure (10^{-3} Torr) afforded compound **3** as a pale yellow solid (1.4 g, 90% yield). **ESI-MS** for [$C_{35}H_{27}N_3O_4$], [MH]$^+$, 554.4. **^{1}H NMR** (DMSO d_6, 300 MHz) δ 10.18 (2H, 2s), 8.76 (1H, d, *J* = 9.26 Hz), 8.67 (1H, d, *J* = 9.07 Hz), 8.31 (1H, d, *J* = 2.08 Hz), 8.25 (1H, d, *J* = 2.27 Hz), 8.01 (1H, dd, *J* = 2.08 Hz, *J* = 9.07), 7.83 (1H, dd, *J* = 2.27 Hz, *J* = 9.26 Hz), 7.75-7.69 (2H, m), 7.82-7.58 (3H, m), 7.52-7.32 (10H, m), 5.23 (2H, s), 5.16 (2H, s). **^{13}C NMR** (DMSO d_6, 75 MHz) *δ* 161.3, 153.3, 143.3, 139.4, 138.0, 136.4, 135.9, 135.8, 129.6, 129.5, 128.8, 128.7, 128.5, 128.4, 128.3, 126.9, 125.3, 123.1, 122.8, 122.7, 119.6, 119.1, 117.9, 116.8, 67.9, 67.2.

3,8-Bis-benzyloxycarbonylamino-5-(3-iodopropyl)-6-phenylphenanthridinium (4)

To a solution of phenanthridine **3** (0.25 g, 0.45 mmol) in nitrobenzene (2 mL) 3-iodopropyltriflate (0.72 g, 2.3 mmol) in nitrobenzene (0.5 mL) was added at 30°C under inert atmosphere. The reaction mixture was stirred at 40°C overnight. The product was purified by chromatography column using (DCM/EtOH: 98/2 vol.) to afford compound **4** as a deep yellow solid (0.15 g, 39% yield). **ESI-MS** for $C_{38}H_{33}IN_3O_4^+$, $[M]^+$, 722.4. **^{1}H NMR** (CD_3OD, 400 MHz): δ 8.97 (1H, d, *J* = 9.2 Hz), 8.91 (1H, d, *J* = 9.2 Hz), 8.88 (1H, s), 8.28 (1H, dd, *J* = 9.2 Hz, *J* = 2.1 Hz), 7.96 (1H, dd, *J* = 9.2 Hz, *J* = 1.2 Hz), 7.91 (1H, d, *J* = 2.1 Hz), 7.88-7.83 (3H, m), 7.73-7.68 (2H, m), 7.50-7.46 (2H, m), 7.44-7.30 (8H, m), 5.29 (2H, s), 5.14 (2H, s), 4.87 (2H, m), 3.29 (2H, t, *J* = 6.3 Hz), 2.56-2.46 (2H, m).

3,8-Bis-benzyloxycarbonylamino-5-[3-(diethylmethylammonio)propyl]-6-phenylphenanthridinium (5)

To a solution of phenanthridinium salt **4** (0.15 g, 0.18 mmol) in MeOH (10 mL) *N,N*-diethylmethylamine (0.1 mL, 0.9 mmol) was added. The reaction mixture was then refluxed for 1 day under inert atmosphere and monitored by HPLC. After total conversion, the reaction was cooled to room temperature and concentrated. The crude material was purified by flash chromatography on a reversed phase C_{18} column (0.1% TFA in H_2O/MeCN 90/10 vol.) to afford phenanthridinium salt **5** as a yellow solid (126 mg, 75%). **ESI-MS** for $C_{43}H_{46}N_4O_4^{2+}$, $[M]^{2+}$, 341.4. **^{1}H NMR** (CD_3OD, 400 MHz): δ 10.53 (1H, s), 10.03 (1H, s), 9.10-9.08 (1H, m), 9.03 (1H, d, *J* = 9.2 Hz), 8.96 (1H, d, *J* = 9.3 Hz), 8.30 (1H, dd, *J* = 9.3 Hz, *J* = 2.3 Hz), 7.96 (1H, d, *J* = 2.3 Hz), 7.92-7.78 (4H, m), 7.75-7.70 (2H, m), 7.47-7.29 (10H, m), 5.32 (2H, s), 5.14 (2H, s), 4.85-4.77 (2H, m), 4.85-4.79 (6H, m), 2.97 (3H, m), 2.55-2.40 (2H, m), 1.29 (6H, t, *J* = 7.2 Hz). **^{1}H NMR** ($CDCl_3$, 400 MHz): δ 9.45,

8.83 (1H, d, J = 9.0 Hz), 8.73 (1H, d, J = 9.0 Hz), 8.68 (1H, d, J = 9.1 Hz), 8.54-8.37 (2H, m), 7.84-7.77 (3H, m), 7.76-7.69 (2H, m), 7.50-7.45 (4H, m), 7.44-7.33 (8H, m), 5.28 (2H, s), 5.17 (2H, s), 5.0-4.8 (2H, m), 3.55-3.40 (2H, m), 3.40-3.27 (4H, m), 2.97 (3H, s), 2.64-2.49 (2H, m), 1.32-1.20 (6H, m). **^{13}C NMR** ($CDCl_3$, 100 MHz): δ 163.1, 153.7, 143.2, 139.4, 135.5, 135.2, 133.8, 132.1, 131.2, 130.3, 130.0, 128.63, 128.59, 128.5, 128.4, 128.13, 128.10, 128.0, 125.3, 123.3, 122.3, 121.3, 106.4, 67.54, 67.48, 57.1, 56.9, 51.1, 47.4, 22.1, 7.8.

3,8-Diamino-5-[3-(diethylmethylammonio)propyl]-6-phenylphenanthridine (HPr$^+$)

Hydrogenation of compound **5** under continuous-flow conditions was performed using a high-pressure hydrogenator. The dication **5** (126 mg, 0.14 mmol) in EtOH (10 mL) was introduced into the reactor by a pump at 75°C, 40 bar of hydrogen pressure with a flow rate of 1 mL/min using a $Pd(OH)_2$ CatCart®. The reaction was monitored by LC-MS. After total conversion, the crude material was concentrated under vacuum. Purification of the crude material by flash chromatography on a reversed phase C_{18} column (0.1% TFA in H_2O/MeCN 90/10 vol.) afforded **HPr$^+$** as a white powder (68.2 mg, 93%).

3,8-Tetrametylamino-5-[3-(diethylmethylammonio)propyl]-6-phenylphenanthridinium (TMePr^{++})

HPr$^+$ (98 mg, 0.19 mmol) was dissolved in trimethyl phosphate (2.2 mL, 18.5 mmol) and heated for 5 hours at 180°C. The reaction mixture was cooled to room temperature, washed with Et_2O, dissolved with DCM (supplemented with 5% of ethanol) and concentrated under reduced pressure. The crude material was purified by flash chromatography on a reverse phased C_{18} column (0.1% TFA in H_2O/MeCN from 90/10 to 72/28 vol.) to afford the **TMePr^{++}** as a deep blue solid (42 mg, 32%).

3,8-Tetramethylamino-5-[3-(diethylmethylammonio)propyl]-2-hydroxy-6-phenylphenanthridine (2-OH-TMePr^{++})

To a solution containing **TMeHPr^{+}** (5.0 mg, 8.5 µmol) in DMSO (342 µL) at 0°C, 342 µL of a Fremy's salt solution in water (0.1 M) was rapidly added. The resulting mixture was vortexed and shielded from light. After 10 min, the crude material was centrifugated and the supernatant was purified by semi-preparative HPLC using a Kinetex C_{18} column (Phenomenex, 5 µm, 250 mm × 21.2 mm) equilibrated with 25% vol. MeCN (containing 0.1% [v/v] TFA) in 0.1% (v/v) TFA aqueous solution. The compounds were eluted by increasing the content of MeCN from 25% to 45% (v/v) over 13 min at a flow rate of 10 mL/min. The pure fractions collected at 11 min were combined to afford **2-OH-TMePr^{++}** as a red solid (2.5 mg, 41%). **HRMS-ESI** m/z calc. for $C_{31}H_{42}N_4O^{2+}$ $[M]^{2+}$ 243.1674, found 243.1679. **^{1}H NMR** (CD_3OD, 400 MHz): δ 8.60 (H_{10}, d, *J* = 9.4 Hz), 8.1 (H_1, s), 7.88-7.83 (3H, H_9, m), 7.76-7.72 (2H, m), 7.42 (H_4, s), 6.33 (H_7, d, *J* = 2.6 Hz), 4.75-4.69 (2H, m), 3.37-3.30 (4H, m), 3.27 (2H, t, *J* = 8.6 Hz), 3.16 (6H, s), 2.97 (3H, s), 2.94 (6H, s), 2.47-2.37 (2H, m), 1.28 (6H, t, *J* = 7.3 Hz). **^{13}C NMR** (CD_3OD, 100 MHz): δ 157.9 (C_6), 154.6 (C_2), 151.3 (C_8), 147.4 (C_3), 133.6, 132.8, 131.3, 130.0, 129.5 (C_{11}), 127.8 (C_{14}), 127.2 (C_{13}), 126.3 (C_9), 124.43 (C_{10}), 124.35 (C_{12}), 109.0 (C_7), 107.3 (C_1), 106.1 (C_4), 58.1, 57.3, 51.5, 48.0, 43.2, 40.1.

Synthesis of methylethidine (MeE).

To a suspension of ethidium bromide (50 mg, 0.27 mmol) in 5 ml of DCM 700 µL solution of methylmagnesium bromide (1 M in THF was added at 0°C. The reaction mixture was stirred for 3 days at 40°C under argon atmosphere. After the mixture was cooled to room temperature, water and solid ammonium bromide were added. The product was extracted by chloroform (2 × 20 ml), and the organic phase was dried on Na_2SO_4. The solvent was removed under reduced pressure.

Purification by C_{18} reverse phase semipreparative-HPLC using a gradient of acetonitrile/water containing 0.1% vol. TFA with a flow rate of 2.4 ml/min (detection at 230 nm) was performed to obtain MeE (20 mg, 0.05 mmol, 40%). ESI-MS calculated for $[C_{22}H_{23}N_3+H]^+$; 330.1965; found 330.1960.

Pulse radiolysis experiments.

Pulse radiolysis experiments were performed with a 6-MeV linear accelerator at the Institute of Applied Radiation Chemistry (Lodz, Poland). The radiolysis of water produces short-lived, highly reactive species, i.e., e_{aq}^- (2.6), $HO^•$ (2.7), and $H^•$ (0.6), and less-reactive intermediates, i.e., H_2O_2 (0.7), H_2 (0.45), H_3O^+ (2.6). The numbers in parentheses indicate the radiation yield values defined as the number of species formed per 100 eV energy absorbed.[11] The dose absorbed per pulse was measured on the basis of the initial yield of $(SCN)_2^{•-}$ formed in the nitrous oxide (N_2O)-saturated 0.01 M potassium thiocyanate (KSCN) aqueous solution, assuming a radiation yield of 6.13 and an extinction coefficient of 7580 $M^{-1}cm^{-1}$ at 475 nm.[12] The addition of aprotic polar solvent (MeCN) to aqueous solutions was required to achieve better solubility of HE. The presence of MeCN at concentrations up to 10% did not significantly affect the formation of one-electron oxidants in this system. In all kinetic experiments, to maintain pseudo-first-order conditions the concentration of HPr^+ and HE was at least in a five-fold excess to the appropriate radiolytically formed oxidant.

Generation of azidyl radical ($N_3^•$)

To investigate the reaction between HPr^+ or HE and the azidyl radical ($N_3^•$), e_{aq}^- was converted into $HO^•$ by saturating the solution with N_2O (Eq. 1, $k_1 = 9.1 \times 10^9$ $M^{-1}s^{-1}$).[13]

$$e_{aq}^{-} + N_2O + H_2O \rightarrow HO^{\bullet} + OH^{-} + N_2 \qquad \text{(Equation 1)}$$

Pulse radiolysis was carried out in the presence of sodium azide (NaN_3, 0.1 M), so that the azide ion reacted with $HO^{\bullet}$ to form $N_3^{\bullet}$ (Eq. 2, $k_2 = 1.2 \times 10^{10}$ $M^{-1}s^{-1}$).[13]

$$N_3^{-} + HO^{\bullet} \rightarrow N_3^{\bullet} + OH^{-} \qquad \text{(Equation 2)}$$

The azidyl radical is characterized by a narrow absorption band at 274 nm. It is a suitable one-electron oxidant for the investigation of reactive intermediates that absorb light at wavelengths longer than 300 nm.[14] Although the standard reduction potential of the azidyl radical (1.32 V) is lower than that of the dibromide radical anion ($Br_2^{\bullet-}$, 1.63 V), in most cases it reacts faster than $Br_2^{\bullet-}$ due to the relatively fast self-exchange reaction (Eq. 3, $k_3 = 4 \times 10^{4}$ $M^{-1}s^{-1}$).[15,16]

$$N_3^{\bullet} + N_3^{-} \rightarrow N_3^{-} + N_3^{\bullet} \qquad \text{(Equation 3)}$$

Generation of carbonate radical anion ($CO_3^{\bullet-}$)

To generate the $CO_3^{\bullet-}$, pulse radiolysis was carried out in N_2O-saturated 0.25 M sodium bicarbonate ($NaHCO_3$) and 0.25 M sodium carbonate (Na_2CO_3) solution at pH 10.

$$HCO_3^{-} + HO^{\bullet} \rightarrow CO_3^{\bullet-} + H_2O \qquad \text{(Equation 4)}$$

$$CO_3^{2-} + HO^{\bullet} \rightarrow CO_3^{\bullet-} + OH^{-} \qquad \text{(Equation 5)}$$

The second-order rate constants for reactions described by Eq. 4 and 5 are $k_4 = 8.5 \times 10^{6}$ $M^{-1}s^{-1}$, $k_5 = 4.2 \times 10^{8}$ $M^{1}s^{-1}$, respectively.[17] HE has been reported to react with $^{\bullet}OH$ with a second-order rate constant of $(7 \pm 1) \times 10^{9}$ $M^{-1}s^{-1}$.[2] Similar rate constant can be assumed for HPr^{+} probe. At pH 10.0, the concentrations of bicarbonate (HCO_3^{-}) and carbonate (CO_3^{2-}) in the 0.25 M bicarbonate solution are 0.17 and 0.08 M, respectively (pK_a of HCO_3^{-} is equal to 10.33).[18] Thus, the chosen

experimental conditions ensure that practically all $HO^\bullet$ radicals produced reacted directly with CO_3^{2-}.

Generation of nitrogen dioxide radical ($^\bullet NO_2$).

For the reaction of HPr^+ and HE with $^\bullet NO_2$, the solvated electron was converted into $^\bullet NO_2$ in the reaction with 0.1 M NO_3^- in the presence of 1 M *t*-butyl alcohol, used as a scavenger of $HO^\bullet$ radical. In this experiment, NO_3^- was reduced by e_{aq}^- with subsequent formation of the nitrate radical dianion ($NO_3^{\bullet 2-}$) ($k_6 = 1.0 \times 10^{10}$ M^{-1}s^{-1}). In the presence of sodium phosphate buffer (50 mM) both reaction 7 (Eq. 7, $k_7 = 1 \times 10^5$ M^{-1}s^{-1}) and reaction 8 (Eq. 8, $k_8 = 5 \times 10^8$ M^{-1}s^{-1}) contribute to the production of $^\bullet NO_2$.[19,20]

$$e_{aq}^- + NO_3^- \rightarrow NO_3^{\bullet 2-} \qquad \text{(Equation 6)}$$

$$NO_3^{\bullet 2-} + H_2O \rightarrow {}^\bullet NO_2 + 2OH^- \qquad \text{(Equation 7)}$$

$$NO_3^{\bullet 2-} + H_2PO_4^- \rightarrow {}^\bullet NO_2 + OH^- + HPO_4^{2-} \qquad \text{(Equation 8)}$$

Generation of thiyl radicals ($GS^\bullet$ or $CysS^\bullet$).

To study the reactivity of glutathione- and cysteine-derived thiyl radicals toward HPr^+ and HE, $GS^\bullet$ and $CysS^\bullet$ were generated by pulse radiolysis of N_2O-saturated aqueous solution of 2.5 mM glutathione (GSH) or cysteine (CysSH) and 1.6 M methanol (CH_3OH) in the presence of a phosphate buffer (50 mM, pH 7.4) *via* reactions described by Eqs. 9 – 12 ($k_9 = 2.3 \times 10^{10}$ M^{-1}s^{-1},[21] $k_{10} = 7.5 \times 10^8$ M^{-1}s^{-1},[11] $k_{11/12} \sim 1 \times 10^8$ M^{-1}s^{-1}),[22] where reactions described by Eq. 10 – 12 are favored over the reaction described by Eq. 9 under the experimental conditions.

$$HO^\bullet + GSH \rightarrow H_2O + GS^\bullet \qquad \text{(Equation 9)}$$

$$HO^{\bullet} + CH_3OH \rightarrow H_2O + {}^{\bullet}CH_2OH \qquad \text{(Equation 10)}$$

$${}^{\bullet}CH_2OH + GSH \rightarrow CH_3OH + GS^{\bullet} \qquad \text{(Equation 11)}$$

$${}^{\bullet}CH_2OH + CysSH \rightarrow CH_3OH + CysS^{\bullet} \qquad \text{(Equation 12)}$$

Generation of chloromethylperoxyl radicals.

To investigate the reactivity of HE toward chloromethylperoxyl ($CH_2ClO_2^{\bullet}$), dichloromethylperoxyl ($CHCl_2O_2^{\bullet}$), and trichloromethylperoxyl ($CCl_3O_2^{\bullet}$) radicals, we used O_2-saturated 2-propanol:water (1:1, v/v) mixture containing 4% (v/v) of appropriate halocarbon compound and 20 mM phosphate buffer (pH 7.4). In the first step, alkyl radicals were produced by the reaction of e_{aq}^{-} with the halogenated organic compound (CH_xCl_y), leading to reductive elimination of the halide ion (Eq. 13, $k_{13} \sim 10^9$–10^{10} $M^{-1}s^{-1}$).[23-27]

$$e_{aq}^{-} + CH_xCl_y \rightarrow Cl^{-} + {}^{\bullet}CH_xCl_{y-1} \qquad \text{(Equation 13)}$$

The hydroxyl radical was scavenged by 2-propanol (Eq. 14, $k_{14} = 1.9 \times 10^9$ $M^{-1}s^{-1}$).[11] The resulting ketyl radical reacts further with halogenated compounds to produce additional quantity of the desired alkyl radicals (Eq. 15, $k_{15} = 10^6$ - 10^8 $M^{-1}s^{-1}$) [28-30].

$$HO^{\bullet} + (\mathrm{CH_3})_2\mathrm{CHOH} \rightarrow \mathrm{H_2O} + (\mathrm{CH_3})_2\mathrm{C^{\bullet}OH} \qquad \text{(Equation 14)}$$

$$(\mathrm{CH_3})_2\mathrm{C^{\bullet}OH} + \mathrm{CH}_x\mathrm{Cl}_y \rightarrow (\mathrm{CH_3})_2\mathrm{CO} + \mathrm{H^+} + \mathrm{Cl^-} + {}^{\bullet}CH_xCl_{y-1} \qquad \text{(Equation 15)}$$

The halogenated alkyl radicals react rapidly with oxygen to form peroxyl radicals (Eq. 16 $k_{16} > 10^9$ $M^{-1}s^{-1}$).[31-33]

$${}^{\bullet}CH_xCl_{y-1} + \mathrm{O_2} \rightarrow CH_xCl_{y-1}\mathrm{OO^{\bullet}} \qquad \text{(Equation 16)}$$

Generation of superoxide radical anion ($O_2^{\bullet-}$) and its reaction with $HPr^{\bullet 2+}$ and $TMeHPr^{\bullet 2+}$.

Superoxide radical anion was generated in the aqueous solution of sodium formate (50 mM) in 5 mM phosphate buffer (pH 7.4,) in the presence of 3 mM sodium azide. Additionally, prior to pulse radiolysis, the solution was saturated with oxygen. Under these conditions, the generated hydroxyl radical reacts subsequently with the azide yielding $N_3^{\bullet}$ (Eq. 2) and formate yielding carbon dioxide radical anion ($CO_2^{\bullet-}$) (Eq. 17, k_{17} = 3.2 × 10^9 M^{-1}s^{-1})[11] In this reaction mixture superoxide is formed *via* the reduction of dissolved oxygen by $CO_2^{\bullet-}$ (Eq. 18, k_{18} = 2.4 × 10^9 M^{-1}s^{-1})[34] as well as in the quantitative reaction of other transients formed upon irradiation, like e_{aq}^- (Eq. 19, k_{19} = 1.9 × 10^{10} M^{-1}s^{-1})[35] and hydrogen atom ($H^{\bullet}$)(Eq. 20, k_{20} = 1.2 × 10^{10} M^{-1}s^{-1}),[11] with molecular oxygen. When HPr^+ or $TMeHPr^+$ was present in the solution, the formation of their radical cations due to the reaction with $N_3^{\bullet}$ was observed.

$$HCO_2^- + HO^{\bullet} \rightarrow CO_2^{\bullet -} + H_2O \qquad \text{(Equation 17)}$$

$$CO_2^{\bullet -} + O_2 \rightarrow CO_2 + O_2^{\bullet -} \qquad \text{(Equation 18)}$$

$$e_{aq}^- + O_2 \rightarrow O_2^{\bullet -} \qquad \text{(Equation 19)}$$

$$H^{\bullet} + O_2 \rightarrow H^+ + O_2^{\bullet -} \qquad \text{(Equation 20)}$$

The initial concentrations of radical cations of $TMeHPr^+$ and HPr^+ were calculated using their molar extinction coefficients (Table S4) and their initial absorptions determined at 480 nm for $TMeHPr^{\bullet 2+}$ and 460 nm for $HPr^{\bullet 2+}$. Assuming that all transients formed upon irradiation of oxygenated solution of formate were quantitatively transformed to superoxide radical anion, using the total radical yield (0.62 µM/Gy) the maximal concentration of $O_2^{\bullet-}$ could be calculated. In the presence of azide ions part of the radical transients were quantitatively transformed to azide radicals and subsequently to the radical cations of $TMeHPr^+$ or HPr^+. Thus, the actual initial

concentration of $O_2^{\bullet-}$ was calculated by subtracting the initial concentration of $TMeHPr^{\bullet 2+}$ or $HPr^{\bullet 2+}$ from the maximal concentration of $O_2^{\bullet-}$ calculated on the basis of total radical yield. Under the experimental conditions, the calculated initial concentration of superoxide radical anion was at least five times higher than the initial concentration of $TMeHPr^+$ or HPr^+ radical cations and the kinetics of the reaction fitted the pseudo-first decay of both species. The observed rate constants of the reaction between $O_2^{\bullet-}$ and the studied probes were determined by fitting single exponential curves to the kinetic traces of $TMeHPr^{\bullet 2+}$ or $HPr^{\bullet 2+}$ decay.

Kinetic simulations.

The values of the rate constants of HPr^+ reaction with radiolytically generated one-electron oxidants were also determined by kinetic simulations. In computational calculations, we included self-decay of radical oxidant and dismutation of hydropropidine radical cation. We also included the reaction of the oxidant with hydropropidine radical cation.

We were changing the values of the rate constants of the oxidant reaction with HPr^+ and with $HPr^{\bullet 2+}$ to find the best fit of the computed values of $HPr^{\bullet 2+}$ build up to the experimental values. The resulting values of second order rate constant for the reaction of HPr^+ with one-electron oxidants are presented below.

$N_3^\bullet$ radical

**Reaction**	***k*** **($M^{-1}s^{-1}$)**	**ref**
$HPr^+ + N_3^\bullet \rightarrow HPr^{\bullet 2+} + N_3^-$	4.8×10^9	simulations
$N_3^\bullet + N_3^\bullet \rightarrow$ products	4.4×10^9	Ref.[36]
$2\ HPr^{\bullet 2+} \rightarrow HPr^+ + Pr^{2+}$	3.3×10^8	This work
$HPr^{\bullet 2+} + N_3^\bullet \rightarrow Pr^{2+} + N_3^-$	1.0×10^9	simulations

**HPr^+ (μM)**	**k_{exp} (s^{-1})**	**k_{sim} (s^{-1})**
24	1.6×10^5	1.7×10^5
40	2.4×10^5	2.3×10^5
62	3.3×10^5	3.3×10^5
79	4.3×10^5	4.2×10^5

$^{\bullet}NO_2$ radical

Reaction	*k* ($M^{-1}s^{-1}$)	ref
$HPr^{+} + {}^{\bullet}NO_2 \rightarrow HPr^{\bullet 2+} + NO_2^{-}$	6.1×10^{8}	simulations
${}^{\bullet}NO_2 + {}^{\bullet}NO_2 \rightarrow$ products	4.6×10^{8}	Ref.[13]
$2\ HPr^{\bullet 2+} \rightarrow HPr^{+} + Pr^{2+}$	3.3×10^{8}	This work
$HPr^{\bullet 2+} + N_3^{\bullet} \rightarrow Pr^{2+} + N_3^{-}$	6.1×10^{8}	simulations

HPr^{+} (μM)	k_{exp} (s^{-1})	k_{sim} (s^{-1})
20	2.0×10^{4}	2.1×10^{4}
35	2.8×10^{4}	2.9×10^{4}
50	3.9×10^{4}	3.9×10^{4}
70	5.3×10^{4}	5.2×10^{4}

$CO_3^{\bullet -}$ radical anion

Reaction	*k* ($M^{-1}s^{-1}$)	ref
$HPr^{+} + CO_3^{\bullet -} \rightarrow HPr^{\bullet 2+} + CO_3^{2-}$	5.3×10^{9}	simulations
$CO_3^{\bullet -} + CO_3^{\bullet -} \rightarrow$ products	1.4×10^{7}	Ref.[37]
$2\ HPr^{\bullet 2+} \rightarrow HPr^{+} + Pr^{2+}$	3.3×10^{8}	This work
$HPr^{\bullet 2+} + CO_3^{\bullet -} \rightarrow Pr^{2+} + CO_3^{2-}$	4.0×10^{9}	simulations

HPr^{+} (μM)	k_{exp} (s^{-1})	k_{sim} (s^{-1})
26	1.9×10^{5}	1.9×10^{5}
43	3.1×10^{5}	2.7×10^{5}
60	3.4×10^{5}	3.6×10^{5}
83	4.7×10^{5}	4.8×10^{5}

^{1}H NMR ($CDCl_3$) of compound **TMeE⁺**.

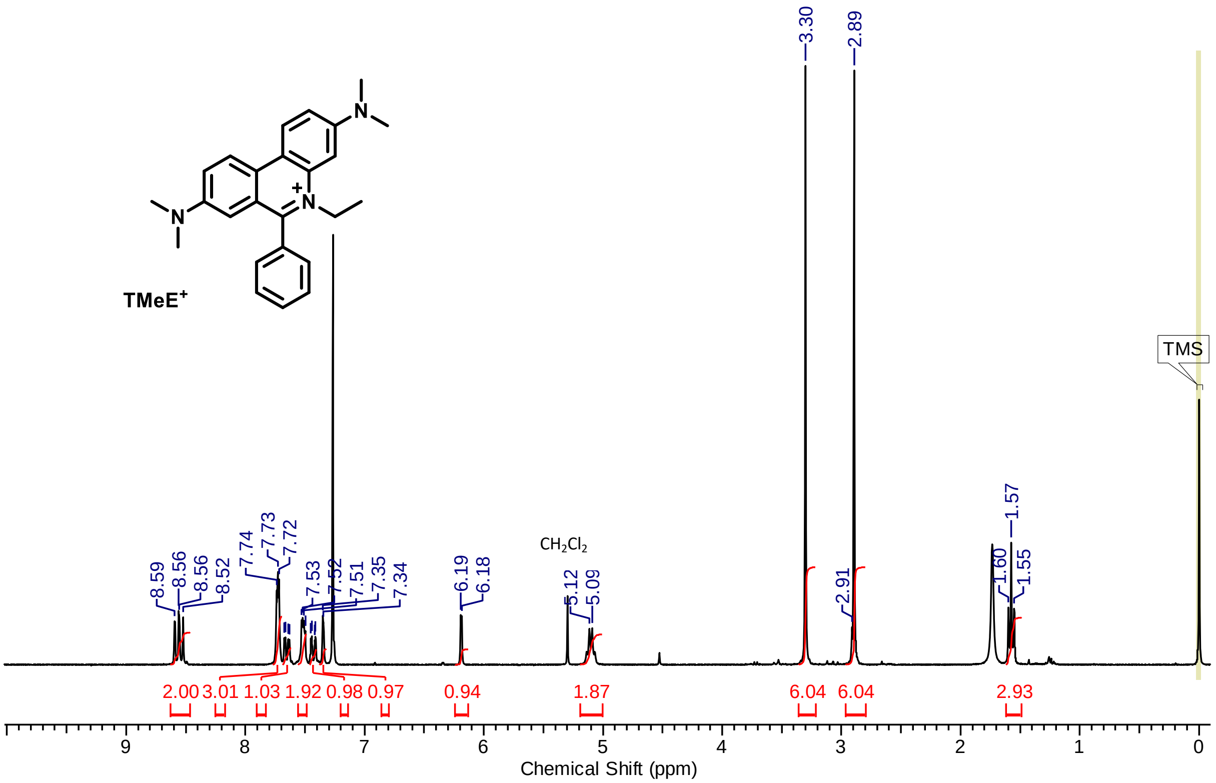

^{13}C NMR ($CDCl_3$) of compound **TMeE⁺**.

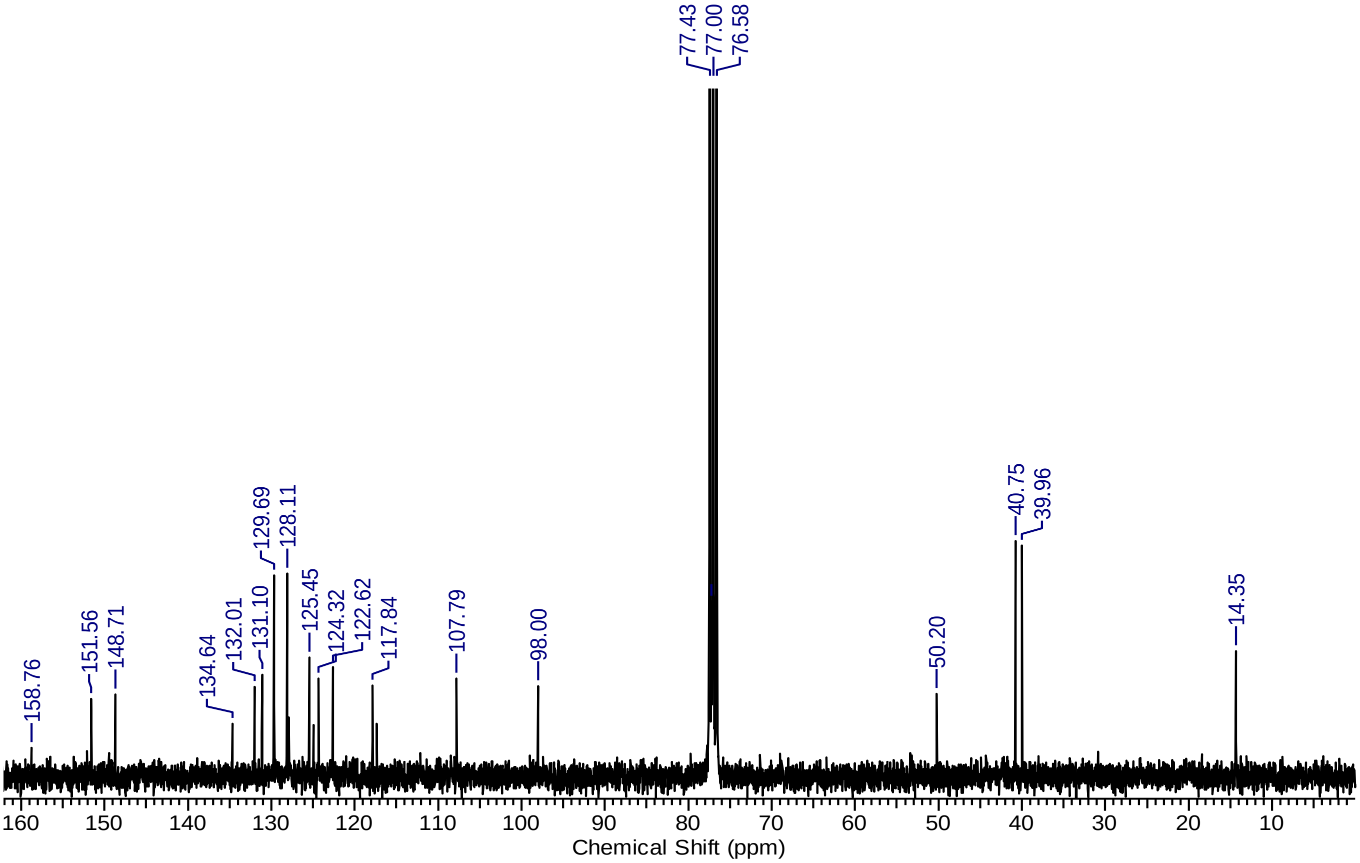

Geometry of **TMeE⁺** from X-ray diffraction analysis of **TMeE⁺•Cl⁻** crystals (Pov-Ray view).

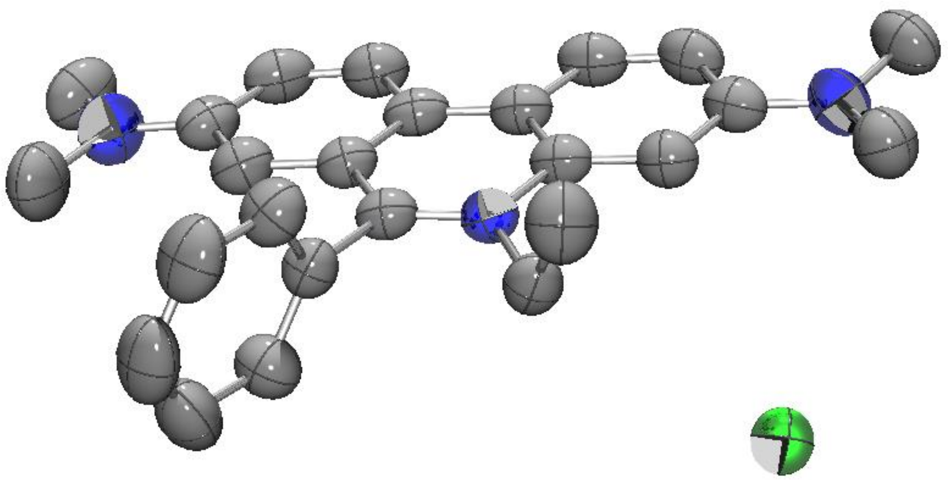

^1H NMR ($CDCl_3$) of compound **TMeHE**.

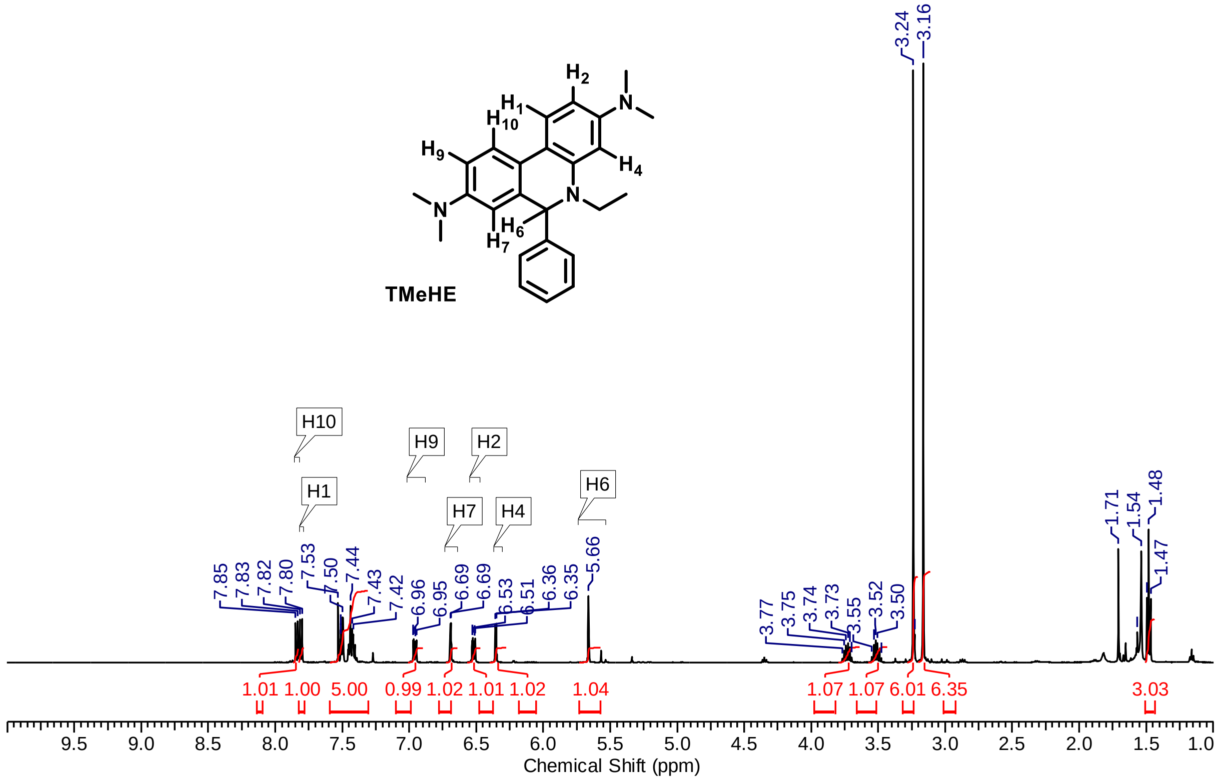

^{13}C NMR ($CDCl_3$) of compound **TMeHE**.

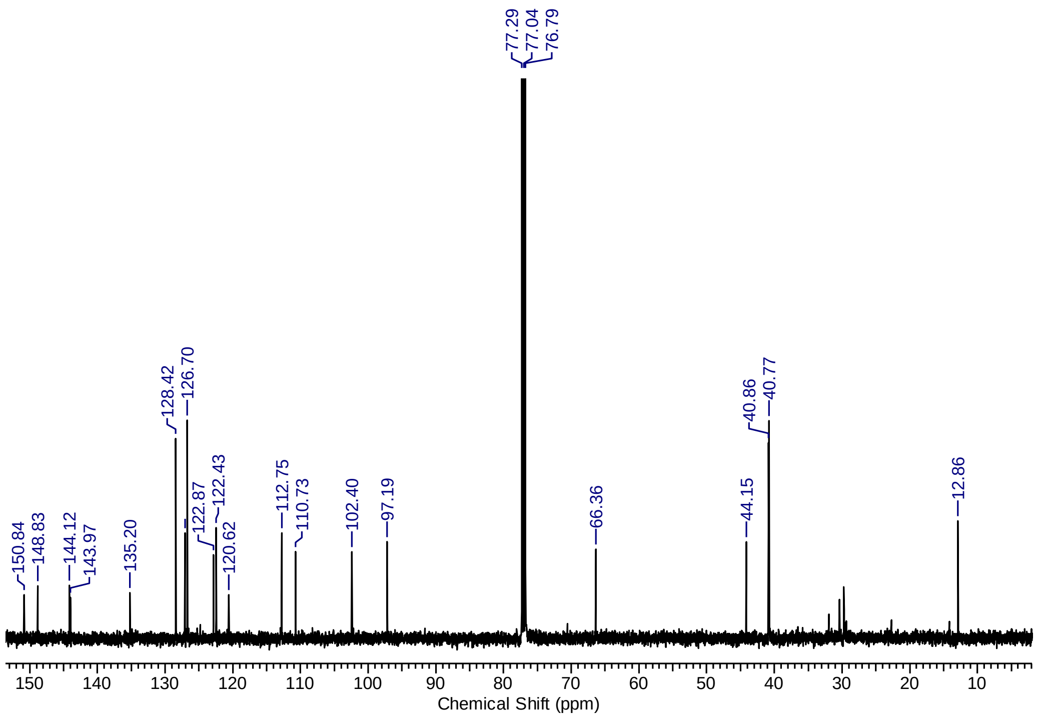

^{1}H NMR ($CDCl_3$) of **2-OH-TMeE^{+}**.

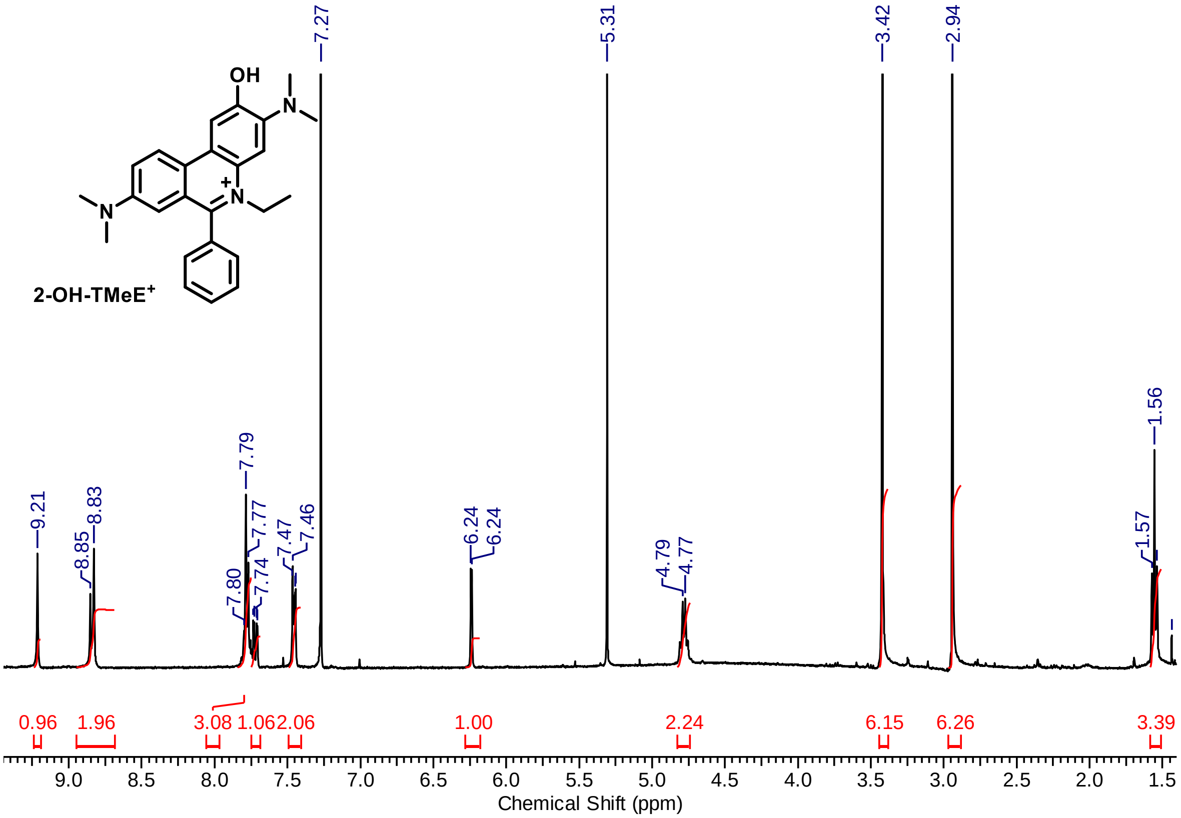

^{13}C NMR ($CDCl_3$) of compound **2-OH-TMeE^{+}**.

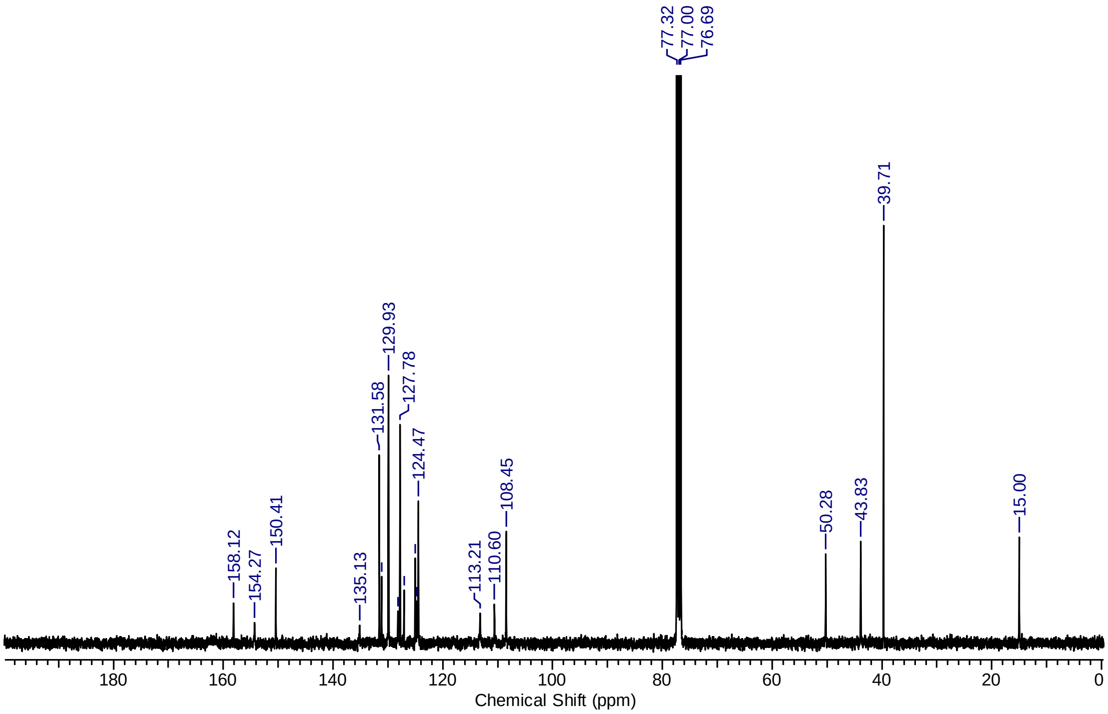

Geometry of **2-OH-TMeE** from X-ray diffraction analysis of **2-OH-TMeE⁺**•CF_3COO^- crystals (Pov-Ray view).

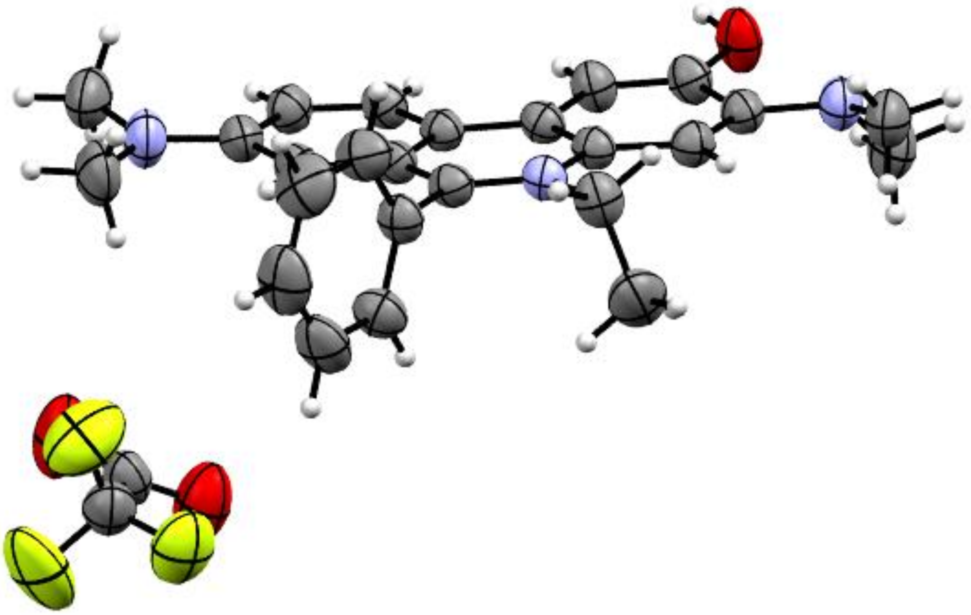

^{1}H NMR (CD_3OD) of compound **1**.

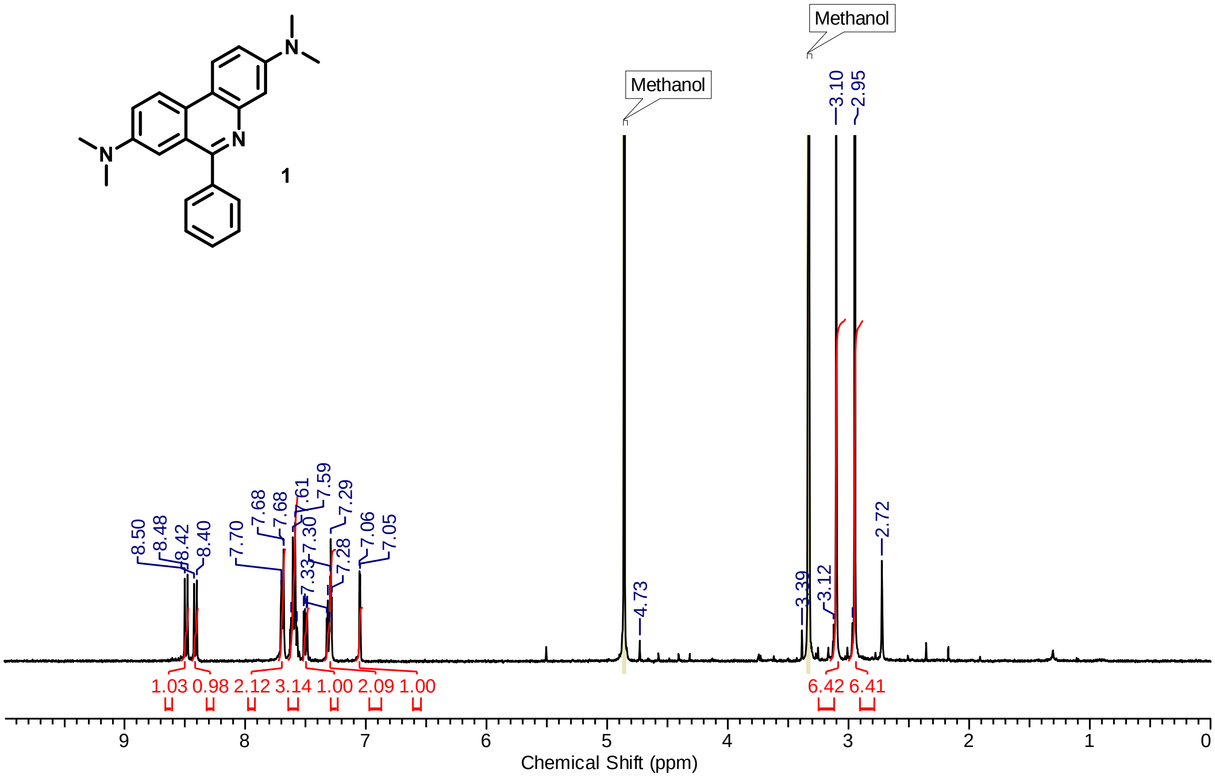

^{13}C NMR (CD_3OD) of compound **1**.

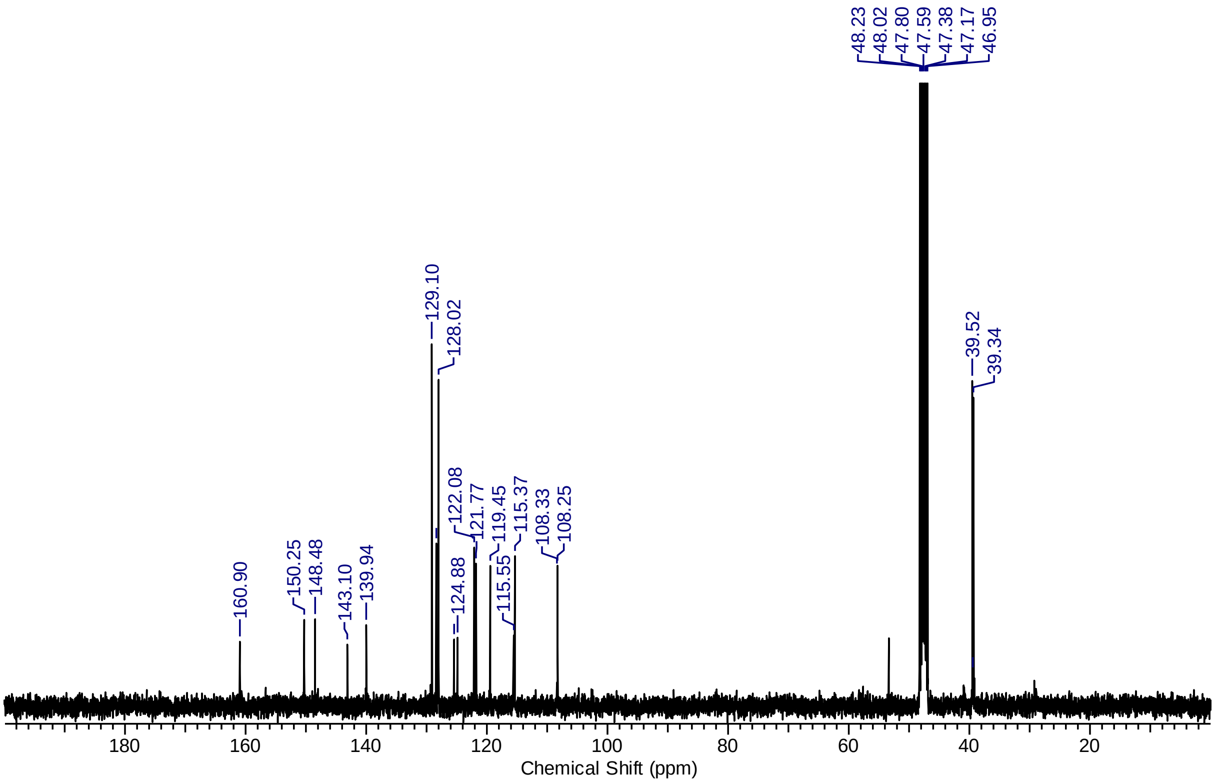

HPLC and ESI-MS analysis of compound **1**.

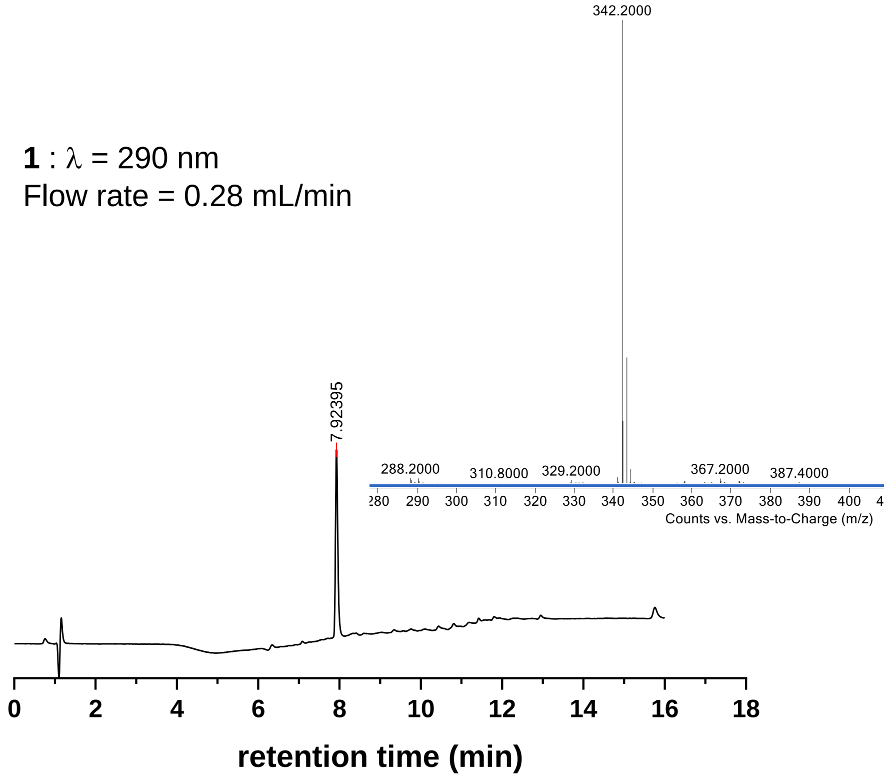

^1H NMR ($CDCl_3$) of 3-iodopropyl trifluoromethanesulfonate.

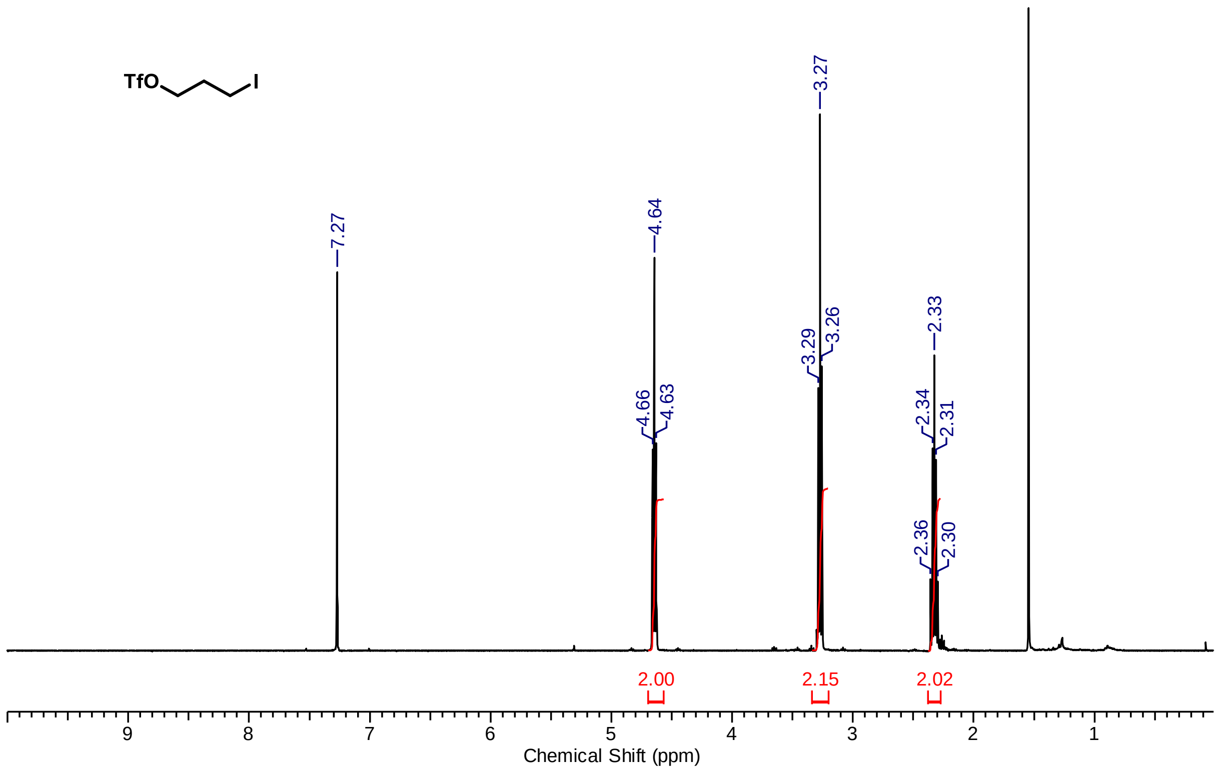

^{1}H NMR ($CDCl_3$) of compound **2**.

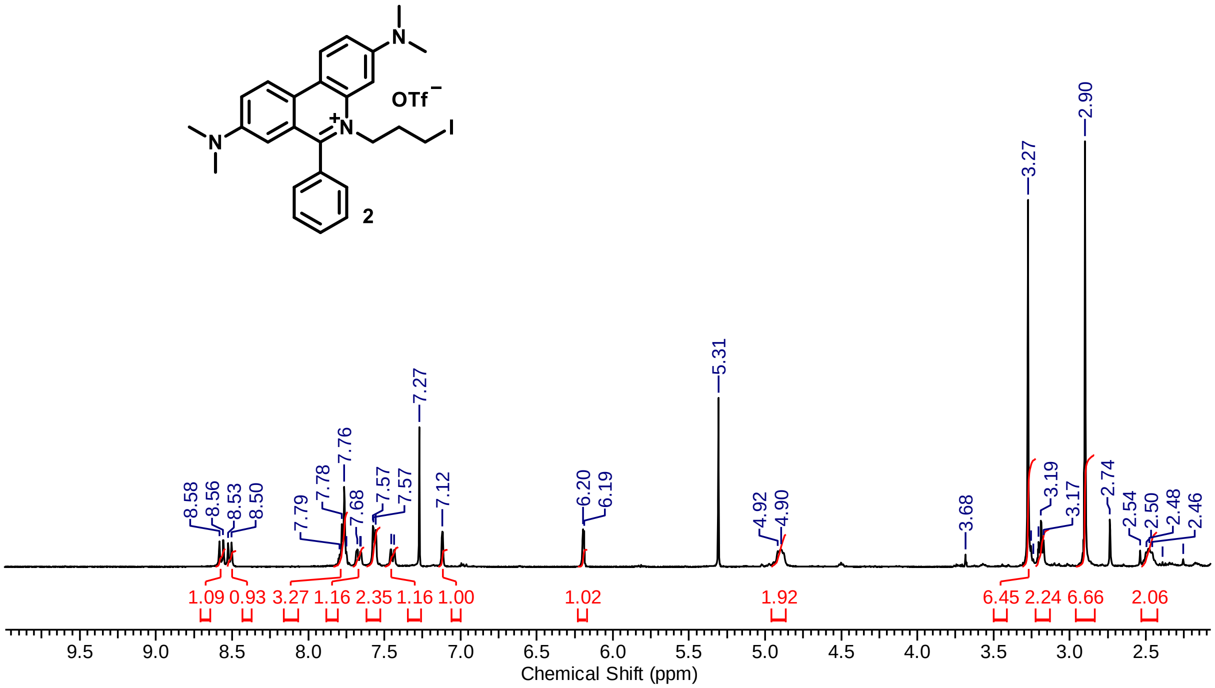

^{13}C NMR ($CDCl_3$) of compound **2**.

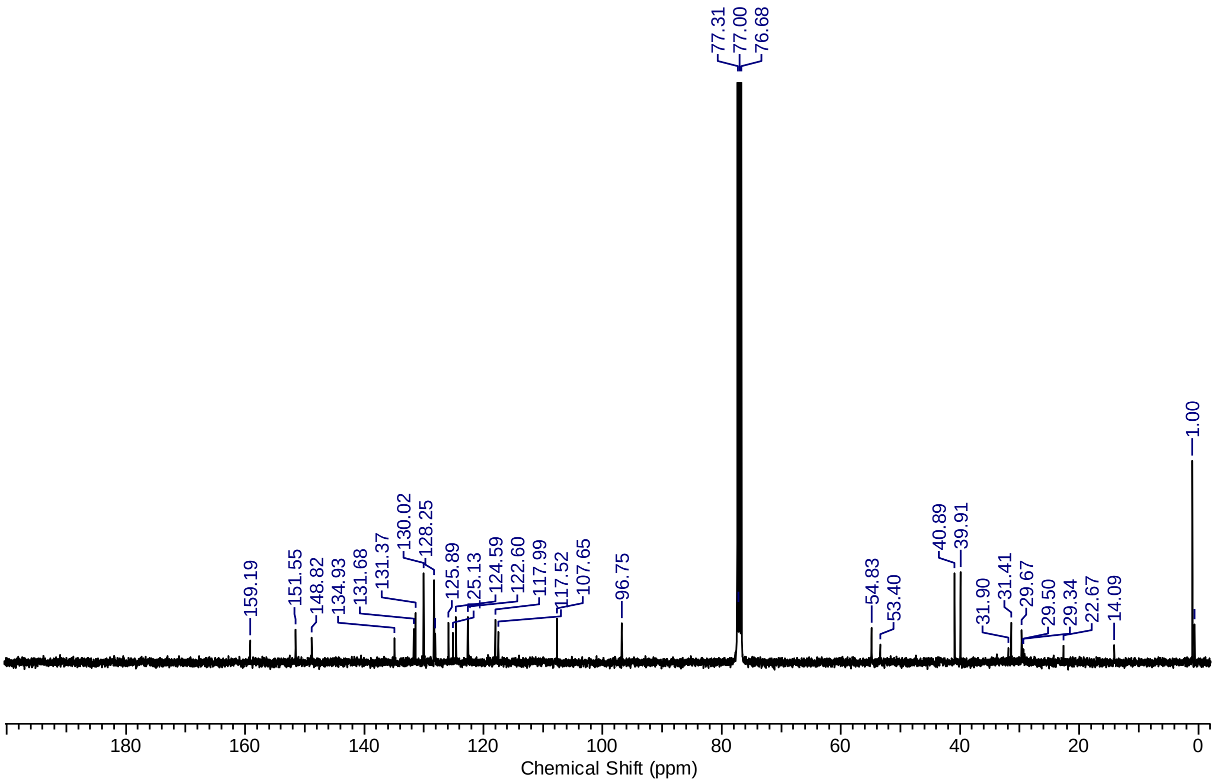

HPLC analysis of compound **2**.

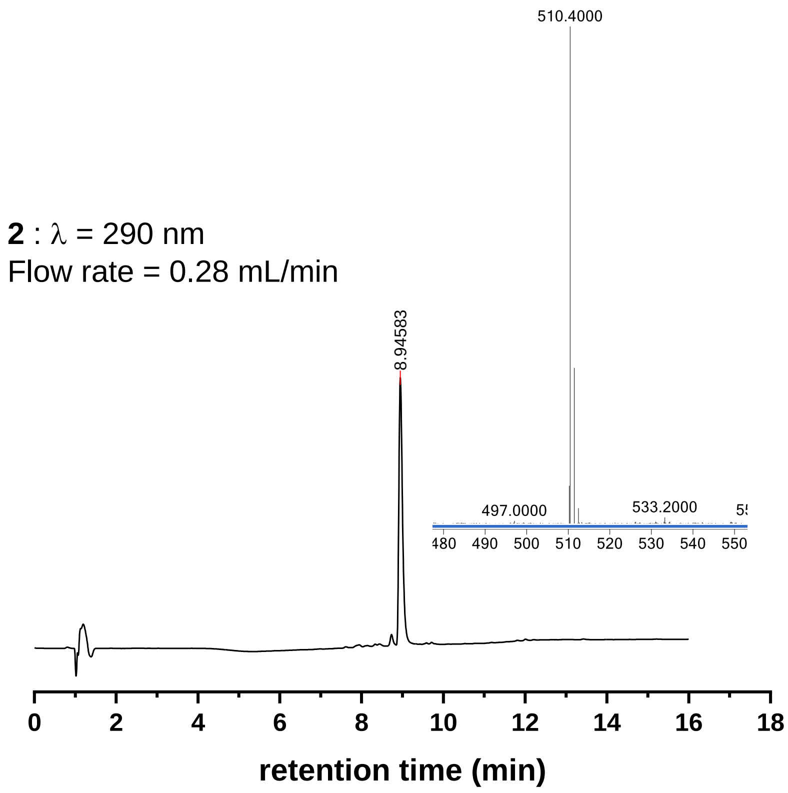

^{1}H NMR (CD_3OD) of **TMePr^{++}**.

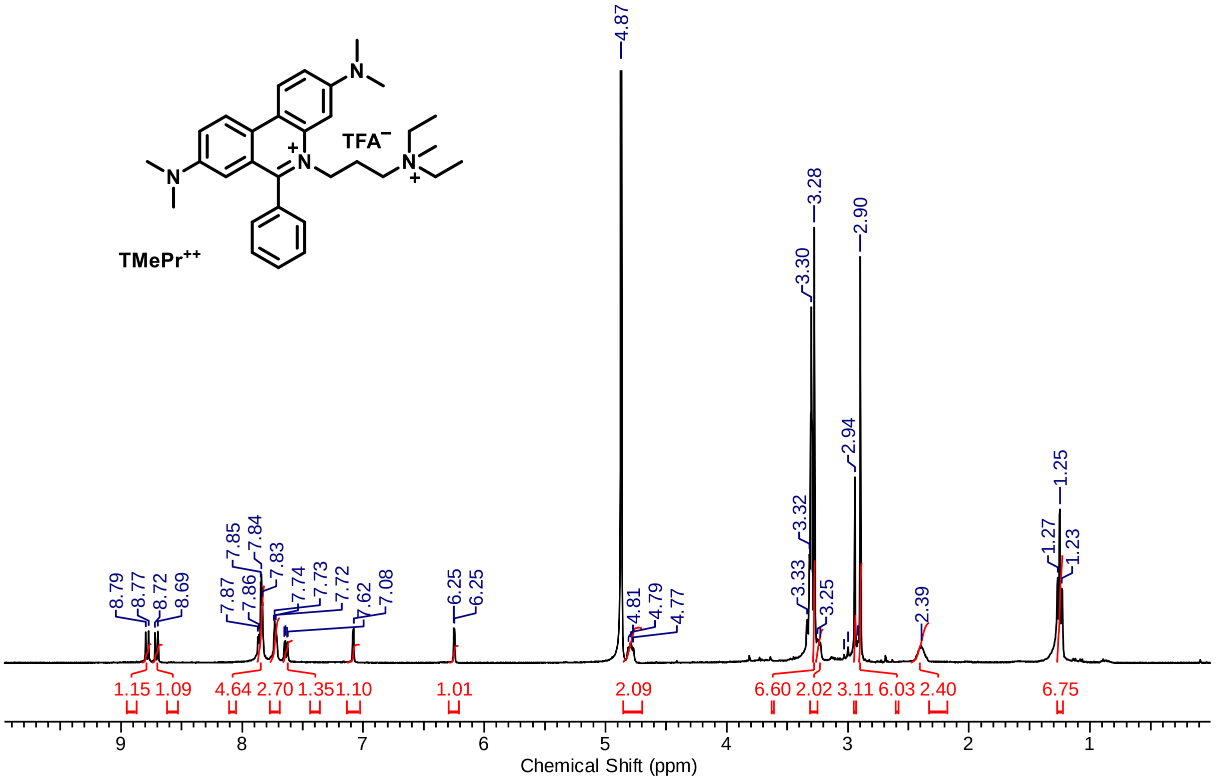

^{13}C NMR (CD_3OD) of **TMePr^{++}**.

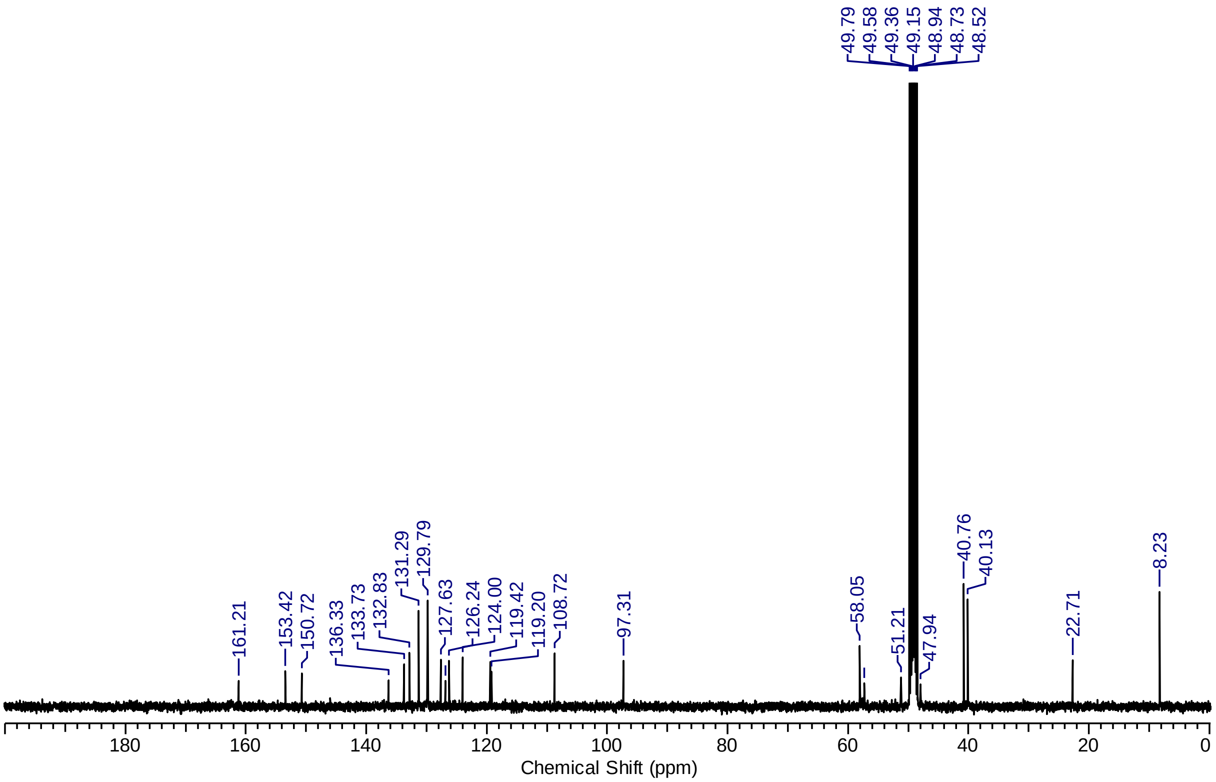

HPLC analysis of compound **TMePr⁺⁺** (λ= 290 nm).

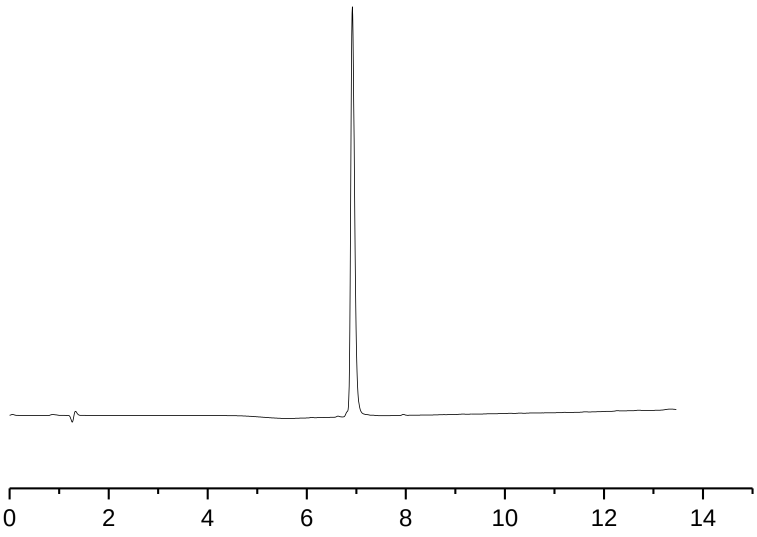

^{1}H NMR (D_2O) of **TMeHPr⁺**.

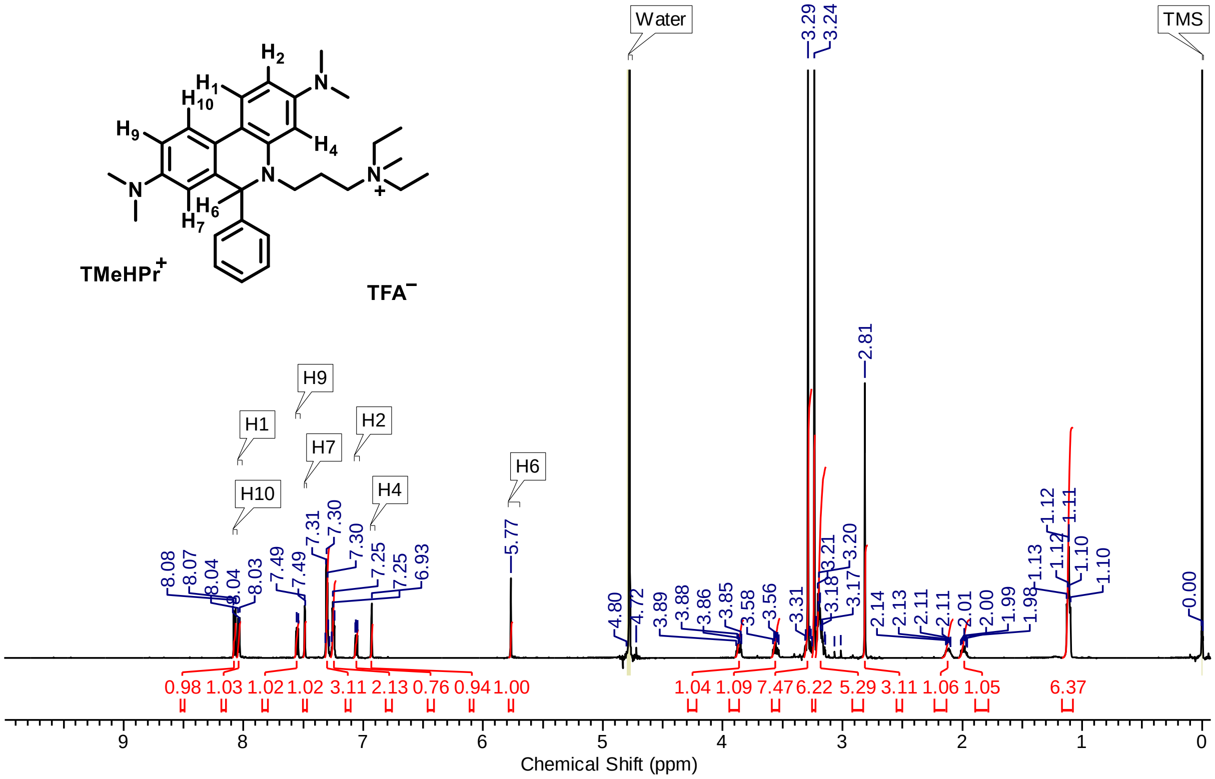

^{13}C NMR (D_2O) of **TMeHPr⁺**.

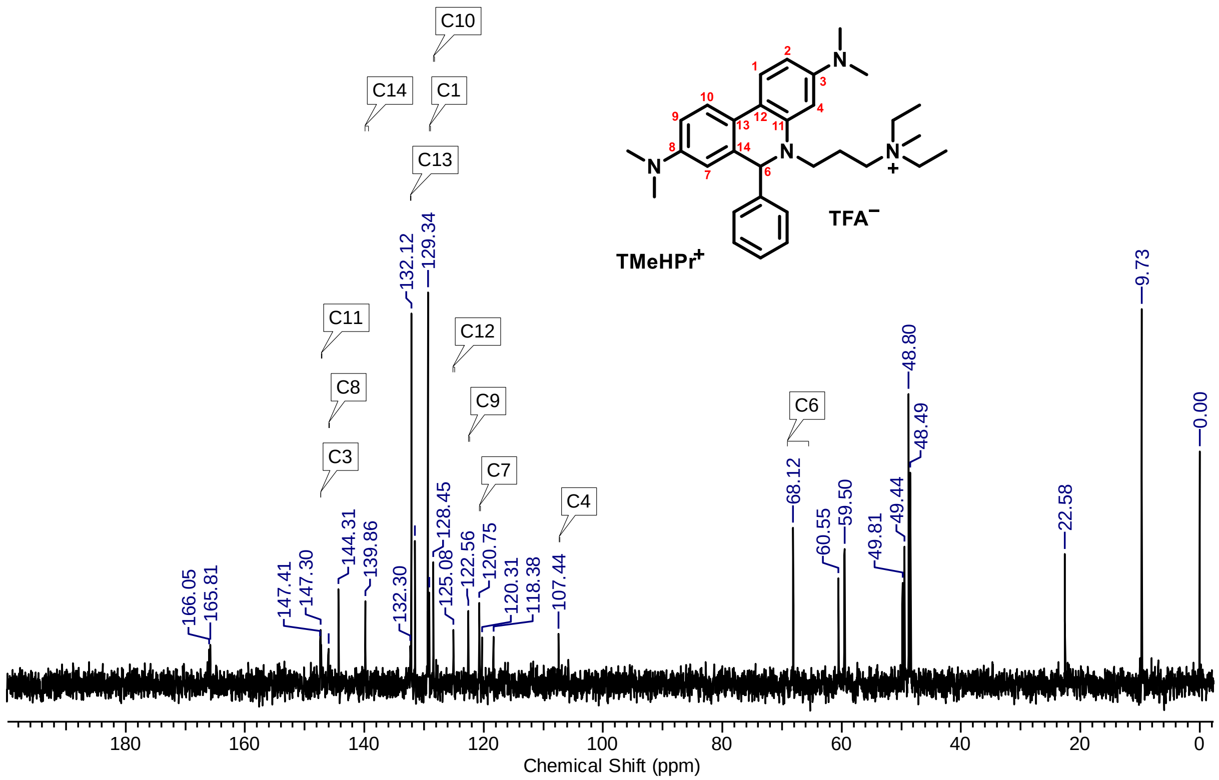

HSQC (D_2O) of **TMeHPr$^+$.**

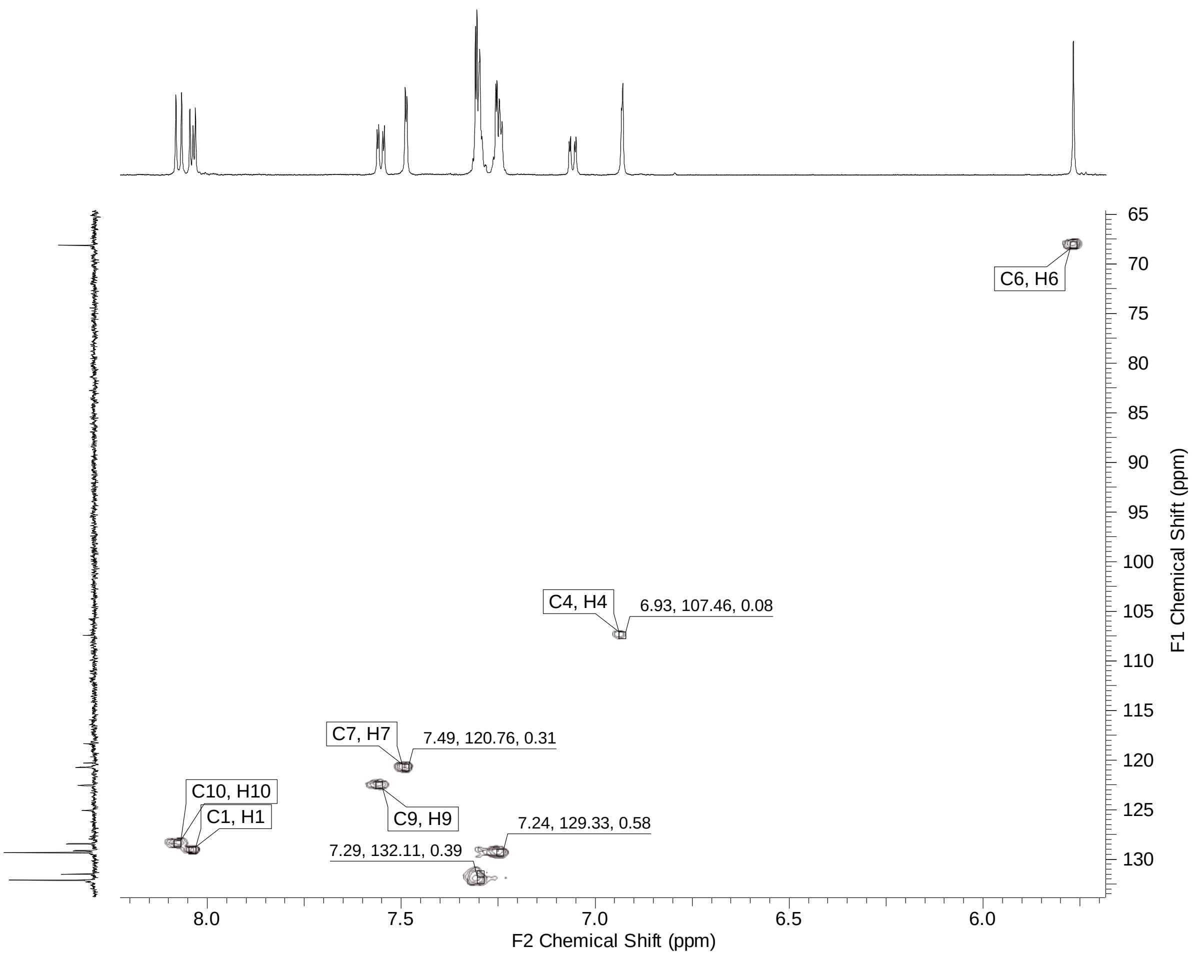

HMBC (D_2O) of **TMeHPr⁺**.

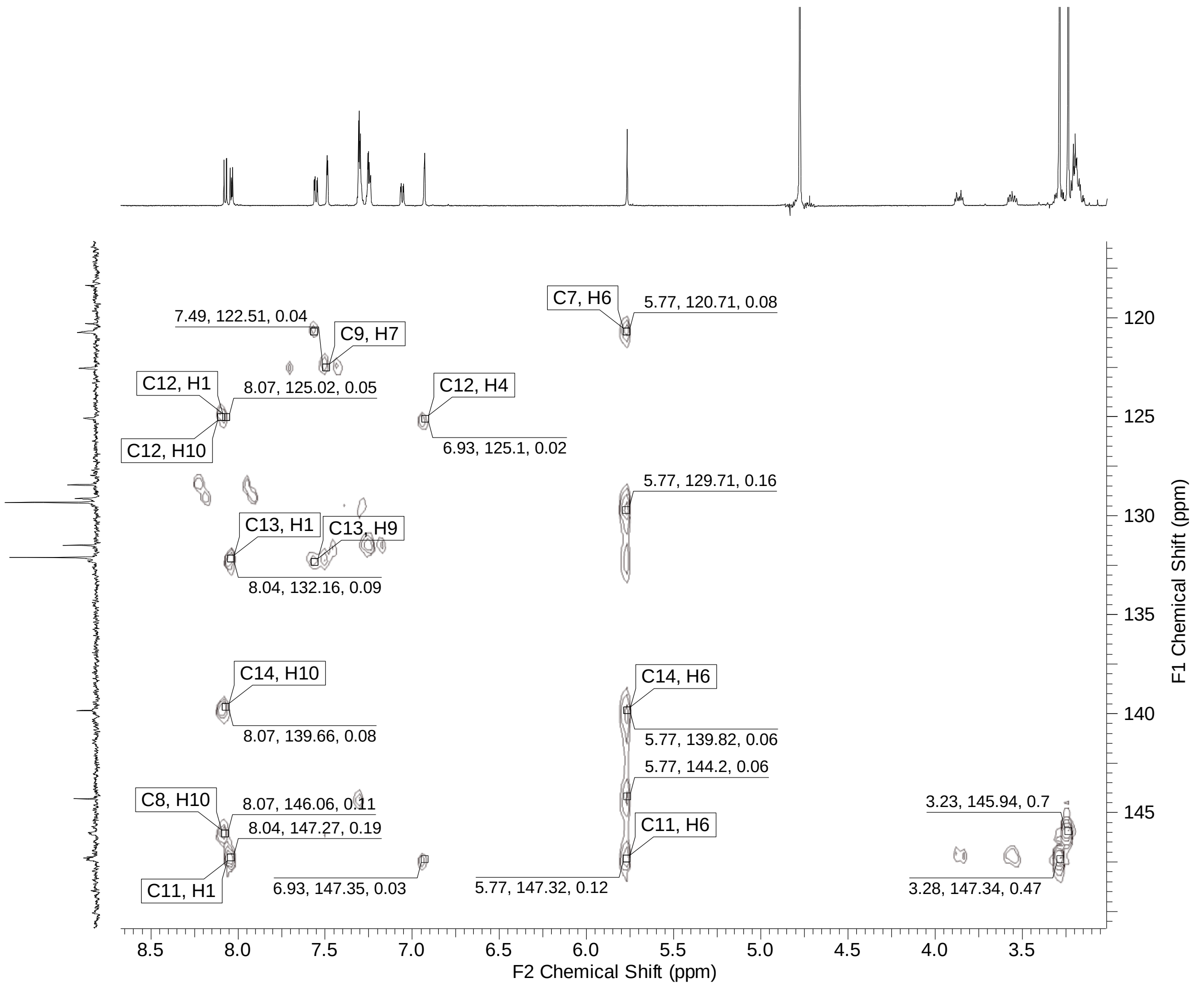

HPLC analysis of compound **TMeHPr⁺** (λ= 290 nm).

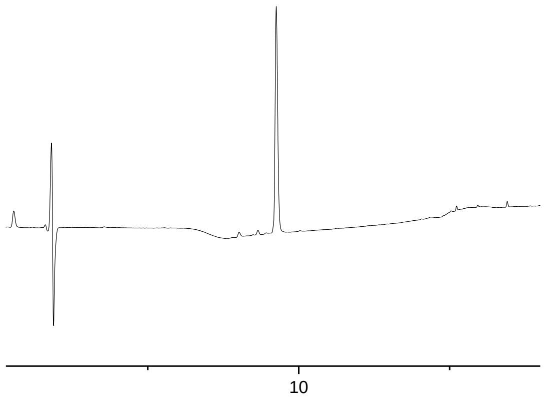

^{1}H NMR (CD_3OD) of **2-OH-TMePr^{++}**.

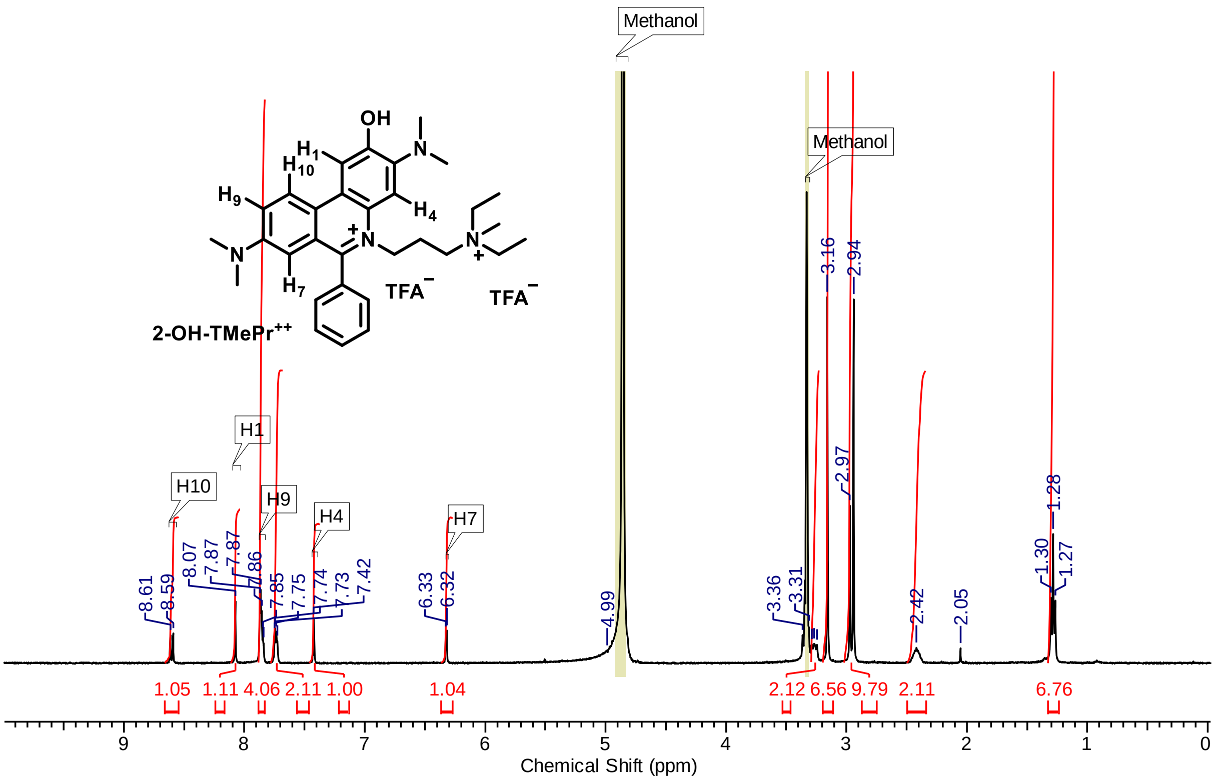

^{13}C NMR (CD_3OD) of **2-OH-TMePr^{++}**.

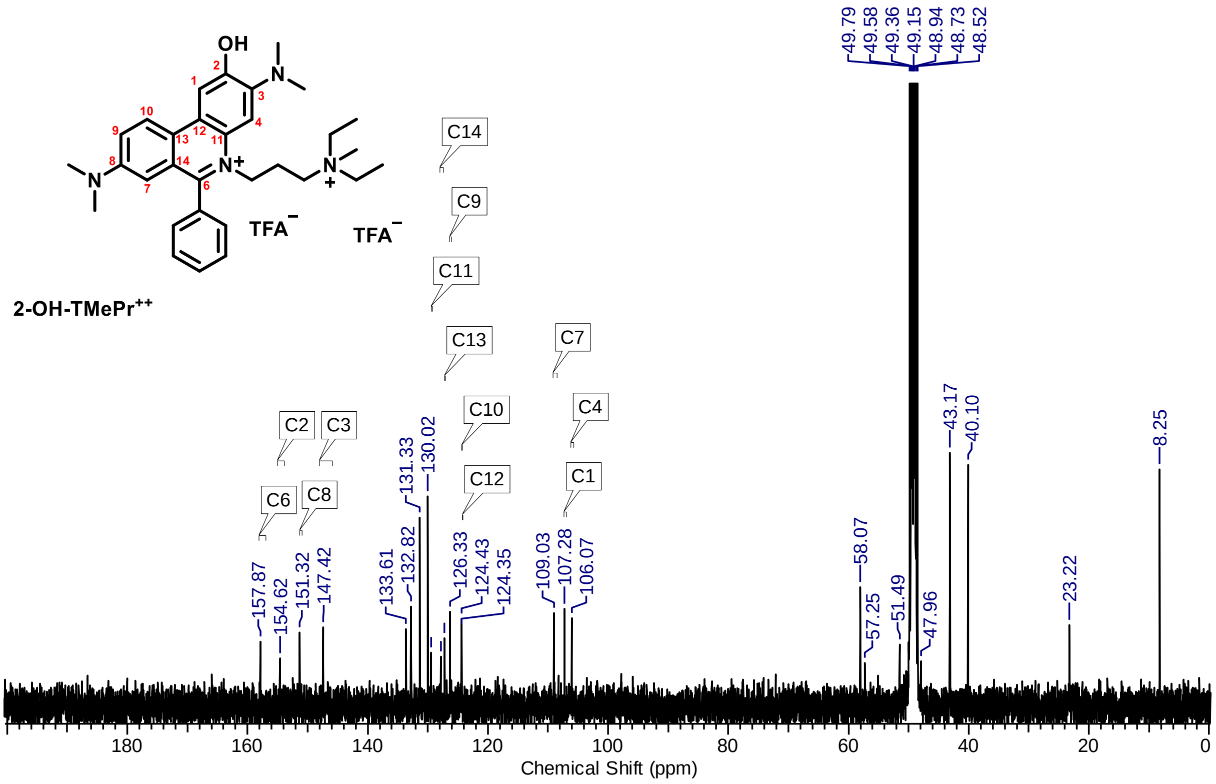

HSQC (CD_3OD) of **2-OH-TMePr++**.

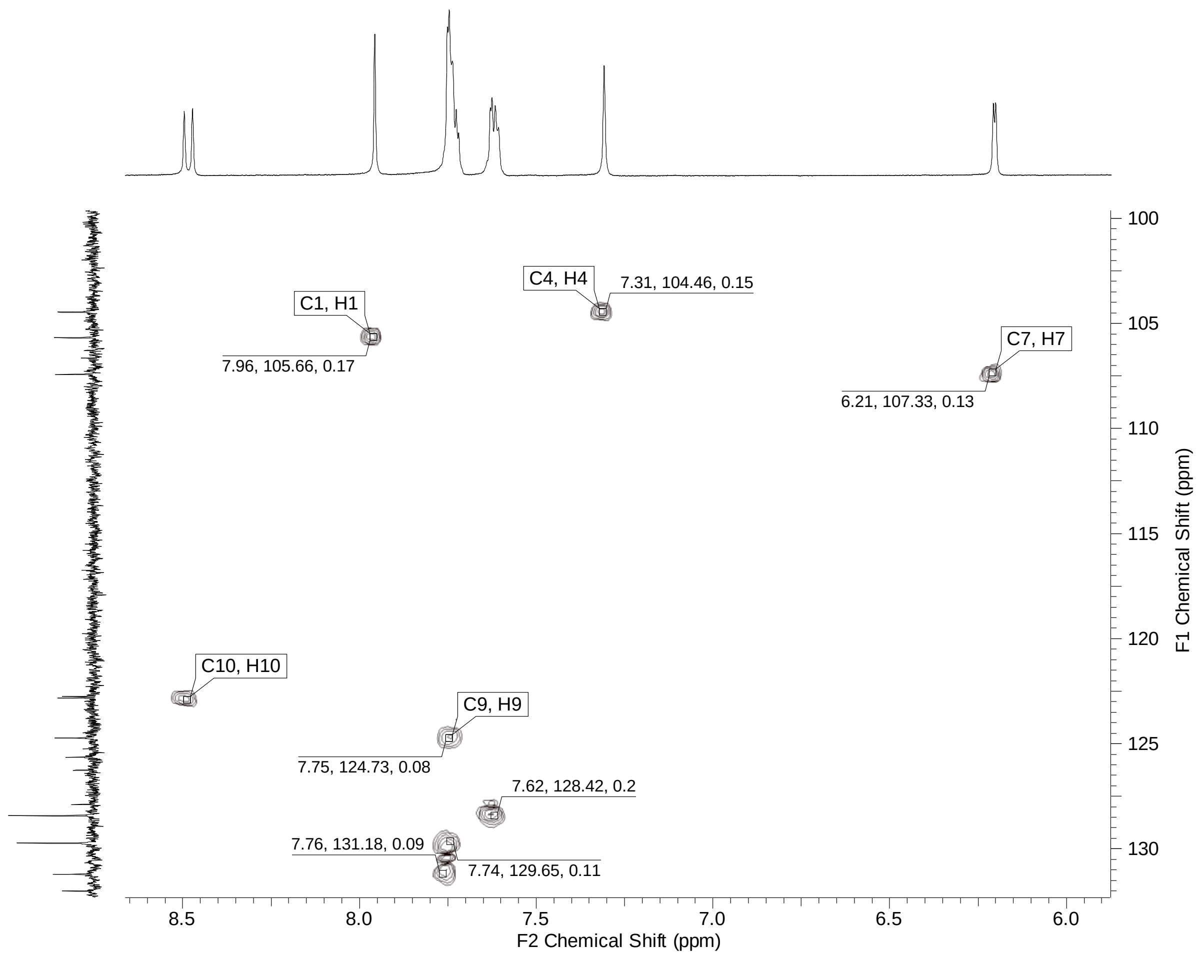

HMBC (CD_3OD) of **2-OH-TMePr^{++}**.

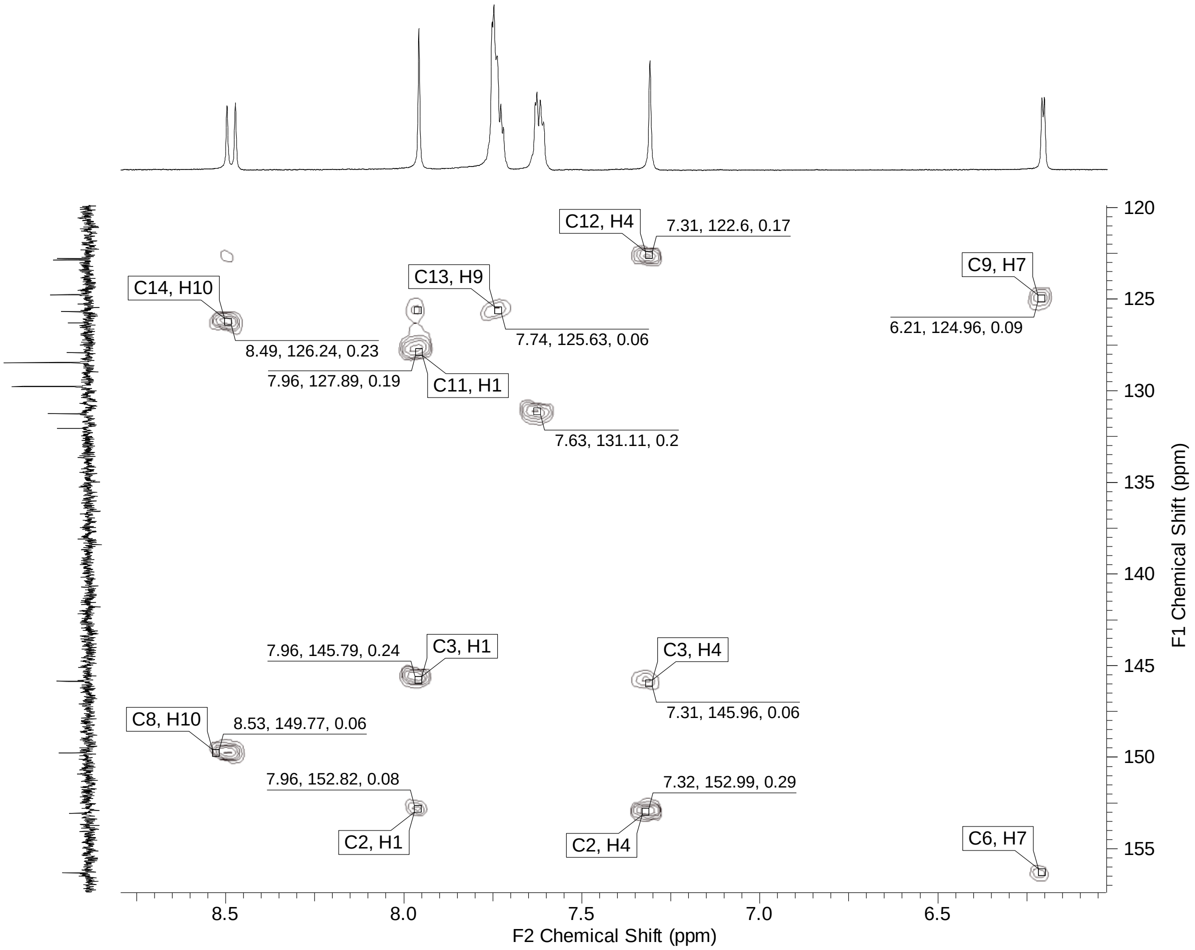

^{1}H NMR (DMSO d_6) of compound **3**.

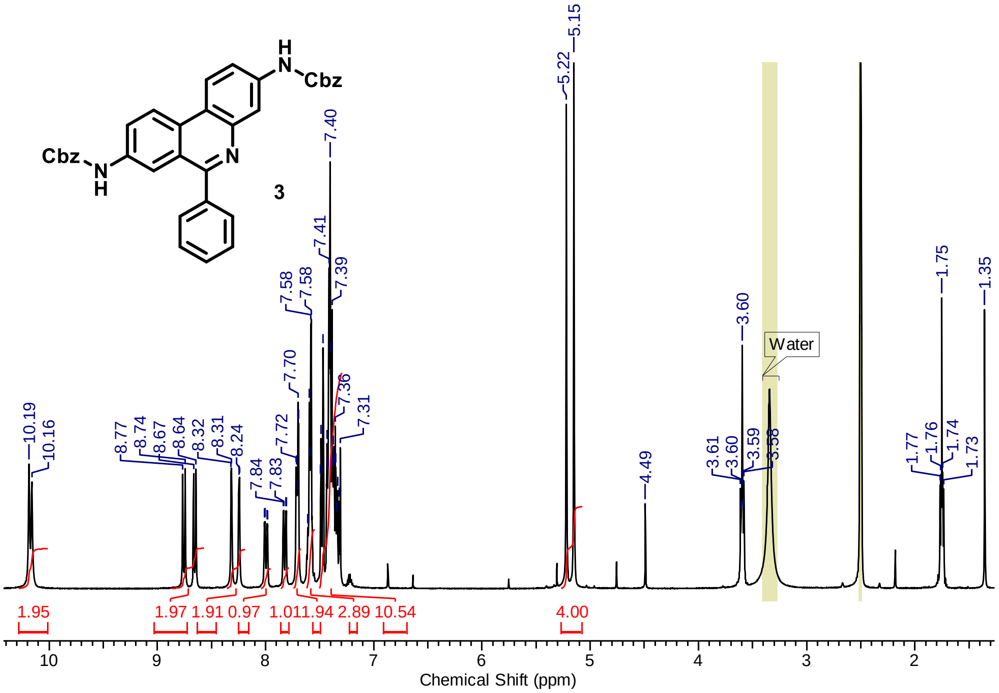

^{13}C NMR (DMSO d_6) of compound **3**.

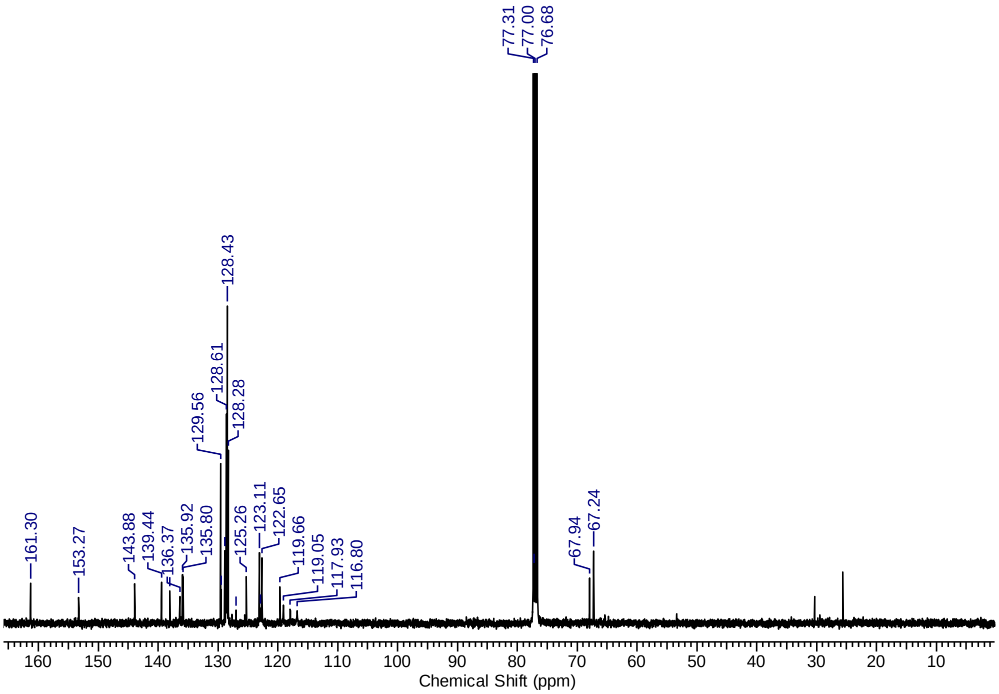

HPLC and ESI-MS of compound **3**.

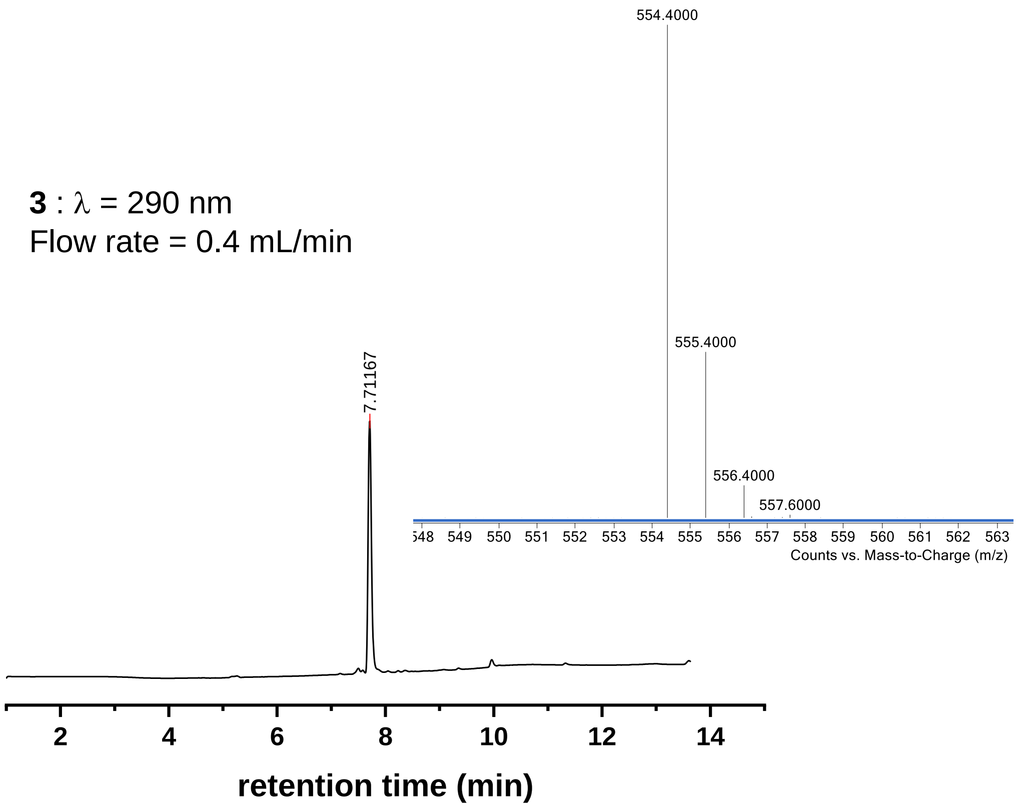

^{1}H NMR (CD_3OD) of compound **4**.

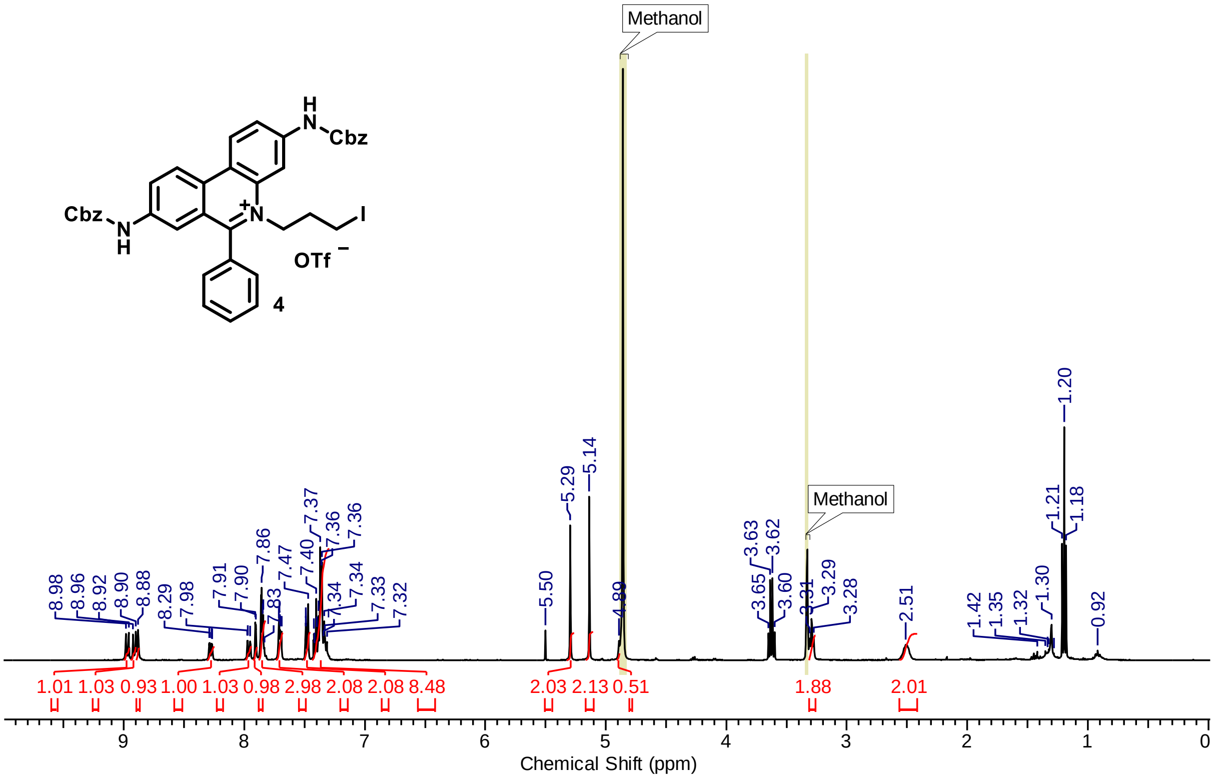

HPLC and ESI-MS of compound **4.**

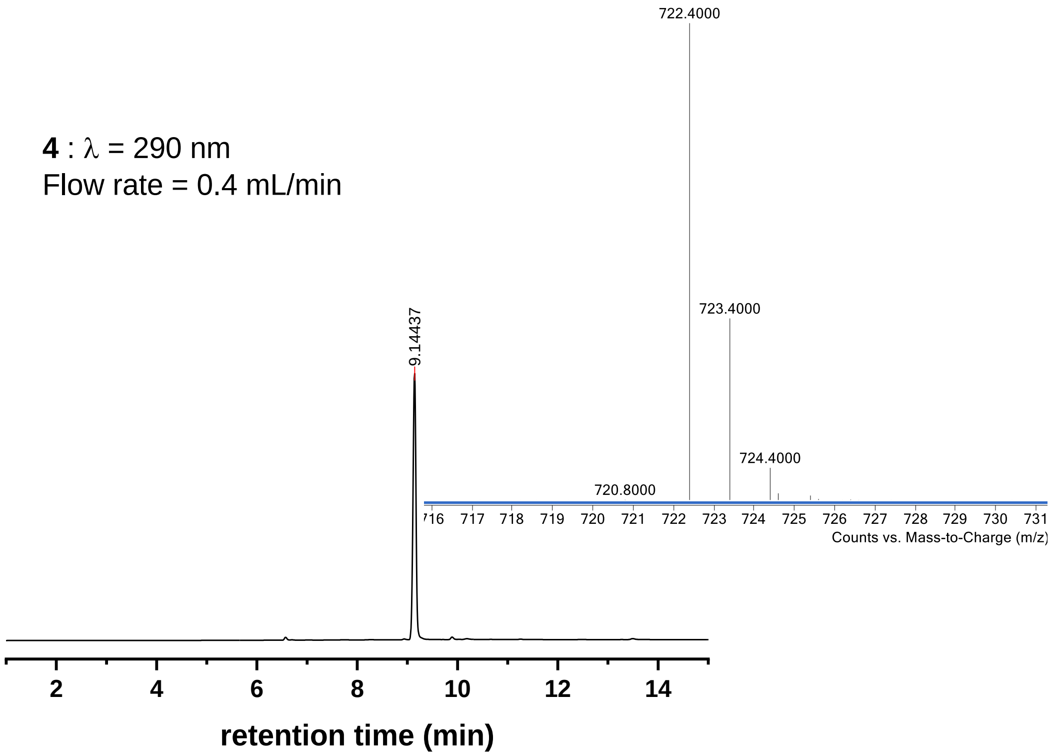

^{1}H NMR (CD_3OD) of compound **5**.

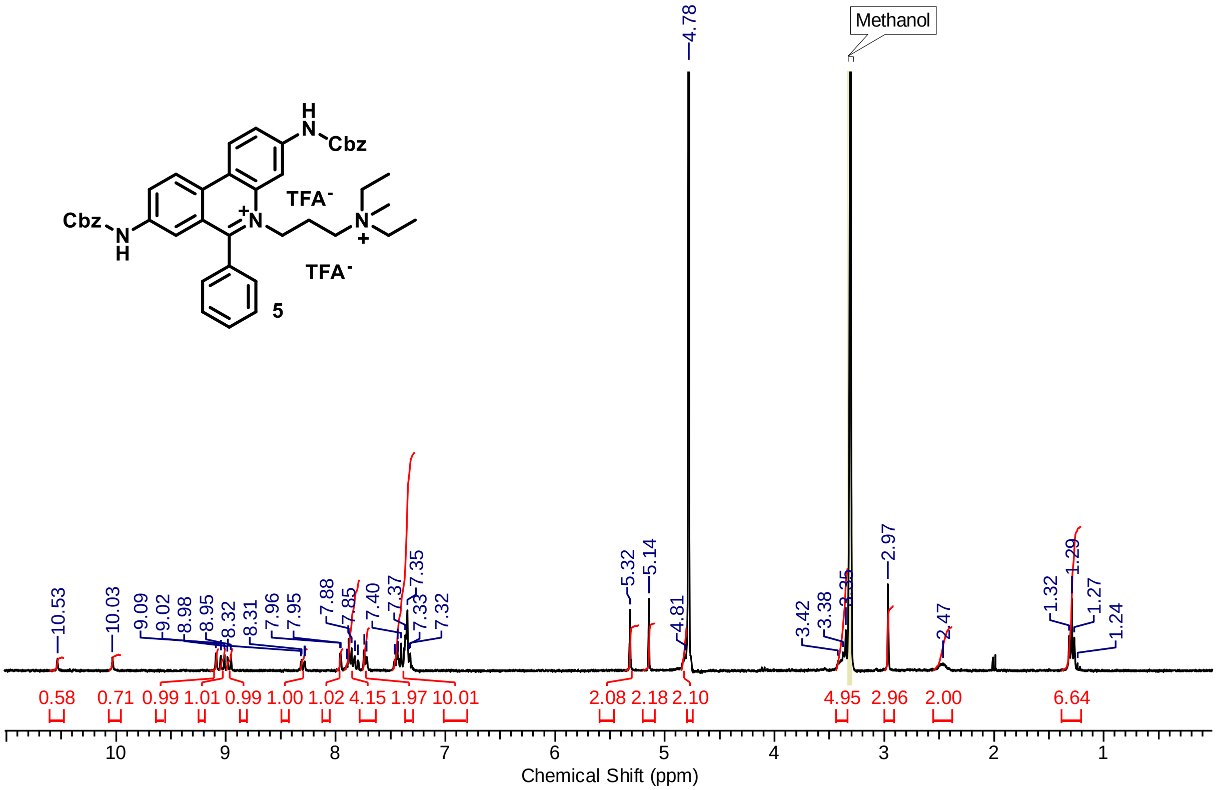

^{13}C NMR ($CDCl_3$) of compound **5**.

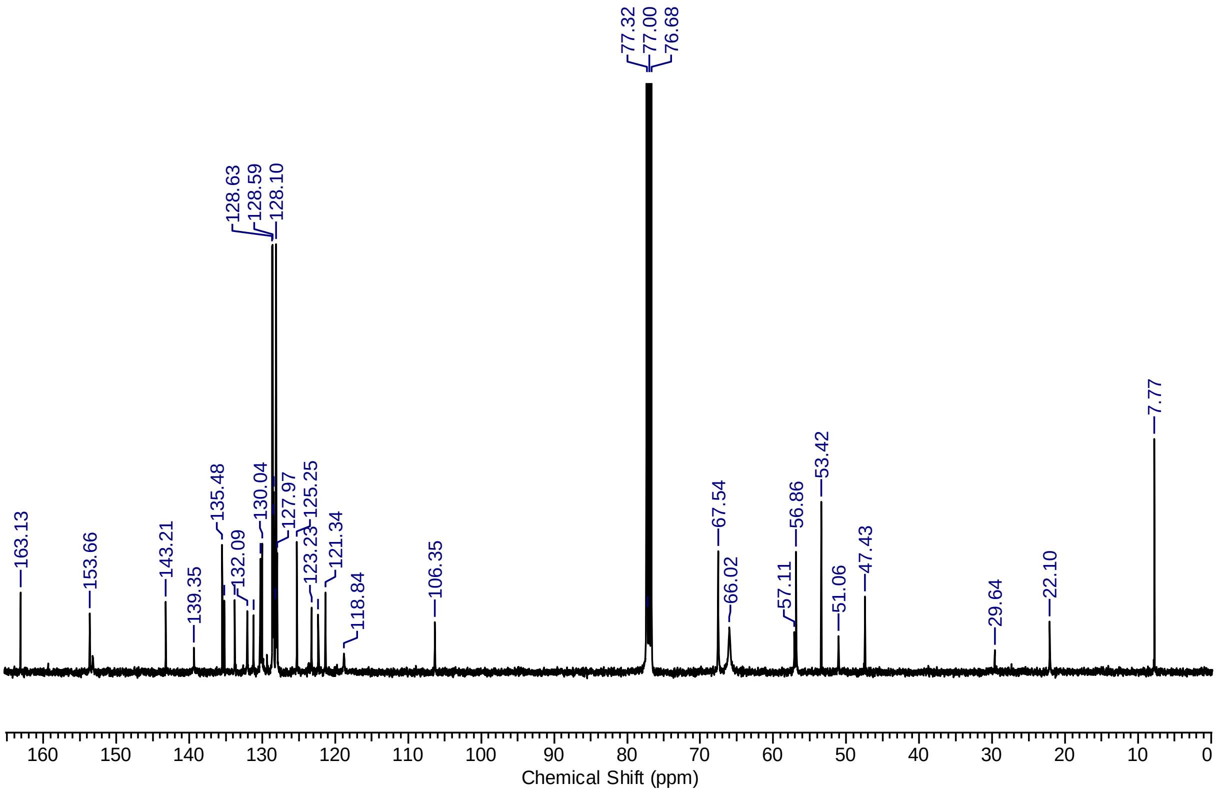

HPLC and ESI-MS of compound **5.**

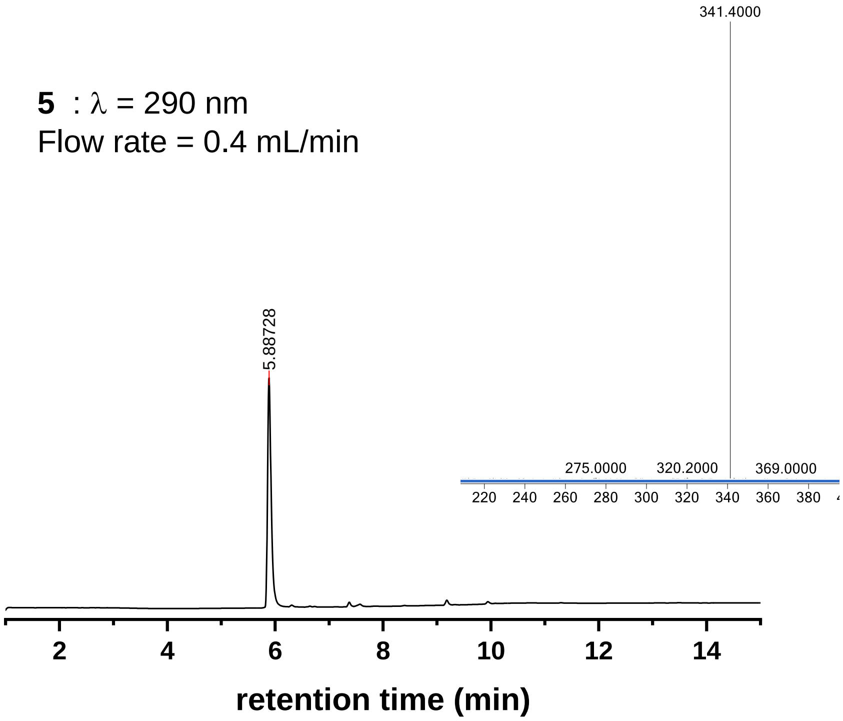

Abbreviations

2-OH-E$^+$, 2-hydroxyethidium

2-OH-TMeE$^+$,2-hydroxy-*N,N,N',N'*-tetramethylethidium

2-OH-TMePr^{++}, 2-hydroxy-*N,N,N',N'*-tetramethylpropidium

B3LYP, Becke 3-Parameter (Exchange), Lee, Yang and Parr (correlation; density functional theory)

BMIM$^+$PF$_6^-$,1-butyl-3-methylimidazolium hexafluorophosphate

CAT, catalase

CysS$^\bullet$, cysteine thiyl radicals

DCFH, dichlorodihydrofluorescein

DMSO, dimethyl sulfoxide

DPBS, Dulbecco's phosphate-buffered saline

dtpa, diethylenetriaminepentaacetate

E$^+$, ethidium

E$^+$-E$^+$, ethidium - ethidium dimer

GS$^\bullet$, glutathionyl radicals

GSH, glutathione

GSSG$^{\bullet-}$, radical cation of glutathione disulfide

HE($^\bullet$NH), aminyl radical of hydroethidine

HE, hydroethidine

HE$^{\bullet+}$, radical cation of hydroethidium

HE-E$^+$, hydroethidine - ethidium dimer

HE-HE, hydroethidine - hydroethidine dimer

HPLC, high-performance liquid chromatography

HPr$^+$, hydropropidine

HPr$^{\bullet 2+}$, radical cation of hydropropidine

HRP, horseradish peroxidase

HX, hypoxanthine

L-012, 8-amino-5-chloro-7-phenyl-pyrido[3,4-d]pyridazine-1,4(2H,3H)dione

LC/MS, liquid chromatography-mass spectrometry

MeCN, acetonitrile

MeE, methylethidine

MeE$^{\bullet+}$, radical cation of methylethidium

Mito-HE/MitoSOX Red, mitochondria-targeted hydroethidine

MitoNeoD, mitochondria-targeted superoxide probe

NAD(P)H, reduced nicotinamide adenine dinucleotide phosphate

PMA, phorbol 12-myristate 13-acetate

Pr^{++}-Pr^{++} , propidium-propidium dimer

RNS, reactive nitrogen species

ROS, reactive oxygen species

RSH, low-molecular-weight thiols

SOD, superoxide dismutase

TD-DFT, time-dependent density functional theory

$TMeE^{+}$, *N,N,N',N'*-tetramethylethidium

$TMeE^{+}$-$TMeE^{+}$, *N,N,N',N'*-tetramethylethidium - *N,N,N',N'*-tetramethylethidium dimer

TMeHE, *N,N,N',N'*-tetramethylhydroethidine

$TMeHE^{\bullet 2+}$, radical cation of *N,N,N',N'*-tetramethylhydroethidium

$TMeHPr^{+}$, *N,N,N',N'*-tetramethylhydropropidine

$TMeHPr^{\bullet 2+}$, radical cation of *N,N,N',N'*-tetramethylhydropropidine

$TMePr^{++}$, *N,N,N',N'*-tetramethylhydropropidium dication

$TMePr^{++}$-$TMePr^{++}$, *N,N,N',N'*-tetramethylpropidium dication - *N,N,N',N'*-tetramethylpropidium dication dimer

UPLC, ultra performance liquid chromatography

UV-Vis, ultraviolet/visible spectroscopy

XO, xanthine oxidase

References

1 Armstrong, D. A. *et al.* Standard electrode potentials involving radicals in aqueous solution: inorganic radicals (IUPAC Technical Report). *Pure Appl Chem* **87**, 1139-1150, doi:10.1515/pac-2014-0502 (2015).

2 Zielonka, J., Sarna, T., Roberts, J. E., Wishart, J. F. & Kalyanaraman, B. Pulse radiolysis and steady-state analyses of the reaction between hydroethidine and superoxide and other oxidants. *Archives of biochemistry and biophysics* **456**, 39-47, doi:10.1016/j.abb.2006.09.031 (2006).

3 Madej, E. & Wardman, P. The oxidizing power of the glutathione thiyl radical as measured by its electrode potential at physiological pH. *Archives of biochemistry and biophysics* **462**, 94-102, doi:10.1016/j.abb.2007.03.002 (2007).

4 Das, T. N., Dhanasekaran, T., Alfassi, Z. B. & Neta, P. Reduction potential of the tert-butylperoxyl radical in aqueous solutions. *J Phys Chem A* **102**, 280-284, doi:DOI 10.1021/jp972903t (1998).

5 Lebret, M. & Chalvet, O. Optical-Spectrum of Ethidium Intercalated into DNA - Cndo-S Study. *J Mol Struct* **37**, 299-319, doi:Doi 10.1016/0022-2860(77)80095-1 (1977).

6 Chesis, P. L., Hwang, D. R. & Welch, M. J. N-(3-[F-18]Fluoropropyl)-N-Nordiprenorphine - Synthesis and Characterization of a New Agent for Imaging Opioid Receptors with Positron Emission Tomography. *J Med Chem* **33**, 1482-1490, doi:DOI 10.1021/jm00167a031 (1990).

7 Michalski, R., Zielonka, J., Hardy, M., Joseph, J. & Kalyanaraman, B. Hydropropidine: a novel, cell-impermeant fluorogenic probe for detecting extracellular superoxide. *Free Radic Biol Med* **54**, 135-147, doi:10.1016/j.freeradbiomed.2012.09.018 (2013).

8 Zielonka, J., Zhao, H., Xu, Y. & Kalyanaraman, B. Mechanistic similarities between oxidation of hydroethidine by Fremy's salt and superoxide: stopped-flow optical and EPR studies. *Free Radic Biol Med* **39**, 853-863, doi:10.1016/j.freeradbiomed.2005.05.001 (2005).

9 Zielonka, J., Vasquez-Vivar, J. & Kalyanaraman, B. Detection of 2-hydroxyethidium in cellular systems: a unique marker product of superoxide and hydroethidine. *Nature protocols* **3**, 8-21, doi:10.1038/nprot.2007.473 (2008).

10 Chesis, P. L., Hwang, D. R. & Welch, M. J. N-(3-[F-18]Fluoropropyl)-N-Nordiprenorphine - Synthesis and Characterization of a New Agent for Imaging Opioid Receptors with Positron Emission Tomography. *J Med Chem* **33**, 1482-1490, doi:DOI 10.1021/jm00167a031 (1990).

11 Buxton, G. V., Greenstock, C. L., Helman, W. P. & Ross, A. B. Critical-Review of Rate Constants for Reactions of Hydrated Electrons, Hydrogen-Atoms and Hydroxyl Radicals (.Oh/.O-) in Aqueous-Solution. *Journal of Physical and Chemical Reference Data* **17**, 513-886, doi:Doi 10.1063/1.555805 (1988).

12 Schuler, R. H., Patterson, L. K. & Janata, E. Yield for the scavenging of hydroxyl radicals in the radiolysis of nitrous oxide-saturated aqueous solutions. *The Journal of Physical Chemistry* **84**, 2088-2089, doi:10.1021/j100453a020 (1980).

13 Neta, P., Huie, R. E. & Ross, A. B. Rate Constants for Reactions of Inorganic Radicals in Aqueous-Solution. *Journal of Physical and Chemical Reference Data* **17**, 1027-1284, doi:Doi 10.1063/1.555808 (1988).

14 Hayon, E. & Simic, M. Absorption spectra and kinetics of the intermediate produced from the decay of azide radicals. *Journal of the American Chemical Society* **92**, 7486-7487, doi:10.1021/ja00728a049 (1970).

15 Alfassi, Z. B., Harriman, A., Huie, R. E., Mosseri, S. & Neta, P. The redox potential of the azide azidyl couple. *J Phys Chem-Us* **91**, 2120-2122, doi:DOI 10.1021/j100292a029 (1987).

16 Ram, M. S. & Stanbury, D. M. Electron-transfer reactions involving the azidyl radical. *J Phys Chem-Us* **90**, 3691-3696, doi:DOI 10.1021/j100407a042 (1986).

17 Buxton, G. V., Wood, N. D. & Dyster, S. Ionization-constants of $^{\bullet}OH$ and $HO_2^{\bullet}$ in aqueous-solution up to 200°C - a pulse-radiolysis study. *Int J Radiat Biol* **53**, 996-997 (1988).

18 Perrin, D. D., International Union of, P., Applied, C. & Commission on Equilibrium, D. *Ionisation constants of inorganic acids and bases in aqueous solution*. (Pergamon Press, 1982).
19 Grätzel, M., Henglein, A. & Taniguchi, S. Pulsradiolytische beobachtungen über die reduktion des NO3−-Ions und über bildung und zerfall der persalpetrigen säure in wäßriger lösung. *Berichte der Bunsengesellschaft für physikalische Chemie* **74**, 292-298 (1970).
20 Benderskii, V., Krivenko, A., Ponomarev, E. & Fedorovich, N. Rate constants of protonation of the ion radical NO_3^{2-}. *Elektrokhimiya*, 1435-1439 (1987).
21 Misik, V., Mak, I. T., Stafford, R. E. & Weglicki, W. B. Reactions of captopril and epicaptopril with transition metal ions and hydroxyl radicals: an EPR spectroscopy study. *Free radical biology & medicine* **15**, 611-619 (1993).
22 Schoneich, C., Bonifacic, M. & Asmus, K. D. Reversible H-atom abstraction from alcohols by thiyl radicals: determination of absolute rate constants by pulse radiolysis. *Free radical research communications* **6**, 393-405 (1989).
23 Micic, O. I. & Cercek, B. Diffusion-controlled reactions in mixed solvents. *The Journal of Physical Chemistry* **81**, 833-837, doi:10.1021/j100524a006 (1977).
24 Afanassiev, A. M., Okazaki, K. & Freeman, G. R. Effect of solvation energy on electron reaction rates in hydroxylic solvents. *The Journal of Physical Chemistry* **83**, 1244-1249, doi:10.1021/j100473a003 (1979).
25 Meisel, D., Matheson, M. S., Mulac, W. A. & Rabani, J. Transients in the flash photolysis of aqueous solutions of tris(2,2'bipyridine)ruthenium(II) ion. *The Journal of Physical Chemistry* **81**, 1449-1455, doi:10.1021/j100530a004 (1977).
26 Hart, E. J., Gordon, S. & Thomas, J. K. Rate Constants of Hydrated Electron Reactions with Organic Compounds1. *The Journal of Physical Chemistry* **68**, 1271-1274, doi:10.1021/j100788a001 (1964).
27 Balkaş, T. I. The radiolysis of aqueous solutions of methylene chloride. *International Journal for Radiation Physics and Chemistry* **4**, 199-208 (1972).
28 Köster, R. & Asmus, K.-D. Die Reduktion von Tetrachlorkohlenstoff durch hydratisierte Elektronen, H-Atome und reduzierende Radikale/The Reduction of Carbon Tetrachloride by Hydrated Electrons, H-Atoms, and Reducing Radicals. *Zeitschrift für Naturforschung B* **26**, 1104-1108 (1971).
29 Willson, R. & Slater, T. in *Fast processes in radiation chemistry and biology. Proceedings of the fifth LH Gray Conference, held at the University of Sussex, 10-14 September 1973.*
30 Brault, D. *et al.* One-electron reduction of ferrideuterioporphyrin IX and reaction of the oxidized and reduced forms with chlorinated methyl radicals. *Journal of the American Chemical Society* **102**, 1015-1020, doi:10.1021/ja00523a018 (1980).
31 Marchaj, A., Kelley, D. G., Bakac, A. & Espenson, J. H. Kinetics of the reactions between alkyl radicals and molecular oxygen in aqueous solution. *The Journal of Physical Chemistry* **95**, 4440-4441, doi:10.1021/j100164a051 (1991).
32 Emmi, S., Beggiato, G., Casalbore, G. & Fuochi, P. in *Proceedings of the 5th Tihany symposium on radiation chemistry held at Siofok, 19-24 Sep 1982. Vols. 1, 2.*
33 Monig, J., Bahnemann, D. & Asmus, K. D. One electron reduction of CCl4 in oxygenated aqueous solutions: a CCl3O2-free radical mediated formation of Cl- and CO2. *Chemico-biological interactions* **47**, 15-27 (1983).
34 Adams, G. E. & Willson, R. L. Pulse radiolysis studies on the oxidation of organic radicals in aqueous solution. *Transactions of the Faraday Society* **65**, 2981-2987, doi:10.1039/TF9696502981 (1969).
35 Gordon, S., Hart, E. J., Matheson, M. S., Rabani, J. & Thomas, J. K. Reaction Constants of the Hydrated Electron. *Journal of the American Chemical Society* **85**, 1375-1377, doi:10.1021/ja00893a002 (1963).

36 Alfassi, Z. B. & Schuler, R. H. Reaction of Azide Radicals with Aromatic-Compounds - Azide as a Selective Oxidant. *Journal of Physical Chemistry* **89**, 3359-3363, doi:DOI 10.1021/j100261a040 (1985).

37 Saini, R. D. & Bhattacharyya, P. K. Flash Photolytic Investigations on the Reaction between Pu(Vi) and Co3- in Alkaline-Medium. *Radiat Phys Chem* **27**, 189-193 (1986).
